# Zinc Metallacarborane Chemistry

**DOI:** 10.1021/acs.inorgchem.6c00184

**Published:** 2026-02-06

**Authors:** Kerry R. Flanagan, Chloe L. Johnson, Joe C. Goodall, Claire L. McMullin, Andrew L. Johnson

**Affiliations:** Department of Chemistry, 1555 University of Bath, Claverton Down, Bath BA2 7AY. U.K.

## Abstract

This work presents a new family of zincacarborane complexes synthesized from ZnMe_2_ and [C_2_B_9_H_13_], using neutral two-electron donor ligands: *N*-heterocyclic carbenes (NHCs) yield the first *closo*-12-vertex half-sandwich zincocenes, [(NHC)­Zn­(C_2_B_9_H_11_)] (**1**–**3**), while bulkier NHCs form slipped bis-dicarbollide salts (**4**–**5**). Use of pyridine leads to the macropolyhedral dimer (**6**) with a planar {Zn_2_B_2_} motif, and triphenylphosphine gives a V-shaped η^3^-borallyl complex (**7**). Structures have been confirmed by single-crystal X-ray diffraction and NMR spectroscopy. Computational studies (DFT and QTAIM) show predominantly ionic Zn–dicarbollide bonding with notable polar covalent character. Apparent Zn···Zn interactions are weak electrostatic contacts, and metal–ligand bonding is exclusively to boron atoms. Together, these findings broaden the structural and electronic landscape of zincacarboranes, challenge assumptions about d^10^ metal–carborane bonding, and offer a new platform for exploring group 12 metallacarborane reactivity.

## Graphical abstract

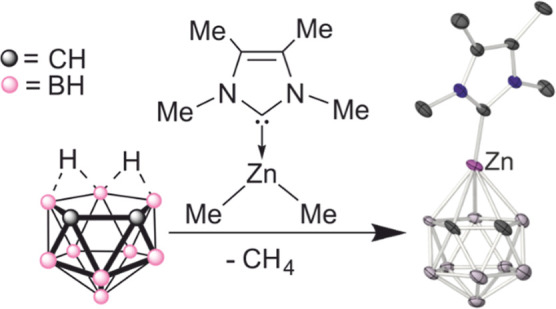

## Introduction

Not long after the discovery of ferrocene,^1^ the first structurally characterized metal-carborane sandwich complexes containing the anion [*nido*-1,2-C_2_B_9_H_11_]^2–^, or dicarbollide anion, was reported by Hawthorne et al.^2^ and opened-up the development of a new class of sandwich complexes known as metallacarboranes.^3^ The σ^2^π^4^ arrangement of the frontier molecular orbitals of the [*nido*-1,2-C_2_B_9_] dianion is remarkably similar to [C_5_H_5_]^−^ (Cp^–^),^4^ making it capable of mimicking the η^5^-, η^3^- and η^1^-bonding behavior of the Cp^–^ anion to form sandwich and half sandwich complexes. The interaction of zinc­(II) with charged conjugated π-systems, such as cyclopentadienyl and allyl groups, is now common.^5^ However, these Zn–C_π_-interactions are considered special cases because the negative charge of the ligand is delocalized over the π-system and Coulombic interactions play a significant role in metal–ligand interactions.^5e^

In general, metal-(η^5^-carborane) bonding is comparable to metal-(η^5^-Cp) bonding, with the [*nido*-C_2_B_9_] dianion displaying similar, but subtlty different bonding to that found in metal-Cp analogues.^3a,6^ Metal-(η^5^-carborane) bonding is also considerably stronger, than the corresponding metal-Cp systems, due to the greater covalent character in metal–dicarbollide versus metal–hydrocarbon binding. However, the C and B atom of the open {C_2_B_3_} face do not contribute equally to the frontier molecular orbitals, resulting in a strong σ-bonding component from the boron atoms and stronger M–B bonding compared to M–C bonding.^7^ As a result, metal coordination to the open {C_2_B_3_} face of the dicarbollide ligand is uneven such that the metal atoms often lie closer to the boron atoms than the carbon atoms.^7b^

While “classic” {η^5^-C_2_B_9_} complexes, in which metallic residues that are isolobal with {BH}^2+^ fragments^8^ can bond in an η^5^-fashion to the face completing the closo-metallacarborane geometry are common,^4b^ as metallacarborane chemistry developed it became obvious that not all metal centers formed *closo*-metallacarboranes.^4b,9^ Subsequently a number of compounds in which the binding of the dicarbollide unit to the metal centers varies between pentahapto (structure **I**, Figure 1), through degrees of increasingly less involvement (**II** to **VI**), to a looser association to the {C_2_B_3_} face as the nature of the metal atom changes from early to midtransition series elements,^10^ to the late transition series elements^7a,11^ and onward to the post-transition elements, such as tin (**IV**),^12^ mercury (**V**)^13^ and thallium (**VI**).^9^ Displacement of the metal atom away from symmetric π-bonding to the open face of a dicarbollide ligand is commonly observed in single cage metallacarboranes with a slip distortion, “Δ”, defined as the distance the metal atom is displaced from a position above the centroid of the lower pentagon of five B atoms for icosahedral species. The related parameter, “*h*”, defined as the perpendicular displacement of the metal atom above the least-squares plane through the lower belt of B atoms (see Figure 2) has also been used as a slip parameter.

**Figure 1:**
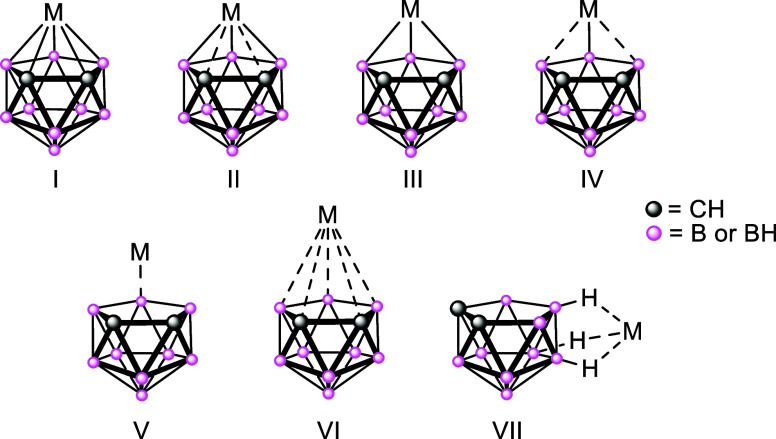
Examples of the various coordination modes of the {7,8-C_2_B_9_H_11_} ligand unit with metals.

**Figure 2:**
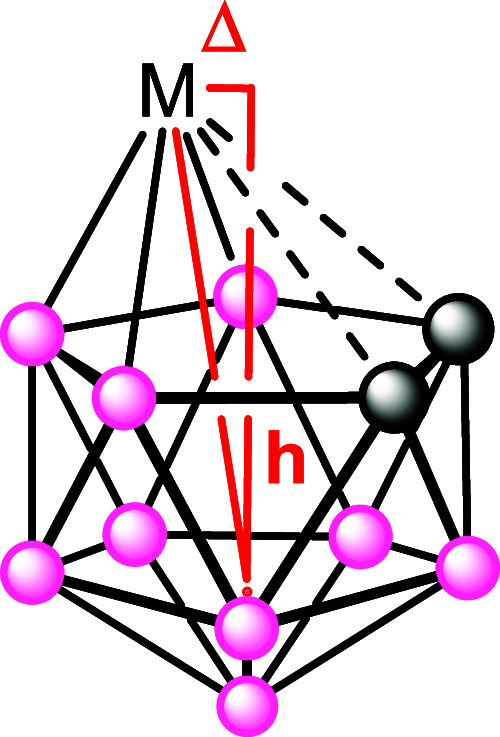
Pictorial representation of the structural “slip” parameters Δ and *h*.

It should also be noted that a number of *exo*-*nido*-metallacarboranes (**VII**), in which metal containing fragments coordinate to {B–H} groups around the exterior of the [*nido*-1,2-C_2_B_9_H_11_] ligand, have also been identified.^14^

Consequently, metallacarborane chemistry can demonstrate very different structural chemistries to their cyclopentadienyl analogues. For example, the zincacarborane system (**VIII**, Figure 3) formed from reaction of ZnMe_2_ with [Me_3_NH]­[*nido*-7,8-C_2_B_9_H_12_], is novel as both the first characterized zincacarborane, and in its unprecedented molecular structure.^6a^ The macropolyhedral dimer, *commo*-[(*nido*-C_2_B_9_H_11_)­Zn·NMe_3_]_2_, consists of two [(*nido*-C_2_B_9_H_11_)] ligands coordinated to a central {(Me_3_N)­Zn···Zn­(NMe_3_)} unit [Zn···Zn = 2.800(1) Å], to form a highly unusual planar, diamond-shaped central {Zn_2_B_2_} arrangement, via 3-center B···Zn···B interactions. Such a structure does indeed seem less extraordinary when it is recalled that Zn^2+^ possesses a full 3d^10^ orbital with only one 4s, and three 4p orbitals available for bonding; as such a sp^3^ hybridized {L–Zn}^2+^ fragment should act as a formally isolobal, isoelectronic analogue of {BH}^2+^.^15^

**Figure 3:**
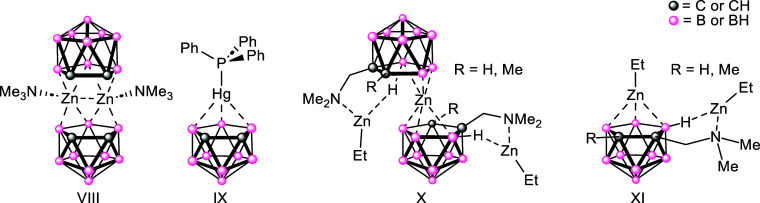
Molecular structures of the known selected group 12 metallacarborane systems (**VIII**–**XI**). (NB {N­(H)­CH_2_Ph} derivatives of **X** and **XI** are also known).

It should be noted that, all things being equal, the isoelectronic mercury­(II) complex, [Ph_3_P·HgC_2_B_9_H_11_] (**IX**) should also be a *closo*-metallacarborane system, isostructural and isoelectronic with *closo*-C_2_B_10_H_12_. However, despite the {Ph_3_P–Hg^2+^} and {HB^2+^} units being formally isolobal, [Ph_3_P·HgC_2_B_9_H_11_] has a “slipped”-structure with a η^3^-coordination between {Ph_3_P–Hg} and {C_2_B_9_H_11_},^13a^ presumably a result of reduced s-p orbital mixing in the Hg^2+^ species.

Despite an intervening 26 years since the first zincacarborane was reported,^6a^ sandwich or half-sandwich metallacarboranes of group 12 metals continue to be rare, with only a two added to the literature (**X** and **XI**) (Figure 3), neither of which possess η^5^-coodination of the {C_2_B_9_H_11_} ligand to the metal.^13a,16^

To date 12-vertex *closo*-metallacarboranes of Zn have yet to be isolated despite attempts made by some researchers.^6a,16^ While the reasons for the paucity of examples is not clear, it may be attributed first to the number of bonding interactions between the metal and the {*nido*-C_2_B_9_H_11_} dianion decreasing as the electron density on the metal increases (i.e., Zn^2+^: d^10^); and second as the full d-orbitals are unavailable for bonding, this promotes looser π-interactions with the dicarbollide ligand. Similarly, no related alkaline-earth dicarbollide compounds have been reported to date.

In this contribution, we present the synthesis, structure, and computational analysis of a family of half-sandwich mono dicarbollyl-zincocenes complexes **1–3** and **6** and the full-sandwich *bis*-dicarbollyl zincocenes **4–5** and **7**.

## Results and Discussion

The direct reaction of ZnR_2_ or ZnX_2_ (R = Alkyl or Aryl, X = Halide, alkoxide, amide, carboxylate) systems with neutral 2-electron donor species (L), such as NHCs, phosphines or amine based ligands is a well-established pathway for the production of [Zn–L] adducts.^17^ The addition of the *N*-Heterocyclic carbenes (**a**–**e**) at 0 °C to a toluene solution of ZnMe_2_ resulted in the in situ formation of NHC adducts [{(NHC}­ZnMe_2_] (**a**: R = R′ = Me; **b**: R = ^*i*^Pr, R′ = Me; **c**: R = Dipp (2,6-diisopropylphenyl), R′ = H; **d**: R = ^*t*^Bu, R′ = Me; **e**: R = Ad, R′ = Me) (Scheme 1), as confirmed by ^1^H and ^13^C­{^1^H} NMR spectroscopy.^18^ Subsequent reaction of the NHC adducts **a–c**, with one equivalent of the dicarbollyl acid 7,8-C_2_B_9_H_13_ in toluene yielded *closo*-12 vertex, half sandwich, zincocenes [{NHC}­Zn­(η^5^-C_2_B_9_H_9_)] (**1**: NHC = *a*; **2**: NHC = *b*; **3**: NHC = *c*). It should be noted that the addition of same NHCs to C_2_B_9_H_13_, results in the formation of the salts [NHC–H]­[C_2_B_9_H_12_] respectively.^19^ Subsequent reaction with ZnMe_2_ yielded **1**, **2** and **3** in comparable yield.

**Scheme 1:**
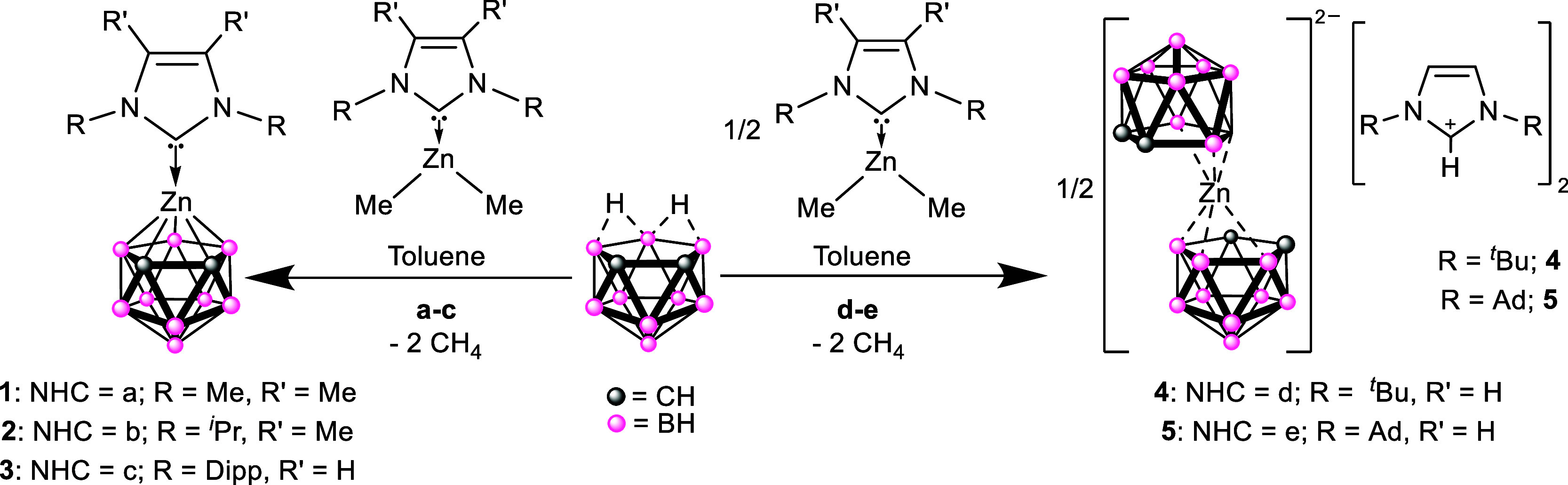
Synthesis of the 12-Vertex Half-Sandwich *closo*-Zincacarborane Complexes **1–3** and the Full-Sandwich *commo*-Zincacarborane Salts **4–5**

Compounds **1–3** provided good quality ^1^H, ^13^C­{^1^H}, ^11^B and ^11^B­{^1^H} NMR spectra in CD_2_Cl_2_, which were consistent with the suggested formulations (i.e., 1:1 ratio of the NHC ligand to the {C_2_B_9_H_11_} unit). Resonances associated with either free ZnMe_2_ or NHC–ZnMe_2_ adducts,^18c,20^ are absent from the spectra. This along with carbene carbon resonances found at 160.2 (**1**), 157.9 (**2**) and 168.4 ppm (**3**), which agree with those observed for previously reported NHC complexes of zinc,^18a,21^ is consistent with the formulation of the proposed products.

Colorless single crystals suitable for X-ray diffraction analysis of compounds **1–3** were obtained from concentrated dichloromethane solutions of **1**, **2** and **3** respectively, stored at −28 °C, in good yields (**1**, 87%; **2**, 80%; **3**, 82%). The molecular structures of **1**, **2** and **3** (Figure 4), provide unambiguous evidence that the {C_2_B_9_} moieties are capped with a zinc–NHC fragment, resulting in the formation of a *closo*-12-vertex species. Selected bond lengths and angles for **1**–**3** are provided in Table 1.

**Figure 4:**
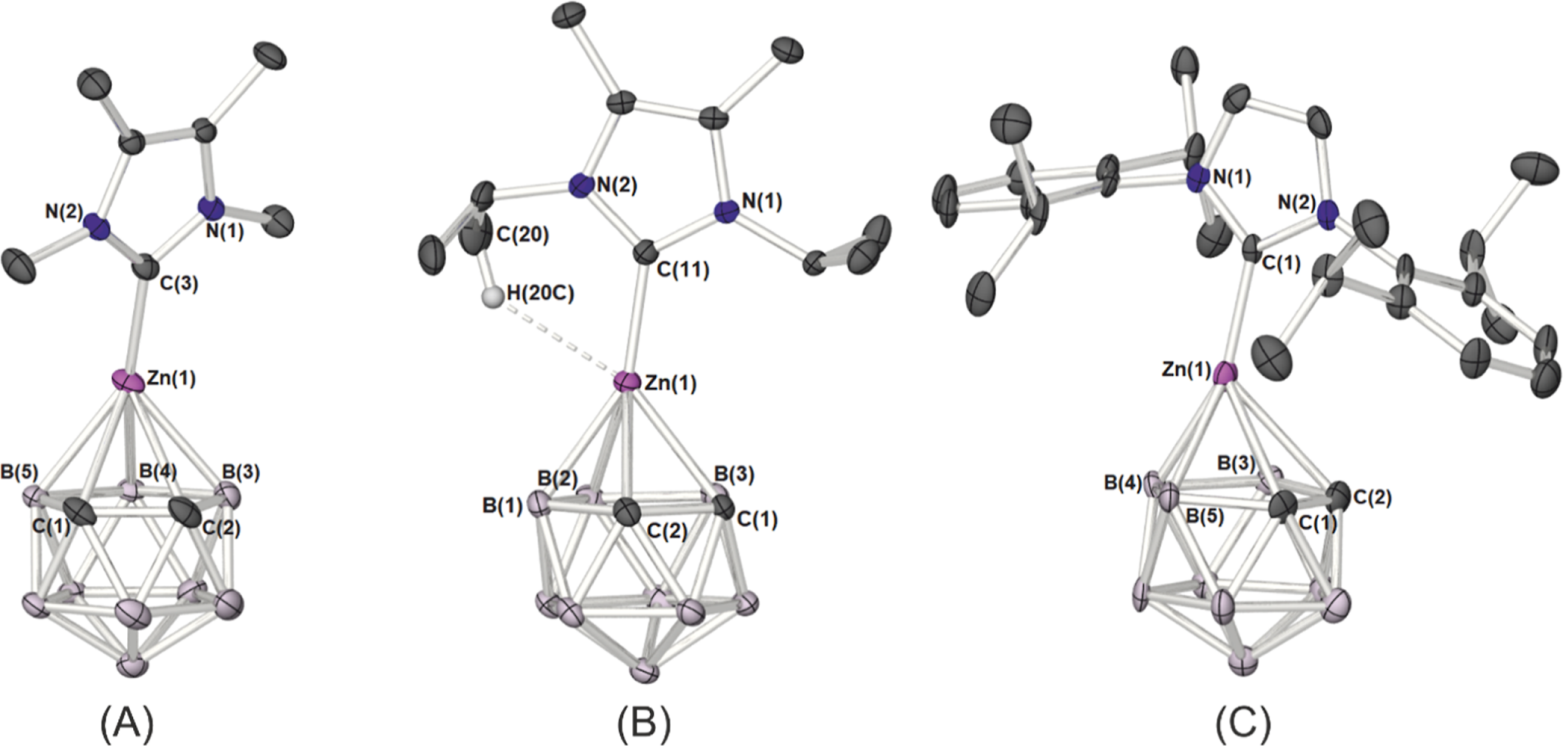
Molecular structures of the zincacarborane **1** (A), **2** (B) and **3** (C): all hydrogen atoms have been omitted for clarity. Thermal ellipsoids are shown at 50% probability.

**Table 1:** Selected Atomic Distances (Å) and Bond Angles (°) for Complexes **1**–**3**

	1	2	3
Zn(1)–C(3)	1.962(8)	1.954(1)	1.958(6)
Zn(1)–C(1)	2.44(1)	2.449(1)	2.407(8)
Zn(1)–C(2)	2.47(1)	2.471(1)	2.425(8)
Zn(1)–B(3)	2.26(2)	2.215(2)	2.203(9)
Zn(1)–B(4)	2.111(9)	2.093(1)	2.076(8)
Zn(1)–B(5)	2.22(1)	2.248(2)	2.203(8)
C(1)–C(2)	1.56(1)	1.566(2)	1.57(1)
Zn(1)–{C_2_B_3_}_Cent_	1.790(9)	1.783(2)	1.741(9)
C(3)–Zn(1)–{C_2_B_3_}_Cent_	146.17(9)	155.64(9)	155.44(8)
C(3)–Zn(1)–{C_2_B_3_}_Cent_–{C_2_}_Cent_	15.69(9)	6.53(9)	–4.61(8)°
N(1)–C(3)–N(2)	106.0(8)	105.8(1)	105.1(5)
Δ/*h*	0.45/3.274	0.46 Å/3.233	0.44/3.202

Complexes **1**, **2** and **3** all adopt a half-sandwich configuration characterized by interaction between the Zn atom and {η^5^-C_2_B_3_} (see Table 1) comparable to definitively η^5^-interactions between dicarbollide ligands and metals of comparable radii.^22^ These [Zn-η^5^-C_2_B_3_] are the shortest among all the zincacarboranes reported and shorter than reported η^5^Cp–Zn and η^5^Cp*–Zn interactions (1.90–1.93 Å).^23^ The Zn–NHC interactions [1.954(1)–1.962(8) Å] and NHC–Zn–{C_2_B_3_}_cent_ arrangements, which tend toward linearity [**1**: 146.17(9)°; **2**: 155.64(9)°; **3**: 155.44(8)°], result in overall pseudo two-coordinate, or distorted linear “pogo-stick” like molecules.

Despite the diverse range of steric requirements for the NHC ligands used here, there is little change in the range of C···Zn bond lengths,^24^ with Zn–C_NHC_ bonds in **1–3** ca. ∼1.96 Å [**1**: 1.962(8) Å; **2**: 1.954(1) Å; **3**: 1.9584(6) Å]. These values are collectively shorter than those observed in the neutral complexes, (IMe_4_)­Zn­(η^1^-C_5_Me_5_)_2_ [2.022(3) Å],^25^ (I^*i*^Pr_2_Me_2_)­ZnH_2_ [2.074(2) Å][Bibr cit21b] and (IPr)­ZnMe_2_ [2.113(2) Å][Bibr cit18c], and more comparable to those observed in cationic Zn–NHC containing species^26^ (∼1.93–2.00 Å), reflecting the higher Lewis acidity of the zinc atoms in **1–3**.

The Zn–{C_2_B_3_} π-interactions are asymmetric and contrast to the more symmetric Zn–Cp* interactions, in systems such as [Cp*–Zn–Me],^23^ [Cp*–Zn–Mes][Bibr ref23] and [Cp*–Zn–C_6_F_5_],^5g^ due to the nonuniform {C_2_B_3_} arrangement.^4a^ In **1**, the Zn(1)–B(4) bond length is measured to be 2.111(9) Å, such that the slip-parameter, Δ, is calculated to be 0.44 Å (*h* = 3.274 Å). These values indicate a shorter, stronger interaction between the Zn atom and the dicarbollide ligand than those observed in the slipped η^3^-zincacarborane reported by Lee et al.,^16^ [2.158(1) Å; Δ = 0.548 Å, *h* = 3.365 Å] and fall into the range observed in *closo*-icosahedral metallacarboranes.^22b,27^ Concomitantly, the atomic distances between Zn(1) and B(3) [2.26(2) Å] and B(5) [2.11(1) Å], are both longer than the Zn(1)–B(4) interaction, and are markedly shorter than the Zn(1)–C(1)/C(2) [2.44(1)/2.47(1) Å] distances. Despite this, both Zn–C and Zn–B are smaller than the sum of the van der Waals radii of Zn, C and B, thus, π-interactions between Zn and the {C_2_B_3_} face can be inferred (vide infra). These general structural trends are continued across **2** and **3** (Figure 4), with similar Zn–C and Zn–B interactions between the zinc atom and the {C_2_B_3_} face of the dicarbollyl ligand, and similar slip-parameters [**1**: Δ = 0.45, *h* = 3.27 Å; **2**: Δ = 0.46, *h* = 3.23 Å; **3**: Δ = 0.44, *h* = 3.20 Å] despite the relative differences in steric properties of the three NHC-ligands.^28^ These observations are in stark contrast to the related Ph_3_P-supported mercuracarboranes all of which adopt structures with slipped η^3^-{C_2_B_3_} geometries.^13,29^

In **2**, a feature of note is the unusual ^*i*^Pr orientation of one of the substituents on the NHC ligand. Typically, the methyl groups of the ^*i*^Pr substituent are always directed to the back of the carbene and not toward the metal center as seen in **2**, indicating the presence of a indicative of a weak {C–H···Zn} H-bonding interaction [Zn(1) ··C(20) = 3.36826(3)­Å; Zn(1) ··H­(20c) = 2.66762(3)­Å; Zn(1) ··H­(20c)–C(20) = 128.6925(7)°] which is the only example of this being isolated in the whole of the CSD.

In the case of **1**, the coordination sphere of the Zn atom is further augmented by two long intermolecular, secondary B–H···Zn interactions (Figure 5) [Zn(1)···B(4)­A = 3.39(2) Å; Zn(1)···H(4)­A = 2.76(2) Å; Zn(1)···B(5)­A = 2.79(3) Å; Zn(1)···H(5)­A = 3.39(2) Å; Zn(1)–H(4)­A-B(4)­A = 109.30(2)°; Zn(1)–H(5)­A-B(5)­A = 110.84(2)°]. These interactions result from the two adjacent Zn-dicarbollide units, and are facilitated by the coplanarity of the NHC–{CN_2_C_2_} ring and the {Zn(1)–B(5)–B(6)} triangle [angle between planes = 7.77(7)°], with a distance between the {Zn(1)–B(5)–B(6)} and {Zn(1)­A–B(5)­A–B(6)­A} planes of 3.25(2) Å. As a result of these B–H···Zn interactions, the coordination geometry about the zinc atom in **1** can be considered to be a pseudo four coordinate, distorted-tetrahedra. This contrasts with both **2** and **3**, which show no evidence in the solid state of additional intermolecular interactions between Zn and adjacent {C_2_B_9_} moieties.

**Figure 5:**
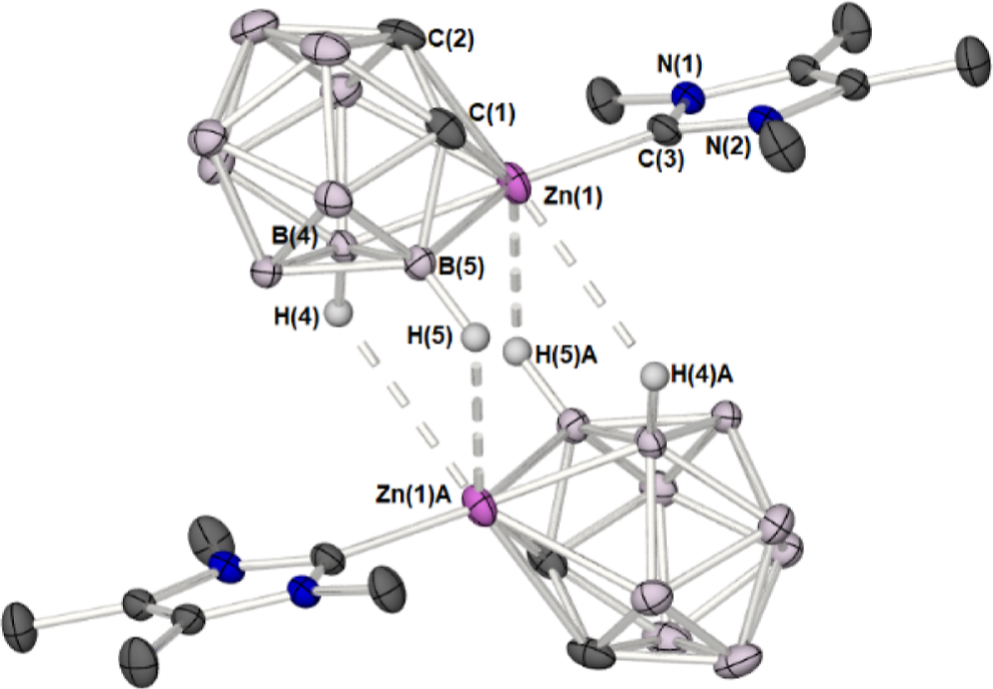
Molecular structures of **1** showing the stacking of molecules in adjacent asymmetric unit. Hydrogen atoms have been omitted for the sake of clarity. Thermal ellipsoids are shown at 50% probability. Symmetry equivalent atoms are generated by the operator: *A* = 2 – *X*, −*Y*, 1 – *Z*.

In the case of both **1** and **2** the presence of additional noncovalent intramolecular B–H···Zn interactions and intermolecular C–H···Zn interactions reflect the high Lewis-acidity of the Zn^2+^ ion.

Reaction of the ^*t*^Bu and adamantyl NHC–ZnMe_2_ adducts **1d** and **1e**, with the one equivalent of the dicarbollyl acid 7,8-C_2_B_9_H_13_ in toluene yielded the zincacarborane systems [(H–NHC)]_2_ [Zn­(η^3^-C_2_B_9_H_11_)_2_] (**4**: NHC = *d*; **5**: NHC = *e*) (Scheme 1). The formation of Zn-dicarbollide species can be most clearly observed in the ^13^C­{^1^H} NMR spectra through the appearance of a characteristic resonance observed at δ = 129.8 and 131.1 ppm respectively, corresponding to the imidazolium cations. Both reactions were performed on an NMR scale due to the limited availability of the NHC ligands, as such reaction of ZnMe_2_ with the preformed [NHC–H]­[C_2_B_9_H_12_] salts was not attempted.

Colorless single crystals of **5** suitable for X-ray diffraction analysis were obtained from concentrated dichloromethane solutions stored at −28 °C, in high yields (91%). Crystals of **4** suitable for X-ray diffraction analysis could not be obtained.

The molecular structure of **5** is shown in Figure 6, with selected interatomic distances and angles are presented in the caption. Single-crystal X-ray analysis revealed that zincacarborane salt **5** crystallizes in the *P-*1 space group, in which the Zn(1) atom is located about a crystallographic inversion center (Figure 6) such that the asymmetric unit contains half of the [Zn­(η^3^-C_2_B_9_H_11_)_2_]^2–^ dianion alongside a single imidazolium cation and two molecules of disordered dichloromethane. The central Zn(1) atom of the [Zn­(η^3^-C_2_B_9_H_9_)_2_]^2–^ anion possesses a highly distorted six-coordinate geometry in which the zinc atom is coordinated to the boron atoms of the dicarbollyl ligand in an η^3^-fashion to form a bis-η^3^-borallyl complex, with a distance between the two {C_2_B_3_} planes of the dicarbollyl ligands, i.e., {C_2_B_3_}_pln_···{C_2_B_3_}_pln_, of 3.746(3) Å. The slipped {η^3^-B_3_} bonding geometry of the [Zn­(η^3^-C_2_B_9_H_9_)_2_]^2–^ dianion is reflected in the short Zn–B [2.056(2)–2.392(2) Å] and long Zn–C distances [2.775(3)–2.737(2) Å], which exceed the sum of covalent radii for Zn and C (ca. 1.98 Å), resulting in a slip-parameter, Δ, calculated to be 0.8 Å [*h* = 3.37 Å; Zn(1)–{C_2_B_3_}_cent_ = 2.008(3) Å; Zn–{C_2_B_3_}_pln_ = 1.873(3) Å]. These values are comparable to identifiable slipped bis-dicarbollide complexes such as the slipped η^3^-zincacarborane reported by Lee et al.,^16^ as well as cupra- and aura-*bis*dicarbollide complexes, [Cu­(η^3^-C_2_B_9_H_11_)_2_]^2–^ (Δ = 0.6 Å *h* = 3.45 Å)^30^ and [Au­(η^3^-Me_2_C_2_B_9_H_9_)_2_]^−^ [Δ = 0.74 Å *h* = 3.38 Å].^31^

**Figure 6:**
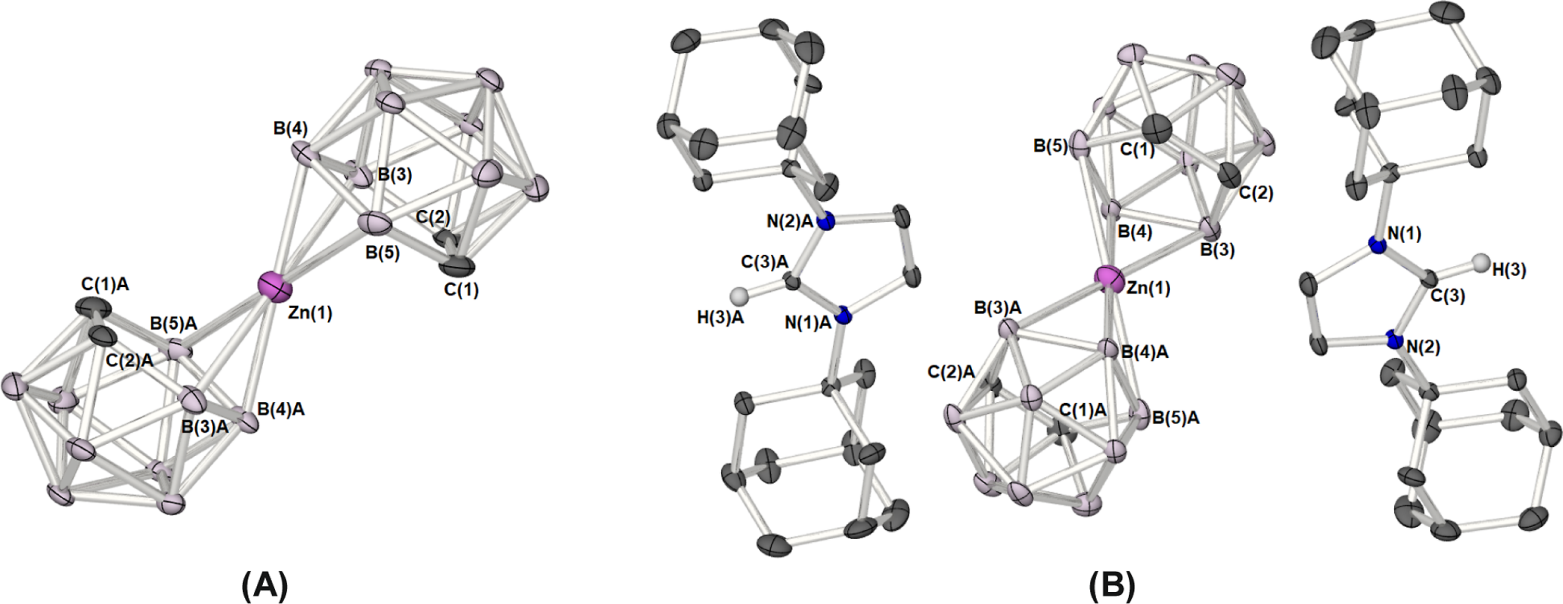
(A) Molecular structure of the of the anion [Zn­(η^3^-C_2_B_9_H_11_)_2_]^2–^ in **5**, showing the connectivity between the zinc atom and the dicarbollide cage. (B) Molecular structure of the zincacarborane salt [HC­(NAd)_2_(CMe)_2_]_2_ [Zn­(η^3^-C_2_B_9_H_11_)_2_] (**5**) (50% probability ellipsoids). Hydrogen atoms, and solvent of crystallization (CCl_2_H_2_) have been omitted for clarity. The symmetry equivalent atoms are generated by the operator: *A* = −*X*, 1 – *Y*, 1 – *Z*. Selected atomic distances (Å) and bond angles (°). **5**: Zn(1)–C(1) 2.775(3); Zn(1)–C(2) 2.737(3); Zn(1)–B(3) 2.302(2); Zn(1)–B(4) 2.056(2); Zn(1)–B(5) 2.392(2); C(1)–C(2) 1.562(2); Zn(1)–{C_2_B_3_}_cent_ 2.008(3); B(4)–Zn(1)–B(4)­A 180.0°; Δ = 0.8 Å, *h* = 3.373 Å.

From an electron counting perspective, such slipped sandwich complexes typically contain electron rich metal ions with d^8^- or d^9^-electron configurations and between 26 and 27 skeletal electron pairs (SEP). Applying the *mno*-rule to a macropolyhedral boranes, such as **5**, reveals a 28 SEP system and an excess of two electron pairs beyond that required for stability.^32^

As part of our study into the chemistry of C_2_B_9_H_13_ and ZnMe_2_, we also decided to investigate the reaction chemistry in the presence of both pyridine and triphenylphosphine, as alternative 2-electron donor ligands to the NHCs investigated here.

Direct reaction of C_2_B_9_H_13_ with one equivalent of pyridine in toluene resulted in immediate formation and precipitation of the pyridinium salt [C_5_H_5_NH]­[C_2_B_9_H_12_]. Subsequent addition of one equivalent of ZnMe_2_ resulted dissolution of the solids and formation of a clear colorless solution from which colorless crystals of **6** were obtained at −28 °C (Scheme 2). During our studies, we also investigated the addition of C_2_B_9_H_13_ to a 1:1 mixture of pyridine and ZnMe_2_, the product of which showed no discernible difference to **6**.

**Scheme 2:**
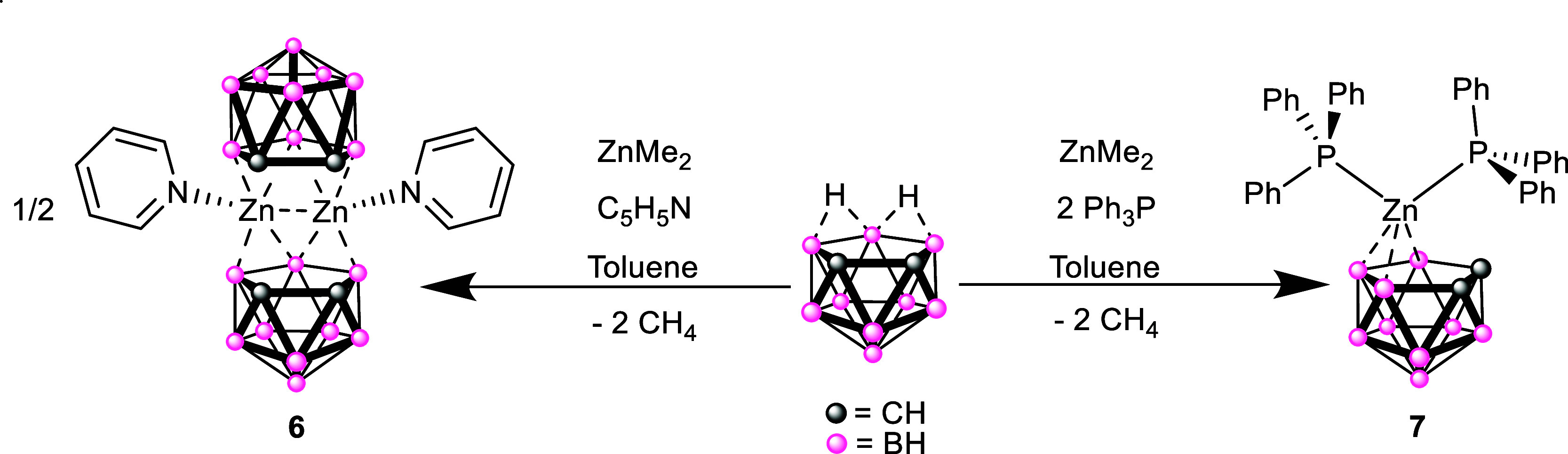
Synthesis of the Zincacarborane Complexes **6** and **7**

^1^H, ^13^C­{^1^H} and ^11^B NMR spectra of **6** in CD_2_Cl_2_, are consistent with the previously reported NMe_3_ complex (reported in C_5_D_5_N).^6a^ As with **1**–**3**, NMR spectra show clear absence of resonances associated with either free ZnMe_2_ or Py-ZnMe_2_ adducts^20,33^ and ^1^H NMR spectra were consistent with a product with a 1:1 C_5_H_5_N:{C_2_B_9_H_11_} ratio. While the stoichiometry of the product was identical to the expected monomeric *closo*-icosahedral metallacarborane [C_5_H_5_N·Zn­{C_2_B_9_H_11_}], X-ray crystallographic studies show the product, **6**, to have an analogous structure to the previously reported macropolyhedral dimeric structure, *commo*-[(μ^2^-C_2_B_9_H_11_)­Zn·NMe_3_]_2_ (**VIII**, Figure 3) in which the NMe_3_ groups have been replaced by pyridine groups (Figure 7).

**Figure 7:**
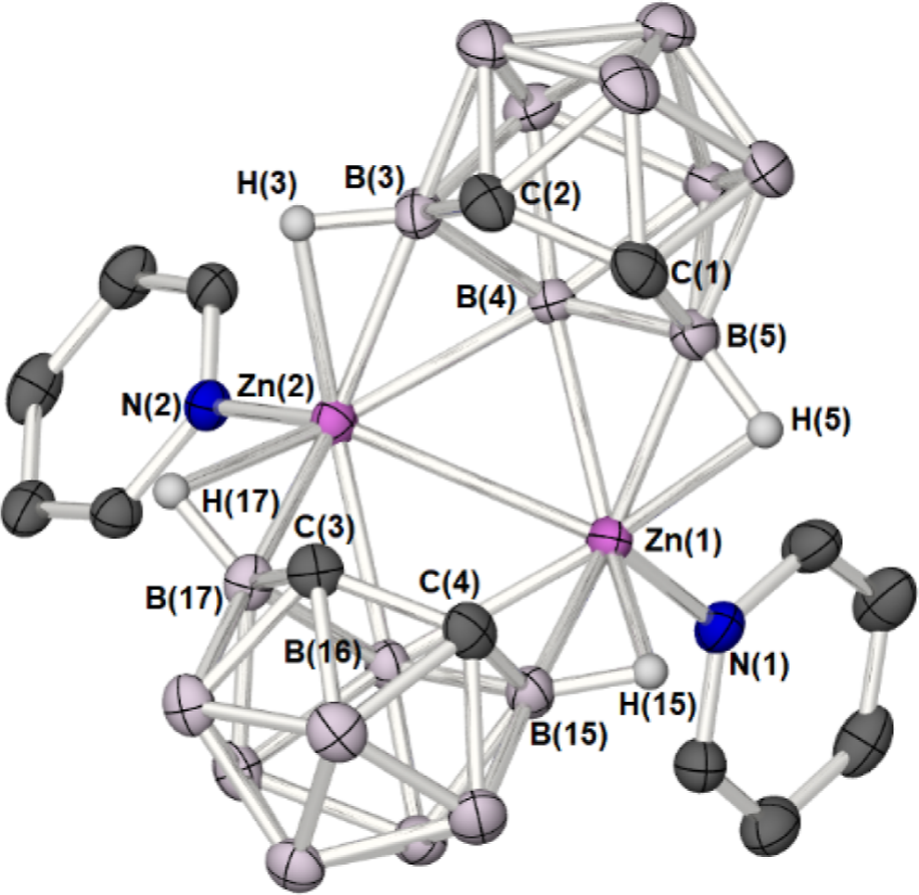
Molecular structure of the macropolyhedral zincacarborane *commo*-[(μ^2^-C_2_B_9_H_11_)­Zn·NC_5_H_5_]_2_ (**6**) (50% probability ellipsoids). Hydrogen atoms, and solvent of crystallization (Toluene) have been omitted for clarity.

Despite the empirical/formulaic similarities between the *closo*-[NHC–Zn­{C_2_B_9_H_11_}] systems (**1–3**) and the macropolyhedral clusters *commo*-[(μ^2^-C_2_B_9_H_11_)­Zn·L]_2_ [L = Pyridine (**6**) or NMe_3_] the precise reasons for the very clear difference in the solid-state structures is unclear: pyridine and NMe_3_ are both essentially pure 2-electron σ-donor ligands. NHC ligands, also 2-electron donors, are considered to be stronger σ-donors, whereas NHC···M interactions are generally considered to be more covalent and also have the possibility of π-back bonding interactions under certain conditions. Table 2 shows selected bond lengths and bond angles for **6**.

**Table 2:** Selected Atomic Distances (Å) and Bond Angles (°) for Complex **6**

bond lengths (Å)			
Zn(1)–Zn(2)	2.7660(3)	C(1)–C(2)/C(3)–C(4)	1.533(3)/1.531(3)
Zn(1)–B(4)/Zn(1)–B(16)	2.328(2)/2.365(2)	Zn(2)–B(4)/Zn(2)–B(16)	2.340(2)/2.327(2)
Zn(1)–B(5)/Zn(1)–B(15)	2.157(2)/2.142(2)	Zn(2)–B(3)/Zn(2)–B(17)	2.156(2)/2.154(2)
Zn(1)–H(5)/Zn(1)–H(15)	1.983(2)/1.920(2)	Zn(2)–H(3)/Zn(2)–H(17)	1.991(2)/2.021(2)
Zn(1)–N(1)	2.0191(17)	Zn(2)–N(2)	2.0259(15)
{C_2_B_3_}_pln_···{C_2_B_3_}_pln_	3.7839(16)		
bond angles (°)			
Zn(1)–Zn(2)–N(2)	125.87(5)	Zn(2)–Zn(1)–N(1)	126.94(5)
Zn(1)–B(4)–Zn(2)	72.67(6)	Zn(1)–B(16)–Zn(2)	72.24(6)
B(4)–Zn(1)–B(16)	106.91(7)	B(4)–Zn(2)–B(16)	107.78(7)
B(5)–Zn(1)–B(15)	119.87(9)	B(3)–Zn(2)–B(17)	125.40(8)

As with *commo*-[(μ^2^-C_2_B_9_H_11_)­Zn·NMe_3_]_2_, **VIII**, the molecular structure of the pyridine adduct **6** possesses approximate *C*_*2v*_ molecular symmetry with no crystallographically imposed symmetry. Similarly to *commo*-[(μ^2^-C_2_B_9_H_11_)­Zn·NMe_3_]_2_, two {*nido*-C_2_B_9_H_11_} fragments are connected/bridge through the unique boron atoms, on the open face of the *nido*-carborane to a {Py·Zn–Zn·Py} unit with a highly unusual planar, diamond-shaped {Zn_2_B_2_} motif, in which the two zinc atoms are at a separation of 2.7660(2) Å, marginally longer than the similar bond in **VIII** (cf. 2.665 Å) and approaching a comparable distance to Zn···Zn interaction in zinc metal. [i.e. 2.61–2.87(1) Å].^34^

The two zinc atoms at the core of the structure are both 8-coordinate with connectivity to the two unique hepta-coordinate boron vertices in the open face of the {*nido*-C_2_B_9_H_11_} fragments, two hexa-coordinate boron vertices in the open face of the {*nido*-C_2_B_9_H_11_} fragments, two B–H groups and the nitrogen atom of the pyridine ligand.

As previously noted,^6a^ the planar diamond-shaped {Zn_2_B_2_} ring system in **6** is reminiscent of the 3-center 2-electron (3c2e) bonds observed in species such as ArZn­(μ-H)_2_ZnAr (Ar = C_6_H_3_-2,6-C_6_H_3_-2,6-*i*Pr_*2*_)_2_ or 4-SiMe_3_-C_6_H_3_-2,6-(C_6_H_3_-2,6-*i*Pr_*2*_)_2_)^35^ and Zn_2_Ph_4_.^36^ While similarities exist between these systems and **6**, there is no evidence to support the presence of 3c2e {Zn–B–Zn} bonding. In addition to the {Zn_2_B_2_} ring, X-ray analysis would suggest coordination about the zinc atom is completed by one pyridine (NC_5_H_5_) ligand and interaction of a pair of {B–H···Zn} interactions with each zinc atom.

Addition of one equivalent of triphenylphosphine to a toluene solution of C_2_B_9_H_13_ showed no obvious reaction. Subsequent addition of one equivalent of ZnMe_2_, at −78 °C, results in the formation of a cloudy solution on warming to room temperature. Filtration and storage at −28 °C of the solution resulted in the formation of colorless crystals of **7** (Scheme 2). ^1^H NMR spectroscopy of the product, clearly showed the presence of Ph_3_P and {C_2_B_9_H_11_} groups in a 2:1 ratio. However, ^31^P­{^1^H} NMR spectroscopy displayed only a single resonance at −5.1 ppm. As with **1**–**6**, the absence of resonances associated with ZnMe_2_ groups was consistent with complete reaction of C_2_B_9_H_13_ with ZnMe_2_ and formation of a system with a [(Ph_3_P)_2_Zn­{C_2_B_9_H_11_}] formulation. Addition of C_2_B_9_H_13_ to a 1:1 mixture of triphenylphosphine and ZnMe_2_, also yielded **7**.

Indeed, an X-ray crystallographic study shows the product, **7**, to be a *bis*-phosphine zincacarborane system, [(*nido*-C_2_B_9_H_11_)­Zn·(PPh_3_)_2_] (Figure 8), in which the V-shaped {Zn­(PPh_3_)_2_} fragment has undergone a significant slippage of the Zn away from the two C atoms and toward the three B atoms. Table 3 shows selected bond lengths and angles for **7**. The orientation of the {Zn­(PPh_3_)_2_} fragment is such that one phosphine lies over the {C_2_B_3_} face of the dicarbollide ligand (*endo*) with a Zn–P bond length of 2.3821(4) Å, while the second phosphine group is orientated in an *exo*-fashion with a longer Zn–P bond length of 2.5270(4) Å.

**Figure 8:**
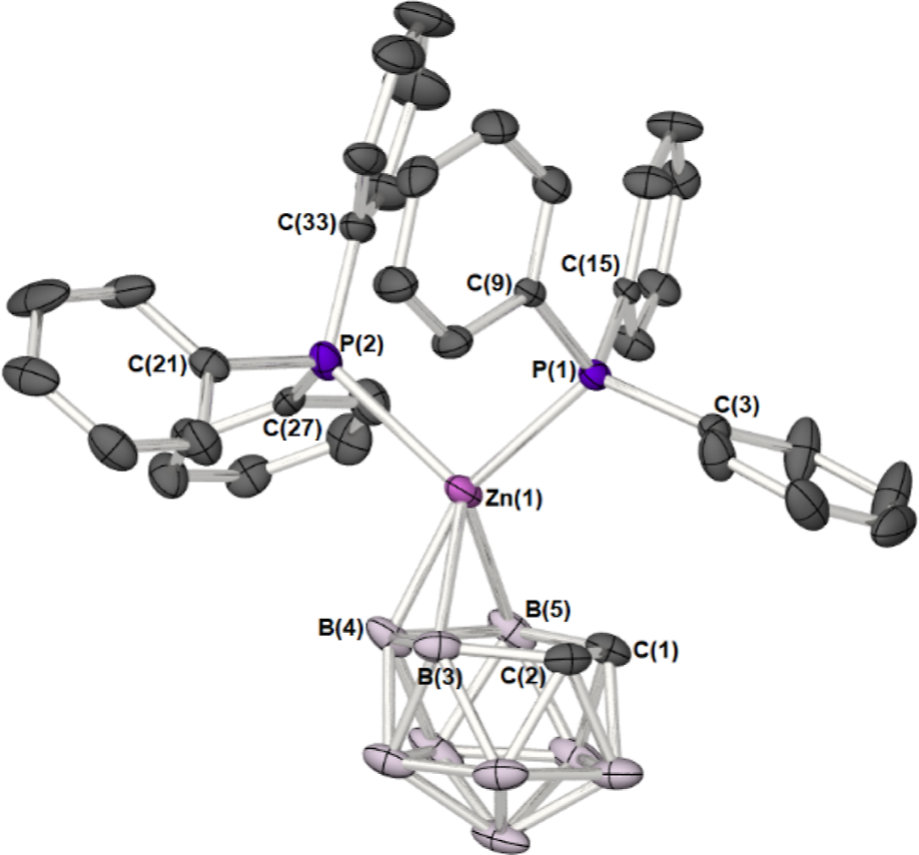
Molecular structure of the zincacarborane [(Ph_3_P)_2_Zn­(η^3^-C_2_B_9_H_11_)] (**7**) (50% probability ellipsoids). Hydrogen atoms have been omitted for clarity.

**Table 3:** Selected Bond Lengths (Å) and Angles (°) for Complex **7** a

bond lengths	
Zn(1)–C(1)	2.714(2)
Zn(1)–C(2)	2.747(2)
Zn(1)–B(3)	2.357(2)
Zn(1)–B(4)	2.100 (2)
Zn(1)–B(5)	2.303(2)
Zn(1)–P(1)_endo_	2.3821(4)
Zn(1)–P(2)_exo_	2.5270(4)
Zn(1)–{C_2_B_3_}_cent_	1.9914(2)
C(1)–C(2)	1.544(2)
Δ/h	0.76/3.375
bond angles	
P(1)–Zn(1)–P(2)	101.542(14)
P(1)–Zn(1)–{C_2_B_3_}_cent_–{C_2_}_cent_	6.0235(3)
P(2)–Zn(1)–{C_2_B_3_}_cent_–{C_2_}_cent_	169.5070(6)
P(1)–Zn(1)–B(4)	157.01(5)
P(2)–Zn(1)–B(4)	101.22(5)

a As noted above, the most significant structural feature of **7** is that the Zn atom is displaced from the center of the {C_2_B_3_} face by 0.76 Å (Δ). The Zn–C distances [Zn(1)­C(1) = 2.714(2) Å; Zn(1)–C(2) = 2.747(2) Å] exceed the sum of covalent radii for Zn and C (ca. 1.98 Å); hence the bonding between the Zn atom and the dicarbollyl ligand is exclusively to boron atoms B(3), B(4), and B(5) [2.100(2)–2.357(2) Å]. As such, **5** can be regarded as η^3^-borallyl complexes.

**7** crystallizes in the triclinic space group *P-*1, with one molecule per asymmetric unit, with disorder in the positions of several carbons of the phenyl groups in the PPh_3_ ligand (C9/9A; C15/15A; C27/27A; C33/33A).

Figure 9 shows the relative projections of the {ML_2_} moieties into the plane of the {C_2_B_3_} face of the [C_2_B_9_H_11_] ligand. In the case of **7**, the {ZnP_2_} plane of the d^10^-{Zn­(PPh_3_)_2_}^2+^ fragment lies almost parallel, *syn*-periplanar, to the molecular mirror plane which passes through B(4) and the midpoint of the C­(l)–C(2) bond such that the torsion angle, P(1)–Zn(1)–{C_2_B_3_}_cent_–{C_2_}_cent_, is 6.0235(3)°. This is in stark contrast to related systems such as [(dppe)_2_Ni­(C_2_B_9_H_11_)],^37^ [(dppe)­Pt­(C_2_B_9_H_11_)],^38^ [(TMEDA)­Pd­(C_2_B_9_H_11_)][Bibr cit11a] [(Me_3_P)_2_Pd­(C_2_B_9_H_11_)],^11a^ [(Et_3_P)_2_Pt­(C_2_B_9_H_11_)],^7a^ [(Ph_3_P)_2_Pt­(PhMeC_2_B_9_H_9_)][Bibr cit11c] and [(Et_3_P)_2_Pt­(PhMeC_2_B_9_H_9_)],^11c^ in which the d^8^-{ML_2_}^2+^ fragments’ {ML_2_} planes are orientated perpendicular to the mirror plane which runs through the carborane face, bonding to the {C_2_B_3_} face in a η^5^-fashion (Δ = 0–0.4).^7a^ In the case of the latter d^8^-systems the relative orientation of the {ML_2_} fragments has been rationalized on the basis of specific molecular orbital interactions between the dicarbollyl ligand and d^8^-ML_2_ fragments.^7a^

**Figure 9:**
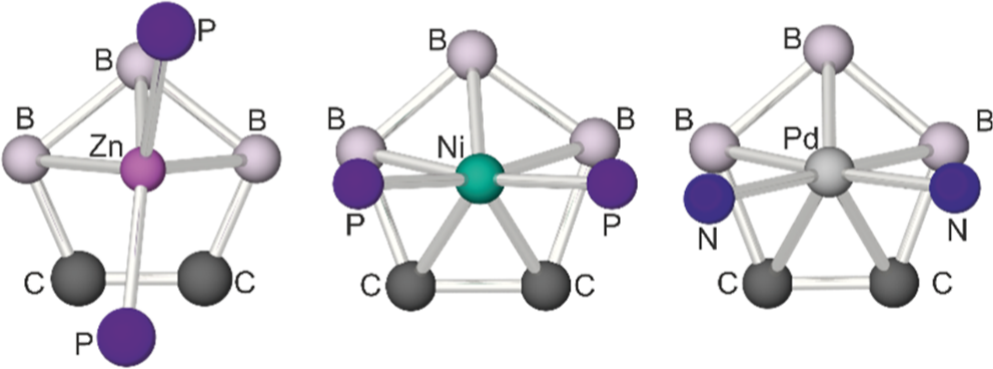
Projection of the {ML_2_} group in **7**, [(dppe)_2_Ni­(C_2_B_9_H_11_)][Bibr ref37] and [(TMEDA)­Pd­(C_2_B_9_H_11_)][Bibr cit11a] onto the atoms on to the plane defined by {C_2_B_3_} face of the [C_2_B_9_H_11_] ligand, showing the slipping and relative orientation of the {ML_2_}, fragment.

### QTAIM: A Topological Analysis

To better understand the details of the electronic structure of the metal–dicarbollide complexes, topological analysis of the electron density was conducted. This analysis employed the quantum theory of atoms in molecules (QTAIM),^39,40^ which has previously been shown to be of great use in understanding covalency trends and various properties of molecules, including bond strengths, charge distributions, and the nature of intermolecular interactions.

What is most noteworthy here are the bond critical points (BCPs) located between atom pairs. These BCPs occur where the bond path, defined as the path of maximum electron density between two atoms, intersects with the interatomic surfaces. However, it should be noted that the absence of a bond path between two atoms does not necessarily mean there is no bonding interaction.^41^ The value of the electron density at this point, along with its Laplacian, are used to interpret the nature of the interactions. Generally, ρ_BCP_ > 0.20 a_0_ and ∇^2^ρ_BCP_ < 0 for a covalent bond, while ρ_BCP_ < 0.10 a_0_ and ∇^2^ρ_BCP_ > 0 indicates an ionic bond. More broadly speaking, increasing values of ρ_BCP_ and reduction in ∇^2^ρ_BCP_ indicate increasing covalent character and stronger bonding interactions. In addition, the ratio of the magnitude of the potential energy density (|*V*|) to the kinetic energy density (*G*) at a bond critical point, is a crucial indicator of the type of bonding interaction;^42^ where |*V*/*G*| < 1 noncovalent interactions predominate, i.e. ionic or weak van der Waals interactions. Conversely, if the magnitude of the potential energy is greater than the kinetic energy, i.e. |*V*/*G*| > 1, interactions are categorized as shared-shell interaction, or covalent bonds.

QTAIM analysis has been previously applied selectively to borane,^43^ metalloboranes^44^ and carborane systems^45^ as well as extensively to organometallic complexes^46^ and clusters.^47^ However, to the best of our knowledge it has, to date, been applied to only one metallacarborane system.^4c^

The geometries of complexes **1**–**3**, **5–7** and **VIII** were optimized using density functional theory (DFT) based on crystallographic structures using the BP86/BS1 level of theory (see the Supporting Information for the full methodology). Computed structures are fully consistent with the crystallographically determined structures. Table 4: lists selected topological properties, ρ, ∇^2^ρ, and |*V*/*G*| for selected bonds in the computed complexes.

**Table 4:** Topological Properties, ρ, ∇^2^ρ, and |*V*/*G*| for Selected for Selected Bonds in Complexes **1**–**3**, **5**–**7** and **VIII** (BP86/BS2//BP86/BS1)

complex	interaction	ρ_BCP_	∇^2^ρ_BCP_	|*V*_BCP_/*G*_BCP_|	ref
**1**	Zn–C	0.1084	0.2343	1.427	this work
	Zn–B_(Cb)_	0.0832	0.0349	1.797	
**2**	Zn–C	0.1066	0.2229	1.433	this work
	Zn–B_(Cb)_	0.0825	0.0361	1.788	
	Zn···H_(iPr)_	0.0104	0.0257	0.887	
**3**	Zn–C	0.1073	0.2340	1.422	this work
	Zn–B_(Cb)_	0.0828	0.0369	1.785	
**5**	Zn–B_(Cb)_	0.0749	0.0483	1.697	this work
**6**	Zn···Zn	0.0301	0.0491	1.264	this work
	Zn–N_(Py)_	0.0734	0.2411	1.232	
	Zn···B(4/7)	0.0655	0.0635	1.572	
**VIII**	Zn···Zn	-	-	-	^6a^
	Zn–N_(NMe3)_	0.0657 and 0.0643	0.1924 and 0.1869	1.240 and 1.237	
	Zn···B(4/7)	0.0647	0.0615 and 0.0652	1.576 and 1.560	
**7**	Zn–B_(Cb)_	0.0789	0.044	1.74	this work
	Zn–PPh_3(endo)_	0.064	0.0759	1.52	
	Zn–PPh_3(exo)_	0.042	0.054	1.41	

Clear intramolecular bond paths associated with internal bonds within both the dicarbollide {C_2_B_9_H_11_} and NHC ligands (i.e., NHC = *a*, *b* and *c*, Scheme 1) respectively, are observed. In the case of complexes **1**–**3**, bond critical points between Zn and the NHC ligands were found, with values of ρ_BCP_ > ∼0.1 observed for the NHC-carbon zinc interaction. When coupled with positive Laplacian values (∇^2^ρ_BCP_ = ∼0.23), low electron density supports the idea of interactions with strong ionic character, and very little variation is seen in the values irrespective of the NHC.

Analysis reveals a bond critical point between the dicarbollide ligand [C_2_B_9_H_11_]^2–^, exclusively via the unique central B atom (B_(Cb)_) of the open face of the ligand, and [Zn^2+^]. Here, the electron density at the bond critical points is lower than that between the Zn atom and the NHC ligand, with ρ_BCP_ ≈ 0.08 per a_0_ indicating closed shell, strongly ionic bonding interactions between the [Zn^2+^] and [C_2_B_9_H_11_]^2–^. This stands in contrast to analysis performed on charge compensated silver–metallacarboranes, in which π-interactions between the Ag ions and the pentagonal {C_2_B_3_} open face have been shown to exist.^4c^ Similar, bonding motifs, have previously been observed in pentadienyl-zirconium complexes, where metal–ligand interactions are predominantly between the metal and the most electronegative carbon atom, i.e., the central C atom of the of the pentadienyl ligand.^46a^ A positive Laplacian (∇^2^ρ_BCP_ = ∼0.034) supports the idea of a weak ionic interaction comparable to NO···HN hydrogen bonding interactions in strength.^48^

Figure 10 shows a contour plot of the Laplacian (∇^2^ρ_BCP_) in the {C–Zn–B_(Cb)_} plane of the DFT-optimized structure of **2** with bond critical points (BCPs) highlighted. Consistent with this are the |*V*/*G*| values, which also serve as a key indicator of bond character. Typically, values 0.5 < |*V*/*G*| < 1 are indicative of closed-shell or ionic interactions. Values greater than 2, indicate a shared-shell interaction (covalent bond). Indeed, the |*V*/*G*| values are between 1.7 and 1.8, as for the Zn–B_(Cb)_ interactions theses are generally interpreted as indicating a covalent bond, specifically a polar covalent bond.

In the case of the Zn–NHC interactions the |*V*/*G*| values (∼1.4) suggest a significant sharing of electrons between the atoms involved, i.e. a higher degree of covalency, but with a notable degree of charge separation presumably due to electronegativity differences. Despite this, NBO analysis did not reveal bonding orbitals between the zinc atom and the dicarbollide, or the NHC ligands, presumably due to the significant electrostatic contributions to the bonding.

In the case of complex **2**, an additional noteworthy feature is the presence of a distinct H···Zn interaction of a methyl-group H atom, on a substituent of the NHC, with the Zn metal center. QTAIM analysis reveals the presence of a BCP indicative of a weak H-bonding interaction:^49^ a feature that is also noted in the single crystal X-ray diffraction studies of **2** (Figure 4b).

**Figure 10:**
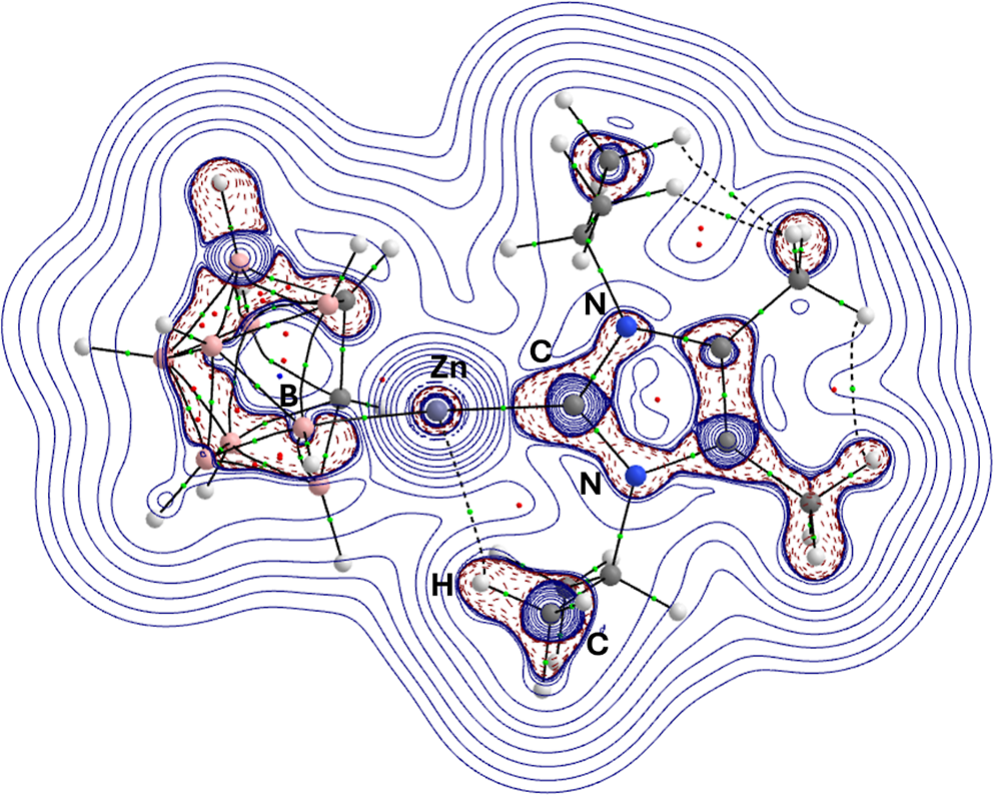
Contour plot of the Laplacian (∇^2^ρ_(r)_) in the {C–Zn–H} plane of the DFT-optimized 2 (BP86/BS2//BP86/BS1). Bond critical points (BCPs) are depicted as green spheres, ring critical points (RCPs) are depicted as dark red spheres.

For the zinc *bis*-dicarbollide system, **5**, QTAIM analysis shows clear bond critical points between the zinc atom and the unique boron atom of the {C_2_B_3_} face, B_(cb)_. Again, QTAIM values provide insight into the chemical bonding, with positive values for both ρ and ∇^2^ρ at the bond critical point (BCP) indicating a closed-shell ionic interaction (ρ = 0.0749/0.0749, ∇^2^ρ = 0.0483/0.0483 and |*V*/*G*| = 1.697/1.697) such that the zinc center may be considered linear 2-coordinate cf. ZnMe_2_. The low Laplacian value found for **5**, also indicates a stronger Zn–B_(Cb)_ interaction that those found for **1**–**3**. The |*V*/*G*| ratio also suggests a closed-shell interaction, not dissimilar to the Zn–B_(CB)_ interaction observed in **1–3**.

For complex **6** and **VIII**, both are dominated by the highly unusual planar, diamond-shaped {Zn_2_B_2_} bonding motif, previously credited to the presence of 3-center {B–Zn–B} interactions, between the unique atom on the open face of the dicarbollide ligand, B_(Cb)_, and the zinc atoms, with the possibility of additional {Zn^2+^···Zn^2+^} interactions.^6a^ QTAIM analysis reveals a much more “ionic” bonding solution. While clear intramolecular bond paths are found for both the dicarbollide and ancillary ligands i.e., C_5_H_5_N and NMe_3_ respectively, in the case of **6** values for ρ, ∇^2^ρ, and |*V*/*G*| for the Zn···Zn interaction characterize a weak ionic interaction (ρ = 0.0301; ∇^2^ρ = 0.0491; |*V*/*G*|: 1.264). While {Zn^2+^···Zn^2+^} interactions have not been previously observed, {Zn^+^···Zn^+^} are known and have been evaluated by QTAIM analysis. Not surprisingly, topological values for molecules such as [Zn_2_Cp_2_] (ρ = 0.065; ∇^2^ρ = 0.069; |*V*/*G*| = 1.53) and [Zn_2_Ph_2_] (ρ = 0.062; ∇^2^ρ = 0.019; |*V*/*G*| = 1.884), both with shorter Zn···Zn interactions (ca. 2.3–2.4 Å) cf. 2.7660(3) Å (**6**) and 2.800(1) Å (**VIII**)^46b^ are significantly different to those calculated for molecules such as **6**. Figure 11 shows a contour plot of the Laplacian (∇^2^ρ) in the {Zn–Zn–B} plane of the DFT-optimized **6**.

**Figure 11:**
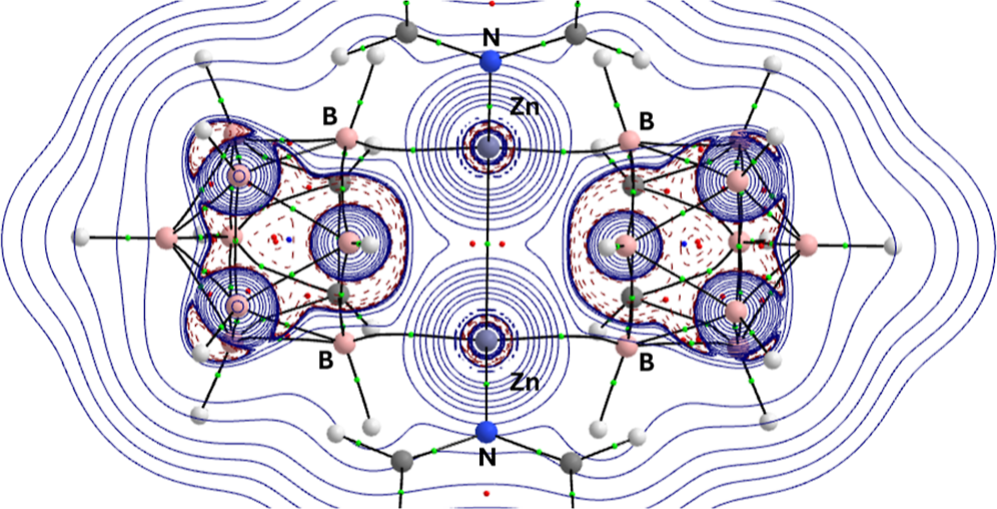
Contour plot of the Laplacian (∇^2^ρ­(r)) in the {Zn–Zn–B} plane of the DFT-optimized 6 (BP86/BS2//BP86/BS1) showing the Zn···Zn interaction and the dicarbollide cages. Bond critical points (BCPs) are depicted as green spheres, ring critical points (RCPs) are depicted as dark red spheres.

QTAIM analysis of **6**, as indicated by a |*V*/*G*| value of 1.264 reveals the [Zn···Zn] interaction, is significantly less covalent than the interactions in [Zn_2_Cp_2_] and [Zn_2_Ph_2_], as expected on the difference in Zn-oxidation states. In stark contrast the cluster (**VIII**) that was also investigated using QTAIM is revealed to have no discernible Zn···Zn interaction.

In both cases, unlike structures **1–3** and **5**, the interaction between the zinc atoms and the dicarbollide anions, is not through the unique B atom in the open face (B_(Cb)_), but rather through the B atoms adjacent to the C–C bond in the open face (B4 and B7). Here ρ_(BCP)_ values of ∼0.065 a_0_ are observed typically indicating an intermediate or partially covalent interaction between atoms. These values fall within a range where bonds are neither purely ionic nor purely covalent but exhibit characteristics of both. A |*V*/*G*| value of ∼1.56 in both systems indicates a predominantly covalent bonding character within the interactions.

Surprisingly, the interaction of the ancillary ligands, C_5_H_5_N and NMe_3_ with the zinc atoms in each cluster is also different. In the case of **6** (see Supporting Information), NBO finds the Zn–Py interactions are negligible and below the threshold of reporting. This is supported by QTAIM analysis, which finds electron density values of ρ = 0.0733 and Laplacian values of ∇^2^ρ = 0.241 for the Zn–N_(Py)_ bonds indicating a primarily ionic interaction.

These values are comparable to the Zn–N_(NMe3)_ interactions found in **VIII** showing slightly less electron density at the Zn–N BCPs.

We believe that it is important to note that while single crystal X-ray diffraction studies (SCXRD) indicate the potential for stabilizing Zn···H–B interactions in the case of **VIII**, DFT analysis reveal no such stabilizing interactions. However, in the case of **6**, weak (∼27.2 kcal mol^–1^) Zn···H–B interactions are observed, with donor/acceptor orbitals found in the NBO data.

For the *bis*-phosphine complex, **7**, SCXRD reveals a difference in bond length between the *endo*- and *exo*-PPh_3_ groups. This is consistent with NBO data where the two Zn-phosphine interactions are different: Wiberg bond indices (WBI) for Zn–P are 0.328 (*endo*) and 0.207 (*exo*) indicative of less shared electron density along the Zn–P_(*exo*)_ bond in comparison to the Zn–P_(*endo*)_ bond. ρ, ∇^2^ρ, and |*V*/*G*| values for the two bonds are also different (Table 4) with |*V*/*G*| values indicating a more covalent interaction between the Zn atom and the *endo*-PPh_3_ group compared to the *exo*-PPh_3_ group.

As with complexes **1–3**, bonding between the Zn atom and the dicarbollide is exclusively between the [Zn^2+^] and unique central B atom (B_(Cb)_) of the open face of the ligand. |*V*/*G*| values (1.74) are comparable with other systems in this series and suggest a polar covalent interaction between the metal and B_(Cb)_. In all cases WBI values all show predominantly weak interactions between the dicarbollide ligand, NHC and phosphine ligands and the zinc metal atom.

## Conclusions

Metallocarborane chemistry, despite being about 60 years old, still has secrets to reveal.^3a^ In an attempt to expand the number of known zincacarborane systems, we have prepared a family of NHC adduct systems (**1**–**3**). In the case of the NHC ligands (Scheme 1) we have been able to synthesize, by the reaction of [Me_2_Zn·NHC] with the acidic carborane C_2_B_9_H_13_, and structurally characterize, zincacarborane systems of the general form [(C_2_B_9_H_9_)­Zn­{NHC}]; where, as shown by SCXRD, the {Zn­(NHC)} fragment sits relatively symmetrically over the face of the dicarbollide ligand.

In the case of more sterically demanding alkyl-NHCs, i.e. with ^*t*^Bu and adamantyl substituents at the nitrogen atoms, reaction between [Me_2_Zn·NHC] and C_2_B_9_H_13_ yield the zincacarborane salts [NHC–H]_2_[(C_2_B_9_H_11_)_2_Zn], **4**–**5**, with the adamantyl derivative (**5**) having been structurally characterized by SCXRD.

Reaction of ZnMe_2_ with C_2_B_9_H_13_ in the presence of either one equivalent of pyridine or triphenylphosphine yields the zincacarborane complexes [μ^2^-(C_2_B_9_H_11_)­Zn·Py]_2_ (**6**) and [(C_2_B_9_H_11_)­Zn­(PPh_3_)_2_]­(**7**) respectively. In the case of **6** the structure of the complex is analogous to that of the trimethylamine zincacarborane adduct, **VIII**, described by Wade and Hughes^6a^ that possesses a planar diamond-shaped {Zn_2_B_2_} motif at its center. In the case of the phosphine adduct, **7**, a zincacarborane system with a V-shaped {Zn­(PPh_3_)_2_} moiety appended to the dicarbollide ligand is revealed.

X-ray diffraction primarily reveals the average positions of atoms, and from bond lengths and bond angles, the nature of interactions may be inferred. In contrast DFT calculations, and in this instance primarily QTAIM analysis, provide a quantum mechanical description of bonding, including electron density and orbital interactions. These differences can lead to varied interpretations of bonding characteristics. For the first time we have used QTAIM to analyze the bonding interaction within this series of zincacarborane systems.

Most revealing has been the nature of the dicarbollide ligand with the [Zn^2+^] ion, which reveal low values of the electronic density (ρ) and the positive values of the Laplacian (∇^2^ρ), which, according to Bianchi’s classification, are considered closed shell and ionic in nature.^50^ Other parameters resulting from the QTAIM calculations indicate that the Zn–NHC interaction can similarly be classified as highly ionic (Table 4). However, on closer inspection, the values of |*V*_BCP_/*G*_BCP_| at the BCPs between the zinc ion and the other coordinated ligands (dicarbollide, NHC, amine and phosphine) are intermediate between ionic and covalent character, since 1 < |*V*/*G*| < 2.

What can be clearly seen is that single crystal X-ray diffraction studies (SCXRD) are not necessarily a direct indicator of the presence, or nature of bonding interactions. In fact, here a reliance on SCXRD studies would have suggested significantly greater interactions between the dicarbollide ligand and zinc atoms, or the tantalizing suggestion of {Zn···Zn} interactions in both **6** and **VIII**, than that indicated by computational analysis.

## Experimental Section

Complexes **1–5** were synthesized under ambient conditions using reagent grade solvents that had not been subject to further purification. All manipulations of air- and moisture-sensitive compounds were carried out under an atmosphere of nitrogen or argon using standard Schlenk-line or glovebox techniques. Solvents were dried according to standard methods and collected by distillation. ZnMe_2_ was purchased from commercial sources and used without further purification. C_2_B_9_H_13_ ^51^ and NHC-ligand **a–e** ^52^ where synthesized according to literature procedures.

^1^H, ^11^B and ^13^C­{^1^H} NMR spectra were recorded on Bruker Avance 400 or 500 MHz FT-NMR spectrometers, in saturated solutions at 298 K. Chemical shifts are expressed in ppm with respect to residual protic solvent (^1^H and ^13^C), BF_3_·OEt_2_ (^11^B), or Me_4_Sn (^119^Sn). Elemental analyses were performed at Elemental Microanalysis Ltd., Okehampton, Devon, UK All samples were run in duplicate. Solvents were dried by passage through a commercially available solvent purification system and stored under argon in ampules over 4 Å molecular sieves. C_6_D_6_ was purchased from Merck, dried over potassium before distilling and storage over 4 Å molecular sieves. No uncommon hazards are noted.

### Preparation of [IMe_4_·Zn­{η^5^-C_2_B_9_H_11_}] (**1**)

A 1.2 M solution of ZnMe_2_ in toluene (0.83 mL, 1 mmol) was added to a toluene (15 mL) solution of IMe_4_ (0.13 g, 1 mmol). After stirring for 4 h a solution of C_2_B_9_H_13_ (0.13 g, 1 mmol) in toluene (5 mL) was added at 0 °C to afford an insoluble cream-colored precipitate. The reaction mixture was dried under vacuum and DCM (30 mL) added. The yellow solution was then filtered and concentrated to 10 mL, after which colorless crystals of **1** were afforded at −28 °C. (0.28 g, 87%); ^1^H NMR (500 MHz, CD_2_Cl_2_): δ 3.93 (6H, s, NMe), 2.26 (6H, s, C­(4,5)-Me), 2.03 (2H, br s, carborane C–H); ^13^C­{^1^H} NMR (125.8 MHz): δ 160.3 (NC:N), 128.9 (C­(4,5)), 42.3 (carborane C–H), 36.5 (NMe), 9.4 (C­(4,5)-Me); ^11^B NMR (160.5 MHz): δ −14.6 (2B, d, *J* = 133 Hz), −19.1 (1B, d, *J* = 158 Hz), −21.6 (3B, d, *J* = 133 Hz), −23.1 (2B, s), −32.1 (1B, d, *J* = 144 Hz); ^11^B­{^1^H} NMR (160.5 MHz): δ −14.6 (2B, s), −19.1 (1B, s), −21.5 (3B, s), −22.9 (2B, s), −32.2 (1B, s); Anal. Calc. for C_9_H_23_B_9_N_2_Zn_1_: C, 33.57; H, 7.20; N, 8.70. Found: C, 34.01; H, 7.15; N, 8.74.

### Preparation of [I^*i*^Pr_2_Me_2_·Zn­{η^5^-C_2_B_9_H_11_}] (**2**)

A 1.2 M solution of ZnMe_2_ in toluene (1.25 mL, 1.5 mmol) was added to a toluene (20 mL) solution of I^*i*^Pr_2_Me_2_ (0.27 g, 1.5 mmol). After stirring for 18 h volatiles were removed in vacuo to yield a white solid. The solid was dissolved in toluene (25 mL) and added to a solution of C_2_B_9_H_13_ (0.2 g, 1.5 mmol) in toluene (10 mL) at 0 °C to afford a pale-yellow solution with an insoluble white precipitate. The reaction mixture was dried under vacuum and DCM (40 mL) added. The pale-yellow solution was then filtered and concentrated to 20 mL. after which colorless crystals of **2** were afforded at −28 °C. (0.45 g, 80%); ^1^H NMR (300 MHz, CD_2_Cl_2_): δ 4.98 (2H, hept, *J* = 7.0 Hz, CHMe_2_), 2.30 (6H, s, C­(4,5)-Me), 1.99 (2H, br s, carborane C–H), 1.68 (12H, d, *J* = 7.0 Hz, CHMe_2_); ^13^C­{^1^H} NMR (75.5 MHz): δ 157.9 (NC:N), 128.6 (C­(4,5)), 54.6 (CHMe_2_), 42.2 (carborane C–H), 23.3 (CHMe_2_), 10.5 (C­(4,5)-Me); ^11^B NMR (160 MHz): δ −14.4 (2B, d, *J* = 138 Hz), −18.7 (1B, d, *J* = 167 Hz), −21.2 (4B, d, *J* = 136 Hz), −23.1 (1B, d, *J* = 125 Hz), −32.8 (1B, d, *J* = 143 Hz); ^11^B­{^1^H} NMR (160 MHz) δ −14.4 (2B, s), −18.6 (1B, s), −21.2 (4B, s), −23.5 (1B, s), −32.8 (1B, s); Anal. Calc. for C_13_H_31_B_9_N_2_Zn_1_: C, 41.30; H, 8.27; N, 7.41. Found: C, 41.10; H, 7.68; N, 8.72.

### Preparation of [IPr·Zn­{η^5^-C_2_B_9_H_11_}] (**3**)

A 1.2 M solution of ZnMe_2_ in toluene (0.83 mL, 1 mmol) was added to a toluene solution of IPr (0.39 g, 1 mmol) at −78 °C and left to stir for 18 h. The pale orange solution was filtered and added dropwise to a toluene solution (7 mL) of C_2_B_9_H_13_ (0.13 g, 1 mmol); precipitate was observed on addition. The reaction mixture was dried to a pale-yellow solid which was recrystallized in DCM to afford colorless crystals of **3** at RT. (0.48 g, 82%) ^1^H NMR (300 MHz, CD_2_Cl_2_): δ 7.61 (2H, t, *J* = 7.8 Hz, p-C_6_H_3_), 7.57 (2H, s, C­(4,5)­H), 7.41 (4H, d, *J* = 7.8 Hz, m-C_6_H_3_), 2.59 (4H, hept, *J* = 6.9 Hz, CHMe_2_), 1.33 (12H, d, *J* = 6.9 Hz, CHMe_2_), 1.24 (12H, d, *J* = 6.8 Hz, CHMe_2_), 1.09 (2H, br s, carborane C–H); ^13^C­{^1^H} NMR (101 MHz): δ 168.4 (NC:N), 146.2 (ipso-C_6_H_3_), 133.3 (o-C_6_H_3_), 132.6 (p-C_6_H_3_), 126.6 (C­(4,5)), 125.4 (m-C_6_H_3_), 41.0 (carborane C–H), 29.8 (CHMe_2_), 25.5 (CHMe_2_), 23.6 (CHMe_2_); ^11^B NMR (160 MHz): δ −14.97 (2B, d, *J* = 134 Hz), −19.61 (1B, d, *J* = 162 Hz), −22.15 (4B, d, *J* = 131 Hz), −23.13 (1B, d, *J* = 126 Hz), −32.40 (1B, d, *J* = 142 Hz); ^11^B­{^1^H} NMR (160 MHz): δ −15.01 (2B), −19.56 (1B), −22.23 (4B), −23.08 (2B), −32.40 (1B); Anal. Calc. for C_29_H_47_B_9_N_2_Zn_1_: C, 59.40; H, 8.08; N, 4.78. Found: C, 58.8; H, 8.06; N, 4.42.

### Preparation of [I^*t*^Bu_2_]­[Zn­{η^3^-C_2_B_9_H_11_}_2_] (**4**)

A J. Young’s NMR tube was charged with I^*t*^Bu_2_ (10 mg, 55 μmol) and 0.5 mL of *d*_6_-benzene followed by a 2 M solution of ZnMe_2_ in toluene (27.5 μL, 55 μmol). After gentle agitation at room temperature for 30 min, 8 mg (55 μmol) of C_2_B_9_H_13_ in 0.5 mL of *d*_6_-benzene was added resulting in the precipitation of an insoluble white precipitate. The solvent was removed in vacuo to afford a white solid which was dissolved in *d*_2_-DCM (0.6 mL). ^1^H NMR (300 MHz, CD_2_Cl_2_): δ 8.23 (1H, s, C(2)-H), 7.51 (2H, d, *J* = 1.8 Hz, C­(4,5)-H), 1.70 (18H, s, CMe_3_); 1.15 (2H, br s, carborane C–H), ^13^C­{^1^H} NMR (75.5 MHz): δ 129.8 (NC­(H)­N), 121.3 (C­(4,5)), 61.5 (CMe_3_), 43.1 (carborane C–H), 30.2 (CMe_3_); ^11^B NMR (96.3 MHz): δ −8.1 (2B, d, *J* = 135 Hz), −14.1–17.8 (1B, m), −19.1 (2B, d, *J* = 147 Hz), −30.3 (1B, m), −35.0 (1B, d, *J* = 140 Hz); ^11^B­{^1^H} NMR (96.3 MHz): δ −8.1 (2B, s), −14.1 (2B, s), −17.8 (1B, s) −19.1 (2B, s), −30.3 (1B, s), −35.0 (1B, s); Anal. Calc. for C_26_H_64_B_18_N_4_Zn_1_: C, 45.08; H, 9.31; N, 8.09. Found: C, 45.14; H, 9.36; N, 7.02.

### Preparation of [IAd_2_]­[Zn­{η^3^-C_2_B_9_H_11_}_2_] (**5**)

A J. Young’s NMR tube was charged with IAd_2_ (19 mg, 55 μmol) and 0.5 mL of *d*_6_-benzene followed by a 2 M solution of ZnMe_2_ in toluene (27.5 μL, 55 μmol). After gentle agitation at room temperature for 30 min, 8 mg (55 μmol) of C_2_B_9_H_13_ in 0.5 mL of *d*_6_-benzene was added resulting in the precipitation of an insoluble white precipitate. The solvent was removed in vacuo to afford a white solid which was dissolved in *d*_2_-DCM (0.6 mL). Slow evaporation of the NMR solvent inside an inert atmosphere glovebox yielded colorless crystals of **5** suitable for single X-ray diffraction studies. ^1^H NMR (300 MHz, CD_2_Cl_2_): δ 8.28 (2H, t, *J* = 1.8 Hz, C(2)-H), 7.52 (4H, d, *J* = 1.8 Hz, C­(4,5)-H), 2.33 (12H, s, AdH­(5,6,7)), 2.16 (24H, dd, *J* = 18.4 Hz, 3.0 Hz, AdH­(2,3,4)), 1.81 (30H, m, AdH­(8,9,10) and carborane C–H/B–H); ^13^C­{^1^H} NMR (75.5 MHz, *d*_8_-THF): δ 131.1 (NC­(H)­N), 120.7 (C­(4,5)), 61.3 (AdC(1)), 44.0 (carborane C–H), 43.2 (AdC­(2,3,4)), 36.4 (AdC­(8,9,10)), 30.9 (AdC­(5,6,7)); ^11^B NMR (96.3 MHz, CD_2_Cl_2_): δ −8.6 (4B, d, *J* = 133 Hz), −14.4 (6B, m), −18.7 (4B, d, *J* = 149 Hz), −29.8 (2B, dd, *J* = 132 Hz, 54 Hz), −34.7 (2B, d, *J* = 140 Hz); ^11^B­{^1^H} NMR (96.3 MHz): δ −8.5 (4B, s), −13.8 (4B, s), −14.4 (2B, s), −18.7 (4B, s), −29.9 (2B, s), −34.8 (2B, s); Anal. Calc. for C_50_H_88_B_18_N_4_Zn_1_: C, 59.74; H, 8.82; N, 5.57. Found: C, 59.14; H, 8.74; N, 5.46.

### Preparation of [C_5_H_5_N·Zn­{μ^2^-C_2_B_9_H_11_}]_2_ (**6**)

Pyridine (0.08 mL, 1 mmol) was added to a stirring solution of C_2_B_9_H_13_ (0.134 g, 1 mmol) in toluene (12 mL) resulting in immediate formation of a white precipitate. A 1.2 M toluene solution of ZnMe_2_ (0.83 mL, 1 mmol) was then added dropwise to the suspension affording a clear and colorless solution. Colorless crystals of **6** were obtained at −28 °C. (0.39 g, 68%) ^1^H NMR (300 MHz, CD_2_Cl_2_): δ 8.46 (2H, d, *J* = 5.0 Hz, o-C_5_H_5_N), 8.06 (1H, t, *J* = 7.7 Hz, p-C_5_H_5_N), 7.63 (2H, t, *J* = 7.0 Hz, m-C_5_H_5_N), 2.23 (2H, br s, dicarbollide C–H); ^13^C­{^1^H} NMR (125.8 MHz): δ 148.9 (o-C_5_H_5_N), 142.2 (p-C_5_H_5_N), 126.9 (m-C_5_H_5_N), 47.2 (dicarbollide C–H), ^11^B NMR (160.5 MHz): δ −10.4 (4B, d, *J* = 139 Hz), −13.0 (4B, d, *J* = 139 Hz), −15.7 (2B, d, *J* = 155 Hz), −25.5 (4B, d, *J* = 95 Hz), −29.4 (2B, d, *J* = 93 Hz), −37.4 (2B, d, *J* = 142 Hz); ^11^B­{^1^H} NMR (160.5 MHz): δ −10.4 (4B, s), −13.0 (4B, s), −15.7 (2B, s), −25.5 (4B, s), −29.4 (2B, s), −37.4 (2B, s); Anal. Calc. for C_14_H_32_B_18_N_2_Zn_2_: C, 30.37; H, 5.86; N, 5.06. Found: C, 30.92; H, 5.90; N, 5.57.

### Preparation of [(Ph_3_P)_2_Zn­{η^3^-C_2_B_9_H_11_}] (**7**)

A 1.2 M solution of ZnMe_2_ in toluene (0.83 mL, 1 mmol) was added to a toluene solution of triphenylphosphine (0.26 g, 1 mmol) at room temperature and left to stir for 3 h. The clear solution was added dropwise, via canula, to a toluene solution (7 mL) of C_2_B_9_H_13_ (0.13 g, 1 mmol). The reaction mixture was filtered, the volume reduced in vacuo and stored at −28 °C yielding colorless crystals of **7** (0.29 g, 40%). ^1^H NMR (400 MHz, C_6_D_6_): δ 7.34 (12H, br s, *o*-C_6_H_5_), 7.01 (18H, br s, *m*- and *p*-C_6_H_6_), 1.91 (2H, s, carborane C–H); ^13^C NMR (101 MHz, C_6_D_6_), δ = 133.9 ppm (d, ^3^J­(^31^P,^13^C) = 16 Hz, *o*-CH, PPh_3_) 131.8 (s, *p*-CH, PPh^3^), 129.2 (m, *m*-CH, PPh_3_), 128.2 (d, ^1^J­(^31^P,^13^C) = 23 Hz, *ipso*-C, PPh_3_), 46.9 (dicarbollide C–H); ^31^P (162 MHz, C_6_D_6_) – 5.1 (s, PPh_3_); ^11^B NMR (128 MHz): δ −8.0–13.89 (4B, br m), −21.33 (1B, d, *J* = 119 Hz), −23.95 (2B, d, *J* = 69 Hz), −29.15 (1B, d, *J* = 50 Hz), −36.14 (1B, d, *J* = 102 Hz); ^11^B NMR (128 MHz): δ −8.0–13.89 (4B, br m), −21.33 (1B), −23.95 (2B), −29.07 (1B), −36.13 (1B); Anal. Calc. for C_38_H_41_B_9_P_2_Zn_1_: C, 63.18; H, 5.72. Found: C, 62.98; H, 5.83.

### Crystallographic Details

Single Crystal X-ray diffraction data were collected on a SuperNova, EosS2 diffractometer using Cu Kα (λ = 1.54184 Å) radiation. In each case, the crystals were maintained at 150 K during data collection. Using Olex2, the structures were solved with the olex2.solve6 structure solution program or ShelXT and refined with the ShelXL refinement package using least-squares minimization.^53^

Table S1 in the Supporting Information contains crystal and structural refinement data for the **1**, **2**, **3**, **5**, **6** and **7**.

### Computational Details

DFT calculations were run with Gaussian 16 (C.01).^54^ The Zn and P centers were described with the Stuttgart RECPs and associated basis sets, and the 6-31G** basis set^55^ was used for all other atoms (BS1).^56^ Initial BP86 optimizations (BP86/BS1) were performed using the “grid = ultrafine” option,^57^ with all stationary points being fully characterized via analytical frequency calculations as minima (i.e., all positive eigenvalues).

The Quantum Theory of Atoms in Molecules (QTAIM, AIMALL program^58^) and Natural Bonding Orbital (NBO7 ^59^) analyses were performed on the BP86-optmised geometry. The QTAIM topological analyses used wave function files obtained with Gaussian 16 (C.01) at the BP86/6-311++G**&cc-pVTZ (BS2) level (only the Zn atoms were described with cc-pVTZ). While NBO analyses were carried out with NBO v7.0 within Gaussian (C.01) at the same methodology level as the QTAIM calculations (BP86/BS2//BP86/BS1). Contour plots were generated in the AIMStudio package, using critical point (CP) visualization threshold values of 0.02*e* a_0_ Å^–3^ (solid line BCP = strong) and 0.005*e* a_0_ Å^–3^ (dashed line BCP = weak), when a_0_ is the Bohr radius. The NBO energies of donor–acceptor interactions (“Δ*E* ^(2)^”) between the various molecular fragments of the structures were estimated with second-order perturbation theory analysis of the Fock matrix in the NBO basis, as calculated by NBO7, with selected donor–acceptor NBO interactions provided. Wiberg bond indices (WBI) were also calculated using NBO v7.0.

## Supplementary Material



## Notes

Crystal structure data for compounds **1**–**3** and **5**–**7**. Electronic structural analysis for compounds **1**–**3**, the anion from **4** and **5**, **6**–**7** and compound **VII**. Data includes Cartesian coordinates and raw Hartree Energies (PDF)
