# Supplementary material for: Zinc Metallacarborane Chemistry
Source: Inorg Chem. 2026 Feb 6;65(7):4272–86. doi: 10.1021/acs.inorgchem.6c00184 (PMC12933884; doi:10.1021/acs.inorgchem.6c00184)
Supplement: Supplementary file 1 [file ic6c00184_si_001.pdf]

Zinc Metallacarborane Chemistry

Kerry R. Flanagan,[a] Chloe L. Johnson,[a] Joe C. Goodall,[a] Claire L. McMullin[a] and Andrew L. Johnson.[a*]*

a) Department of Chemistry, University of Bath, Claverton Down, Bath, BA2 7AY. UK.

Corresponding Authors

* Andrew L. Johnson (a.l.johnson@bath.ac.uk)

* Claire L. McMullin (cm2025@bath.ac.uk)

Contents

NMR Spectra 4

^{1}H NMR Spectra of [IMe_4·Zn{η^5-$C_2B_9H_{11}$}] (1) in CD_2Cl_2: 4

^{13}C NMR Spectra of [IMe_4·Zn{η^5-$C_2B_9H_{11}$}] (1) in CD_2Cl_2: 4

^{11}B NMR Spectra of [IMe_4·Zn{η^5-$C_2B_9H_{11}$}] (1) in CD_2Cl_2: 5

$^{11}B\{^1H\}$ NMR Spectra of [IMe_4·Zn{η^5-$C_2B_9H_{11}$}] (1) in CD_2Cl_2: 5

^{1}H NMR Spectra of [$I^iPr_2Me_2$·Zn{η^5-$C_2B_9H_{11}$}] (2) in CD_2Cl_2: 6

^{13}C NMR Spectra of [$I^iPr_2Me_2$·Zn{η^5-$C_2B_9H_{11}$}] (2)† in CD_2Cl_2: 6

^{11}B NMR Spectra of [$I^iPr_2Me_2$·Zn{η^5-$C_2B_9H_{11}$}] (2) in CD_2Cl_2: 7

$^{11}B\{^1H\}$ NMR Spectra of [$I^iPr_2Me_2$·Zn{η^5-$C_2B_9H_{11}$}] (2) in CD_2Cl_2: 7

^{1}H NMR Spectra of [IPr·Zn{η^5-$C_2B_9H_{11}$}] (3) in CD_2Cl_2: 8

^{13}C NMR Spectra of [IPr·Zn{η^5-$C_2B_9H_{11}$}] (3) in CD_2Cl_2: 8

^{11}B NMR Spectra of [IPr·Zn{η^5-$C_2B_9H_{11}$}] (3) in CD_2Cl_2: 9

$^{11}B\{^1H\}$ NMR Spectra of [IPr·Zn{η^5-$C_2B_9H_{11}$}] (3) in CD_2Cl_2: 9

^{1}H NMR Spectra of [I^tBu_2][Zn{η^3-$C_2B_9H_{11}$}$_2$] (4) in CD_2Cl_2: 10

^{13}C NMR Spectra of [I^tBu_2][Zn{η^3-$C_2B_9H_{11}$}$_2$] (4) in CD_2Cl_2: 10

^{11}B NMR Spectra of [I^tBu_2][Zn{η^3-$C_2B_9H_{11}$}$_2$] (4) in CD_2Cl_2: 11

$^{11}B\{^{1}H\}$ NMR Spectra of $[I^{t}Bu_2][Zn\{\eta^3\text{-}C_2B_9H_{11}\}_2]$ (4) in CD_2Cl_2: 11
^{1}H NMR Spectra of $[IAd_2][Zn\{\eta^3\text{-}C_2B_9H_{11}\}_2]$ (5) in CD_2Cl_2: 12
^{13}C NMR Spectra of $[IAd_2][Zn\{\eta^3\text{-}C_2B_9H_{11}\}_2]$ (5) in d8-THF: 12
^{11}B NMR Spectra of $[IAd_2][Zn\{\eta^3\text{-}C_2B_9H_{11}\}_2]$ (5) in CD_2Cl_2: 13
$^{11}B\{^{1}H\}$ NMR Spectra of $[IAd_2][Zn\{\eta^3\text{-}C_2B_9H_{11}\}_2]$ (5) in CD_2Cl_2: 13
^{1}H NMR Spectra of $[C_5H_5N\cdot Zn\{\mu^2\text{-}C_2B_9H_{11}\}]_2$ (6) in CD_2Cl_2: 14
^{13}C NMR Spectra of $[C_5H_5N\cdot Zn\{\mu^2\text{-}C_2B_9H_{11}\}]_2$ (6) in CD_2Cl_2: 14
^{11}B NMR Spectra of $[C_5H_5N\cdot Zn\{\mu^2\text{-}C_2B_9H_{11}\}]_2$ (6) in CD_2Cl_2: 15
$^{11}B\{^{1}H\}$ NMR Spectra of $[C_5H_5N\cdot Zn\{\mu^2\text{-}C_2B_9H_{11}\}]_2$ (6) in CD_2Cl_2: 15
^{1}H NMR Spectra of $[(Ph_3P)_2Zn\{\eta^3\text{-}C_2B_9H_{11}\}]$ (7) in C_6D_6: 16
^{13}C NMR Spectra of $[(Ph_3P)_2Zn\{\eta^3\text{-}C_2B_9H_{11}\}]$ (7) in C_6D_6: 16
^{11}B NMR Spectra of $[(Ph_3P)_2Zn\{\eta^3\text{-}C_2B_9H_{11}\}]$ (7) in C_6D_6: 17
$^{11}B\{^{1}H\}$ NMR Spectra of $[(Ph_3P)_2Zn\{\eta^3\text{-}C_2B_9H_{11}\}]$ (7) in C_6D_6: 17
^{31}P NMR Spectra of $[(Ph_3P)_2Zn\{\eta^3\text{-}C_2B_9H_{11}\}]$ (7) in C_6D_6: 18
Crystal and structural refinement data for the 1, 2, 3, 5, 6 and 7. 19
Table S1: Crystal and structural refinement data for the 1, 2, 3, 5, 6 and 7. 19
Electronic Structure Analysis 20
[(Cb)Zn(IMe4)]; **1** 20
NBO Charges 20
Wiberg Bond Indices 21
Second Order Perturbation Energies 21
Laplacian ($\nabla 2\rho$) Contour Plot 22
[(Cb)Zn(IiPrMe)]; **2** 25
NBO Charges 25
Wiberg Bond Indices 25
Second Order Perturbation Energies 26
Laplacian ($\nabla 2\rho$) Contour Plot 27
[(Cb)Zn(IDipp)]; **3** 30
NBO Charges 30
Wiberg Bond Indices 30
Second Order Perturbation Energies 31
Laplacian ($\nabla 2\rho$) Contour Plot 32
$[Zn(Cb)_2]^-$ anion from **4** and **5** 35
NBO Charges 35
Wiberg Bond Indices 36
Second Order Perturbation Energies 36

Laplacian ($\nabla 2\rho$) Contour Plot....37
[(Cb)Zn·Py]$_2$; **6**....40
NBO Charges....40
Wiberg Bond Indices....41
Second Order Perturbation Energies....41
Laplacian ($\nabla 2\rho$) Contour Plots....42
[(Cb)Zn(PPh$_3$)$_2$]; **7**....47
NBO Charges....47
Wiberg Bond Indices....48
Second Order Perturbation Energies....48
Laplacian ($\nabla 2\rho$) Contour Plot....49
[(Cb)Zn·NMe$_3$]$_2$; **VIII**....54
NBO Charges....54
Wiberg Bond Indices....55
Second Order Perturbation Energies....55
Laplacian ($\nabla 2\rho$) Contour Plots....56
Cartesian Coordinates and Raw Hartree Energies....62

NMR Spectra

^{1}H NMR Spectra of [IMe$_4$·Zn{η^5-$C_2B_9H_{11}$}] (1) in CD_2Cl_2:

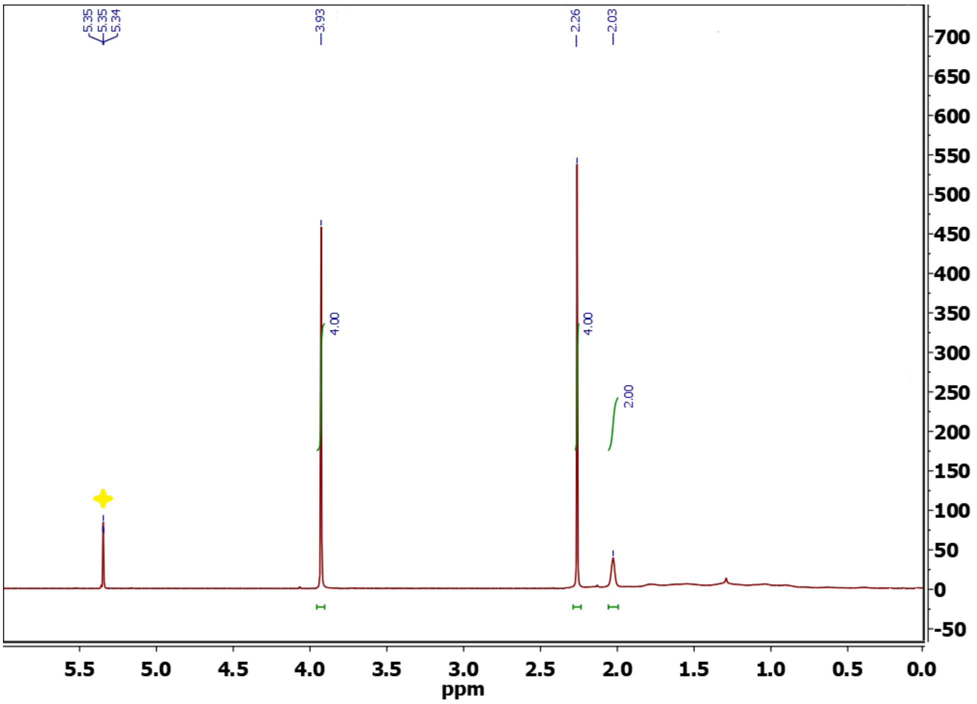

^{13}C NMR Spectra of [IMe$_4$·Zn{η^5-$C_2B_9H_{11}$}] (1) in CD_2Cl_2:

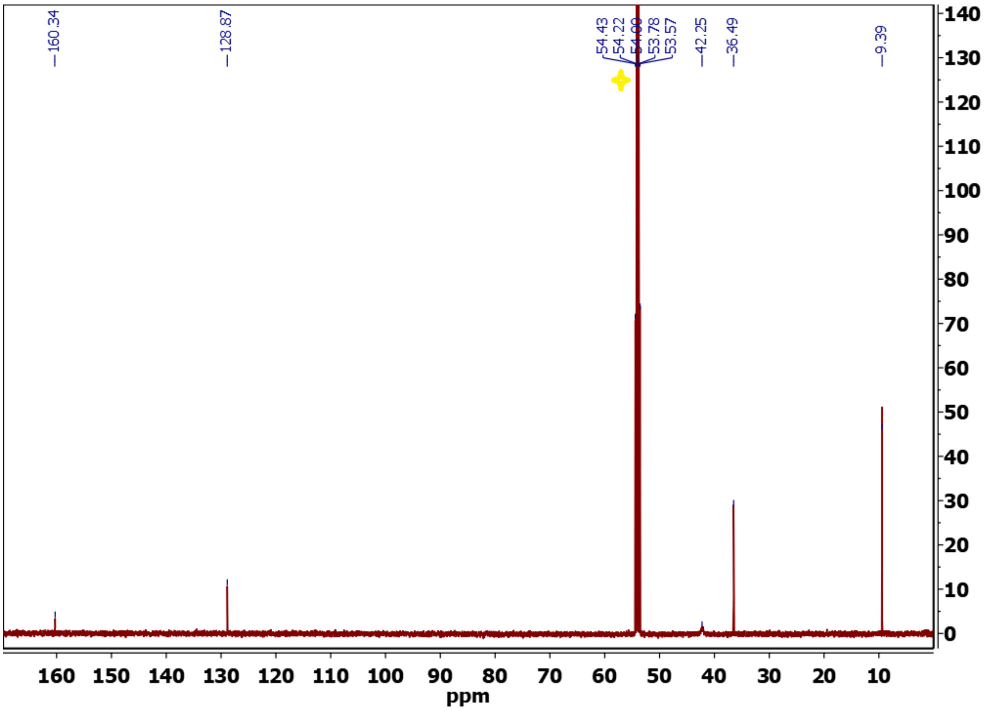

^{11}B NMR Spectra of [IMe_4·Zn{η^5-$C_2B_9H_{11}$}] (1) in CD_2Cl_2:

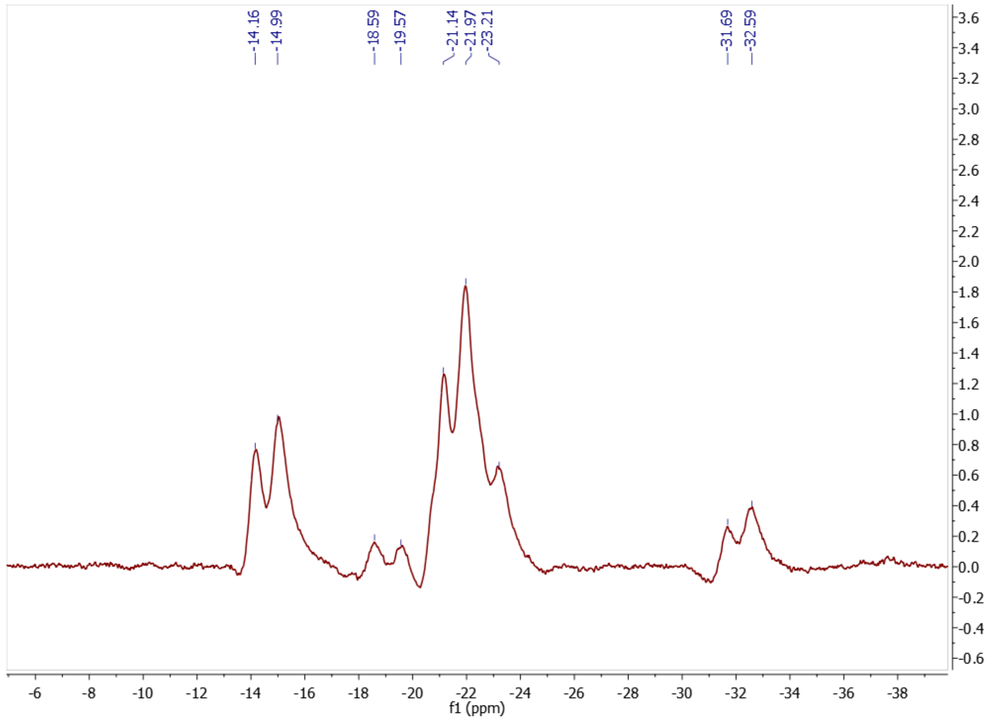

$^{11}B\{^1H\}$ NMR Spectra of [IMe_4·Zn{η^5-$C_2B_9H_{11}$}] (1) in CD_2Cl_2:

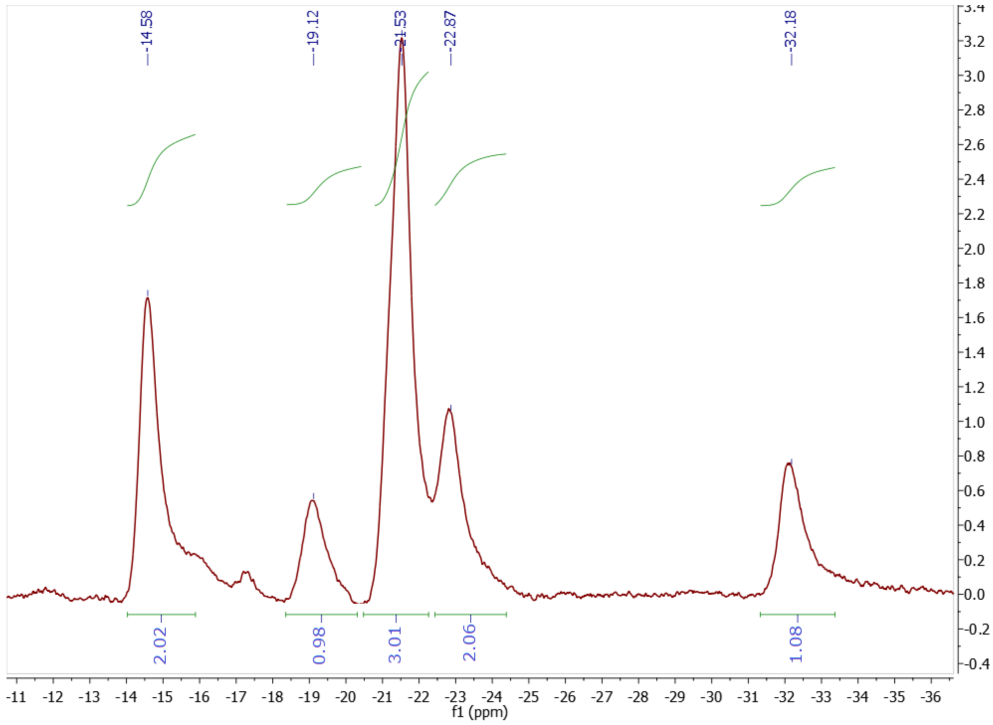

^{1}H NMR Spectra of [I^{i}Pr$_2$Me$_2$·Zn{η^5-C$_2$B$_9$H$_{11}$}] (2) in CD$_2$Cl$_2$:

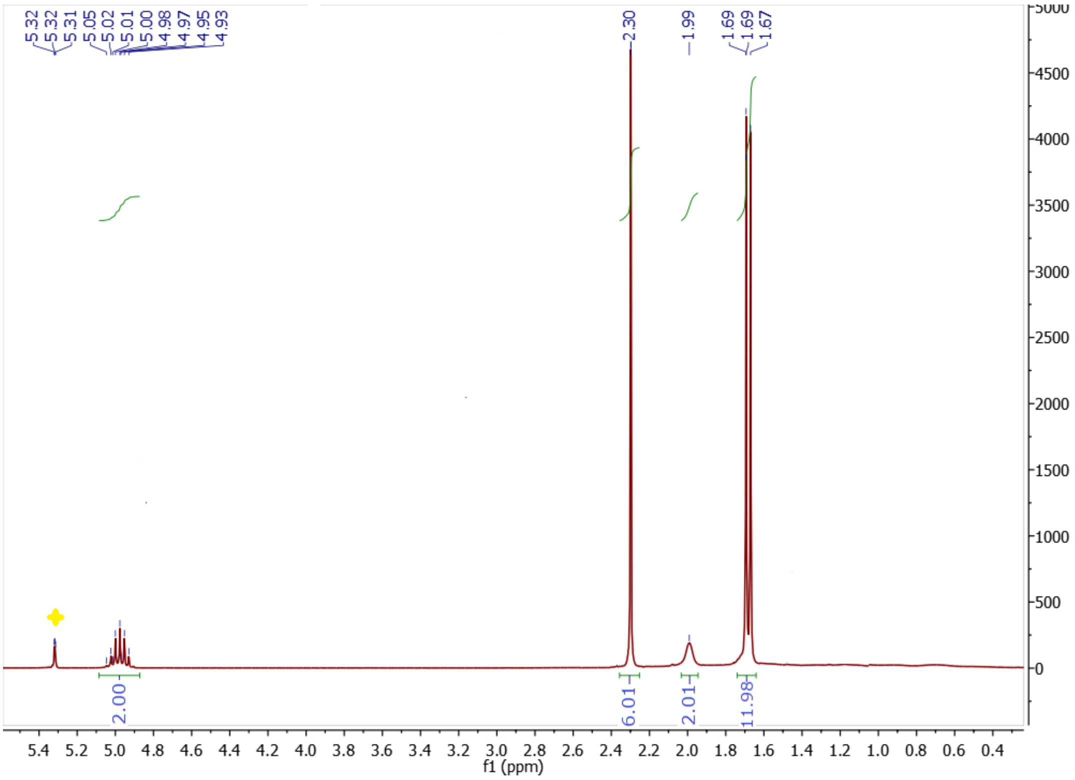

^{13}C NMR Spectra of [I^{i}Pr$_2$Me$_2$·Zn{η^5-C$_2$B$_9$H$_{11}$}] (2)† in CD$_2$Cl$_2$:

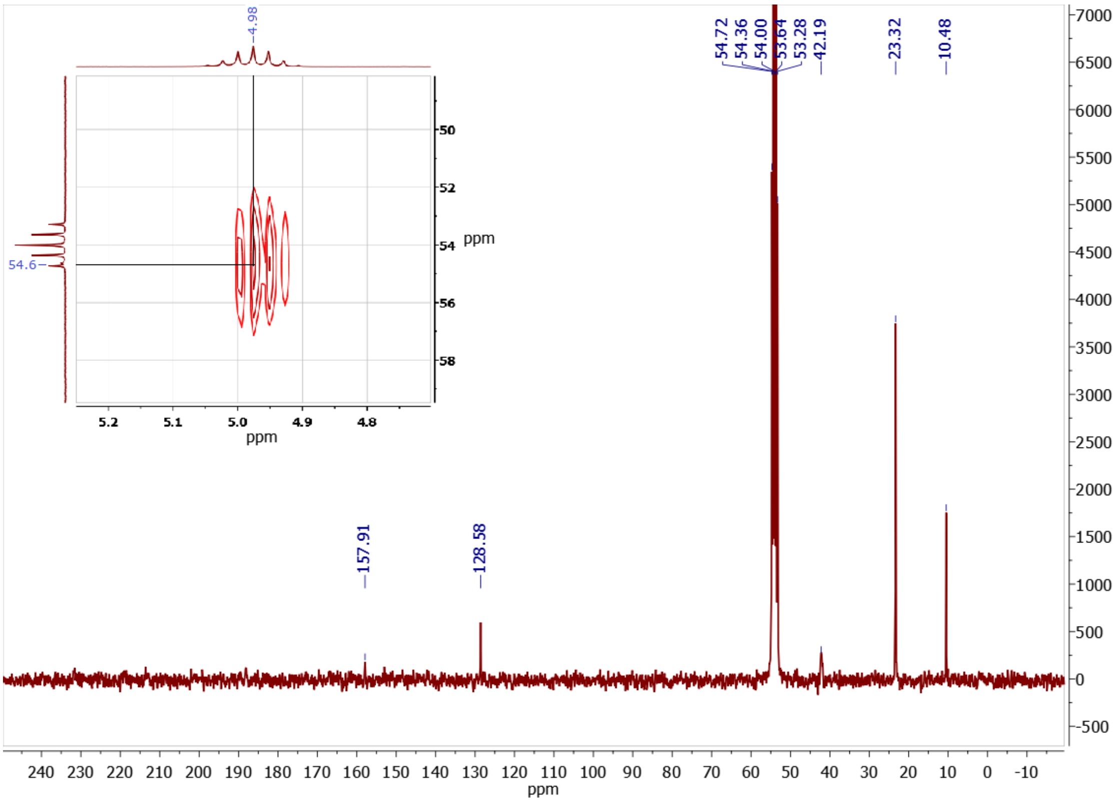

† Insert shows 2D ^{1}H-^{13}C (HSQC) correlation

^{11}B NMR Spectra of [$I^iPr_2Me_2$·Zn{η^5-$C_2B_9H_{11}$}] (2) in CD_2Cl_2:

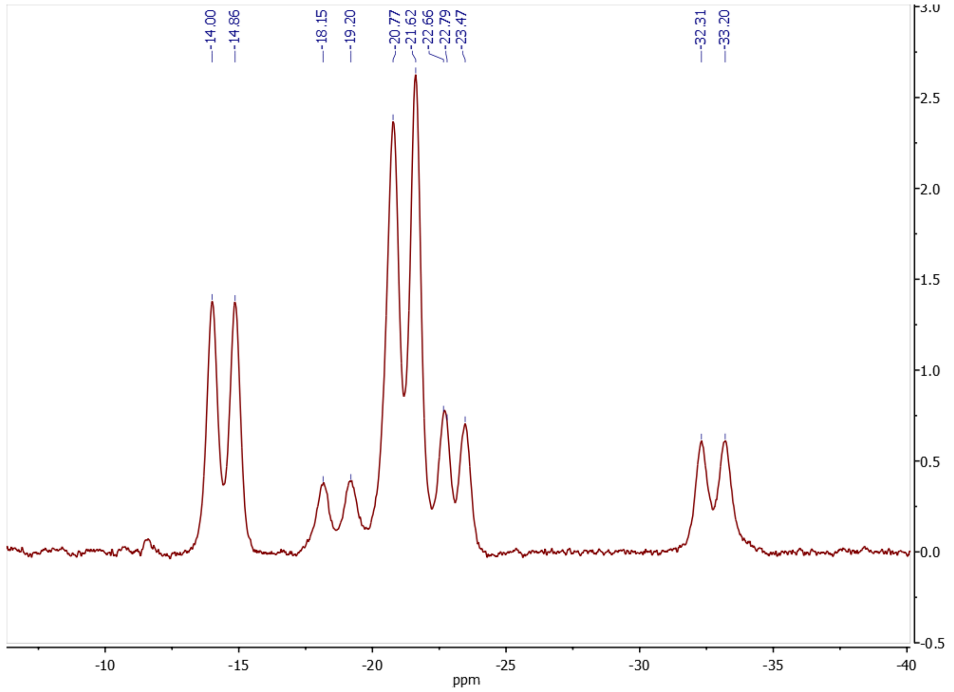

$^{11}B\{^1H\}$ NMR Spectra of [$I^iPr_2Me_2$·Zn{η^5-$C_2B_9H_{11}$}] (2) in CD_2Cl_2:

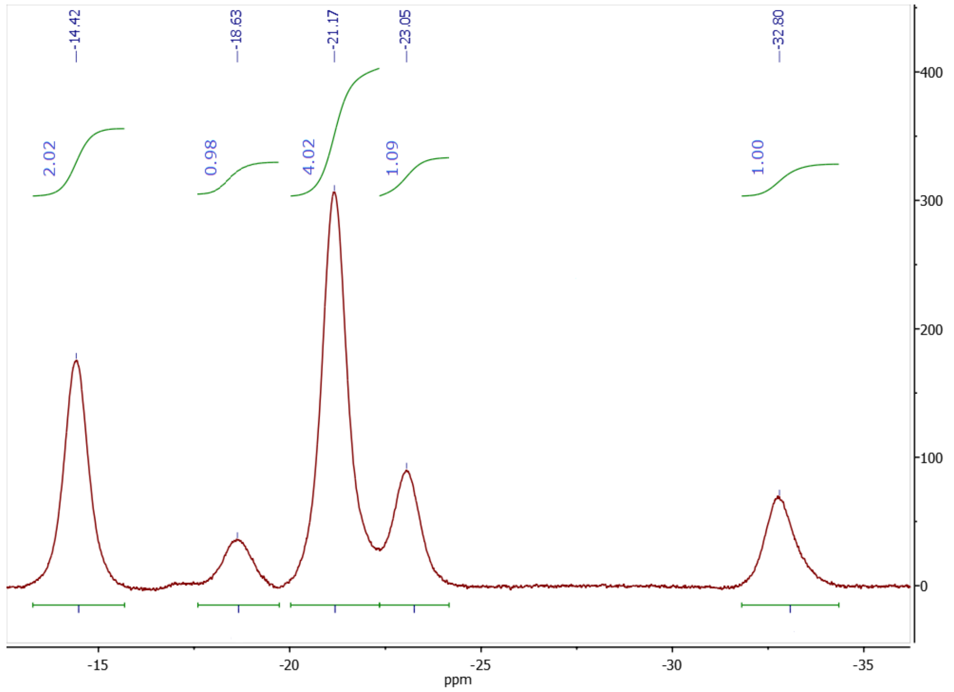

^{1}H NMR Spectra of [IPr▪Zn{η^{5}-$C_2B_9H_{11}$}] (3) in CD_2Cl_2:

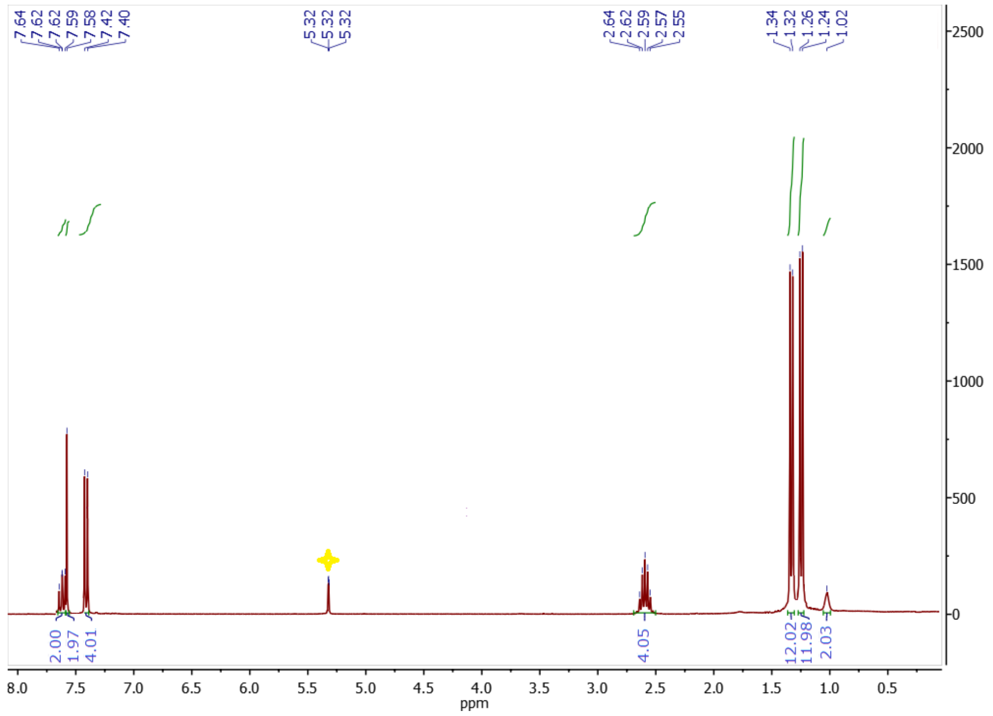

^{13}C NMR Spectra of [IPr▪Zn{η^{5}-$C_2B_9H_{11}$}] (3) in CD_2Cl_2:

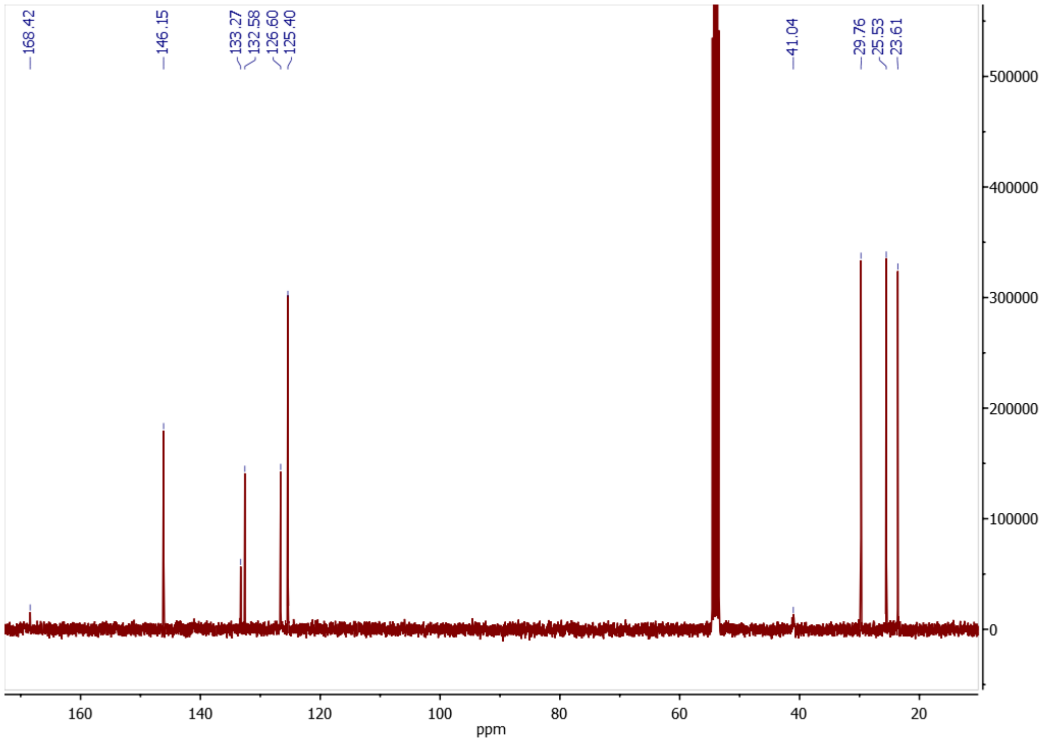

^{11}B NMR Spectra of [IPr·Zn{η^5-$C_2B_9H_{11}$}] (3) in CD_2Cl_2:

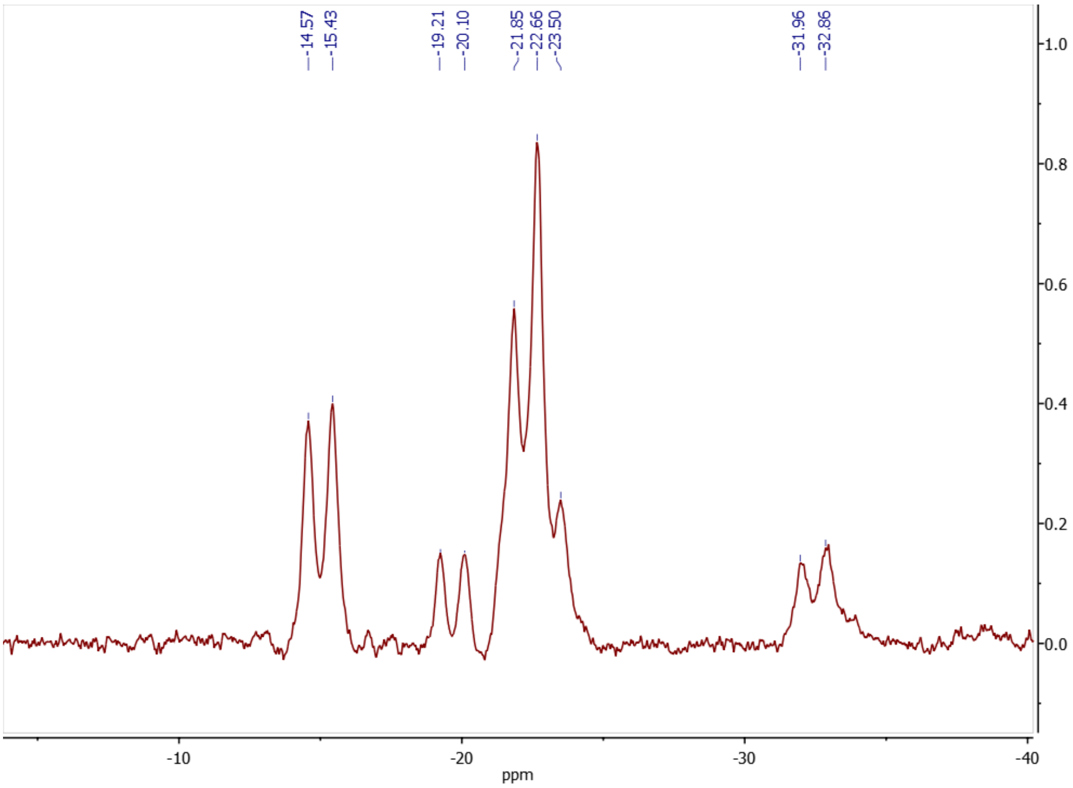

^{11}B{^{1}H} NMR Spectra of [IPr·Zn{η^5-$C_2B_9H_{11}$}] (3) in CD_2Cl_2:

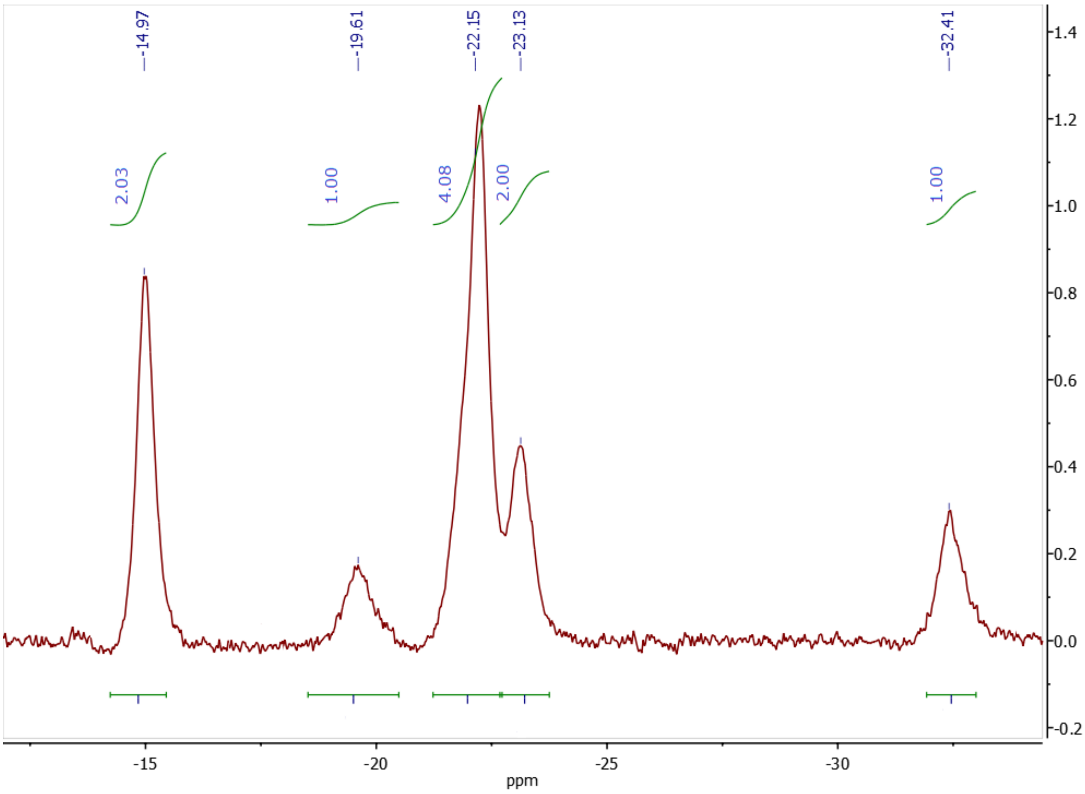

^{1}H NMR Spectra of $[I^{t}Bu_2][Zn\{\eta^3\text{-}C_2B_9H_{11}\}_2]$ (4) in CD_2Cl_2:

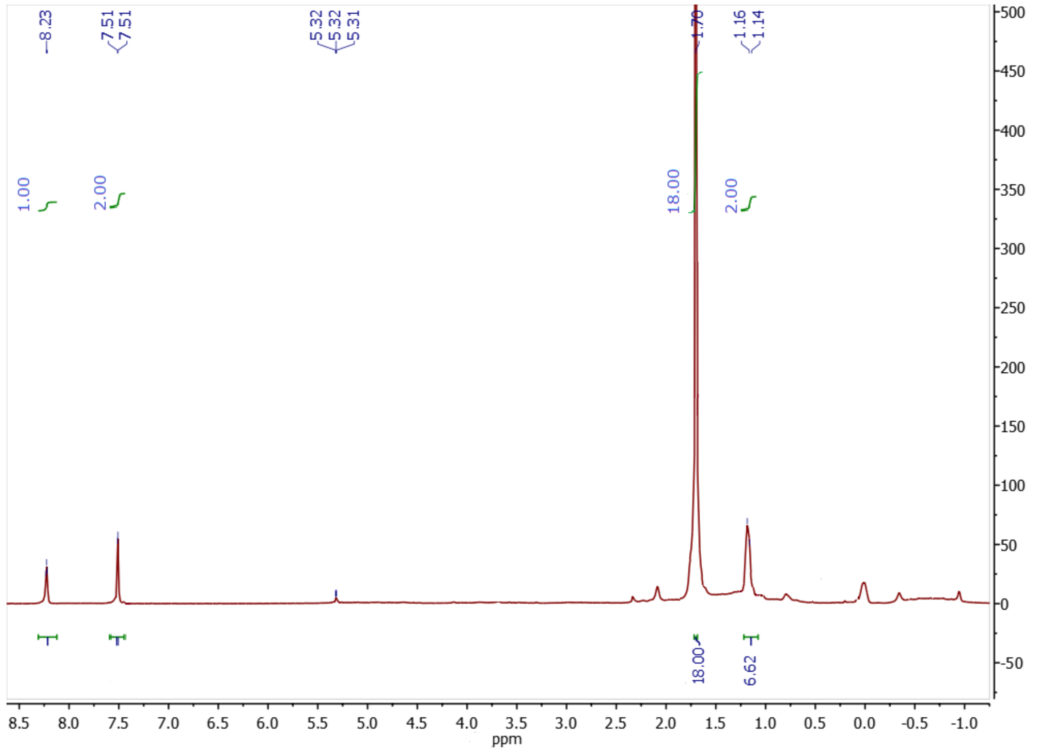

^{13}C NMR Spectra of $[I^{t}Bu_2][Zn\{\eta^3\text{-}C_2B_9H_{11}\}_2]$ (4) in CD_2Cl_2:

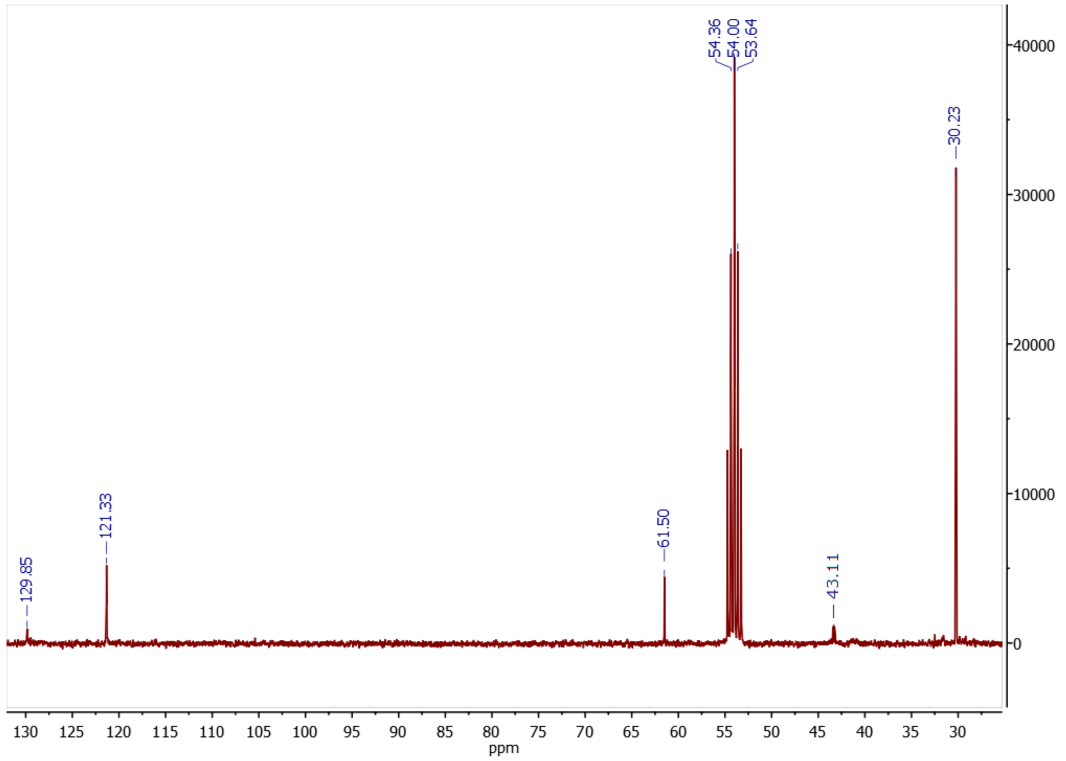

^{11}B NMR Spectra of $[I^tBu_2][Zn\{\eta^3\text{-}C_2B_9H_{11}\}_2]$ (4) in CD_2Cl_2:

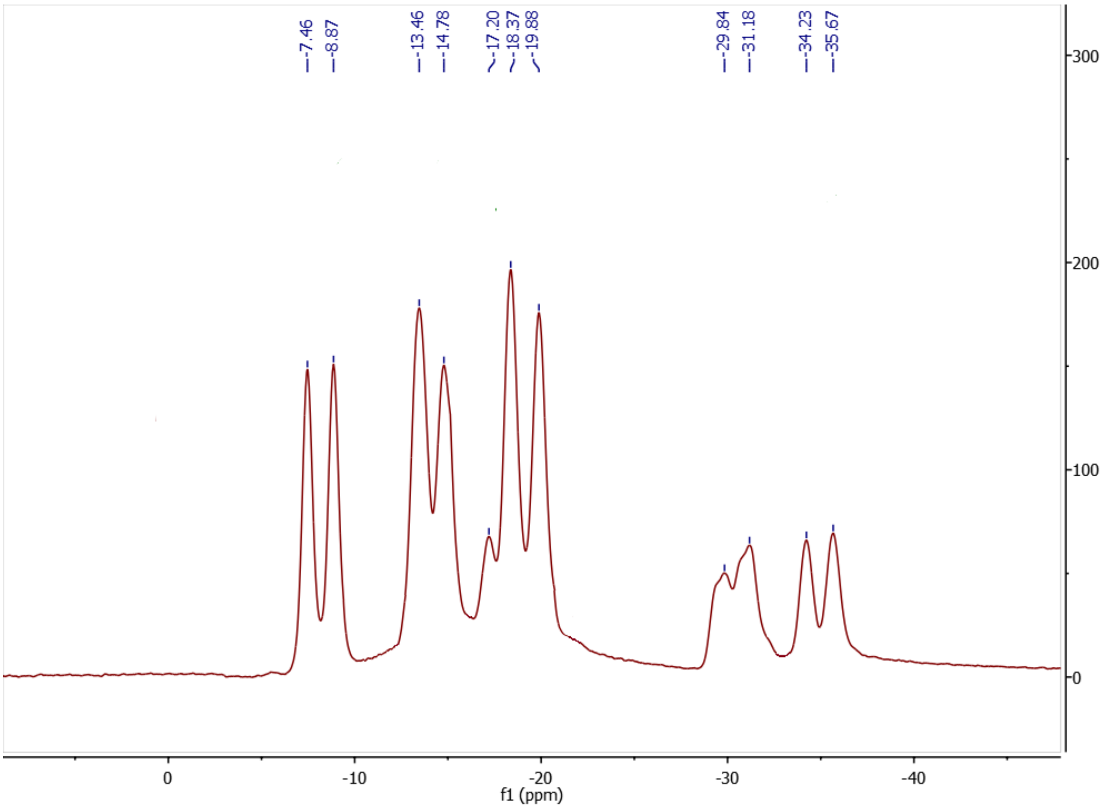

$^{11}B\{^1H\}$ NMR Spectra of $[I^tBu_2][Zn\{\eta^3\text{-}C_2B_9H_{11}\}_2]$ (4) in CD_2Cl_2:

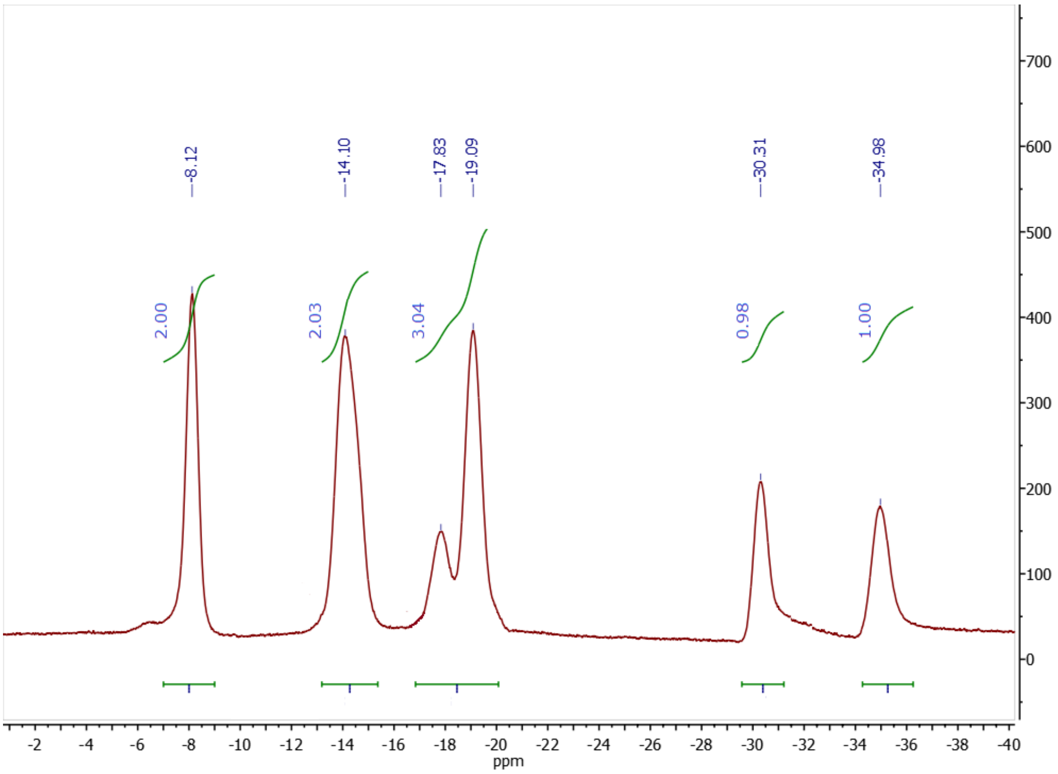

^{1}H NMR Spectra of $[IAd_2][Zn\{\eta^3\text{-}C_2B_9H_{11}\}_2]$ (5) in CD_2Cl_2:

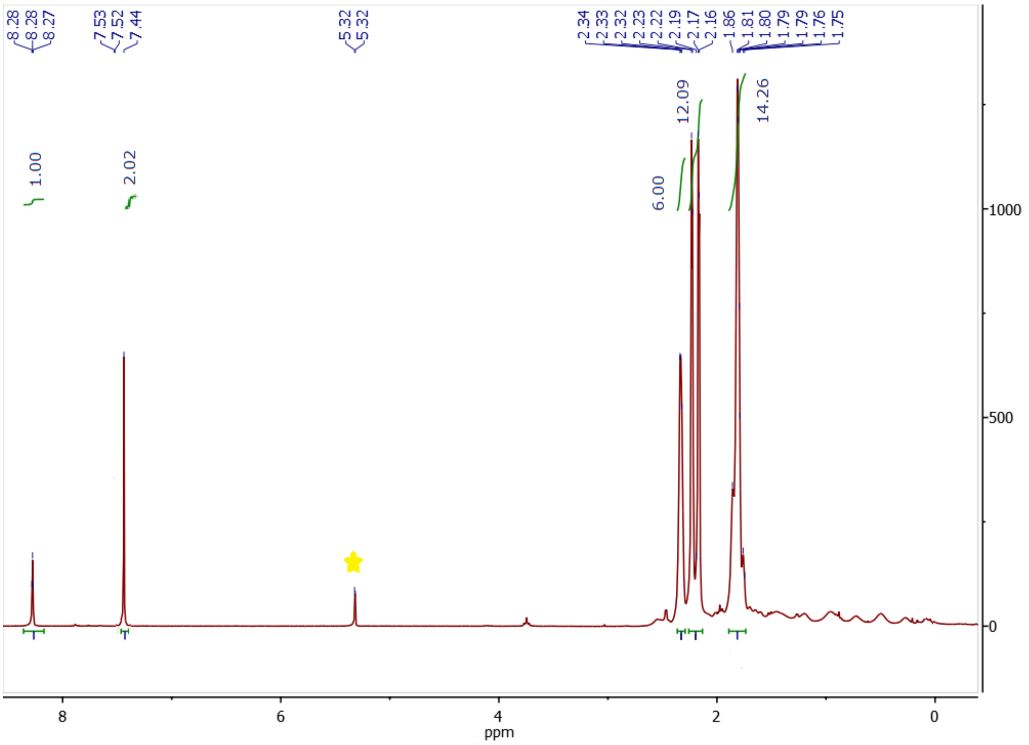

^{13}C NMR Spectra of $[IAd_2][Zn\{\eta^3\text{-}C_2B_9H_{11}\}_2]$ (5) in d8-THF:

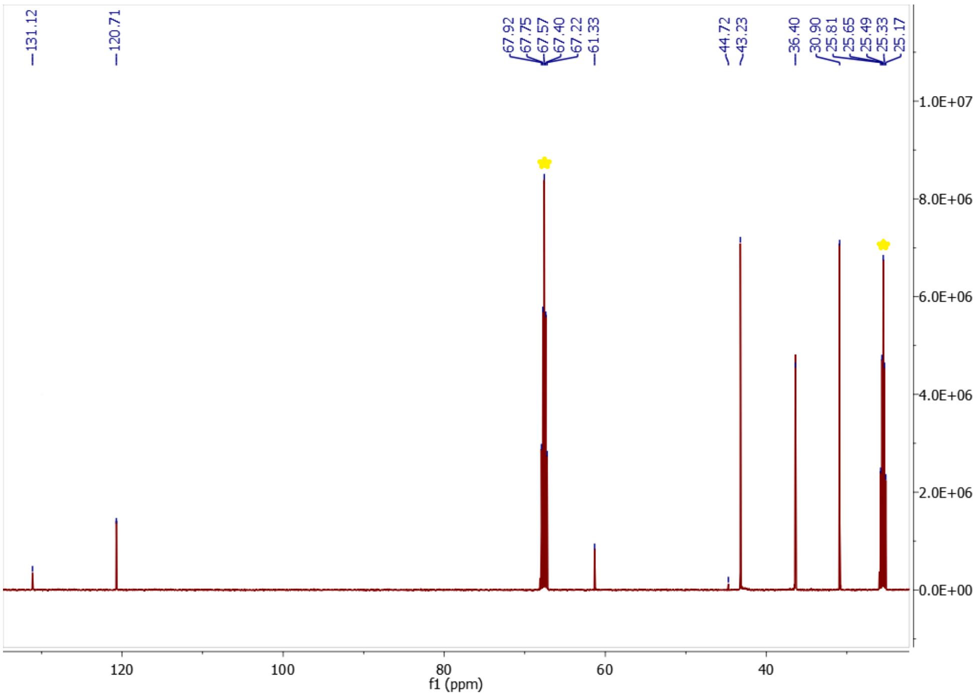

^{11}B NMR Spectra of $[IAd_2][Zn\{\eta^3\text{-}C_2B_9H_{11}\}_2]$ (5) in CD_2Cl_2:

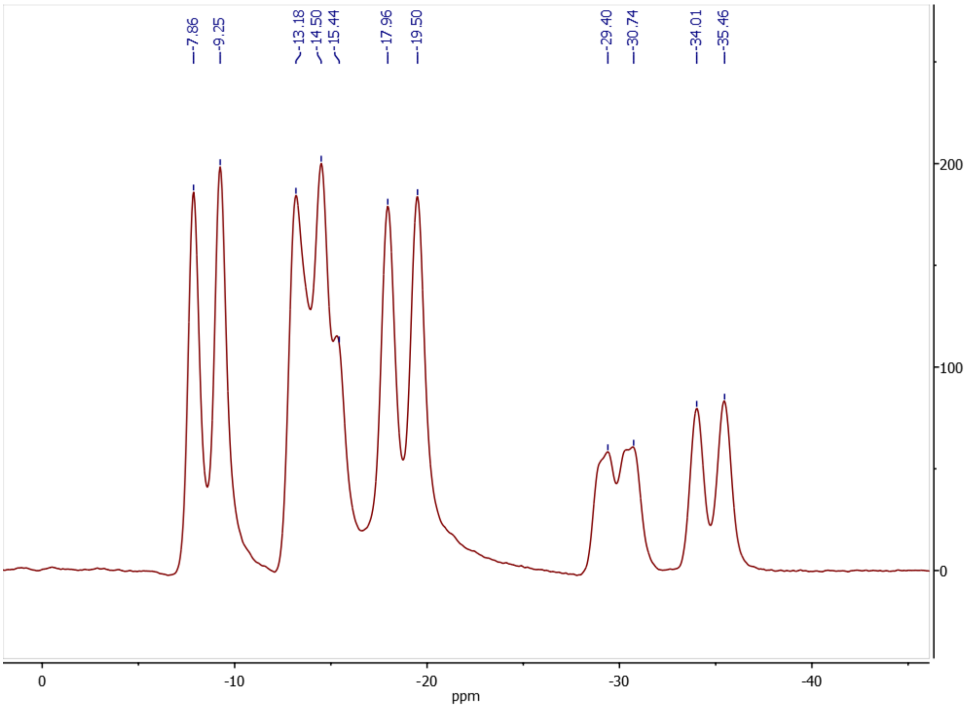

$^{11}B\{^1H\}$ NMR Spectra of $[IAd_2][Zn\{\eta^3\text{-}C_2B_9H_{11}\}_2]$ (5) in CD_2Cl_2:

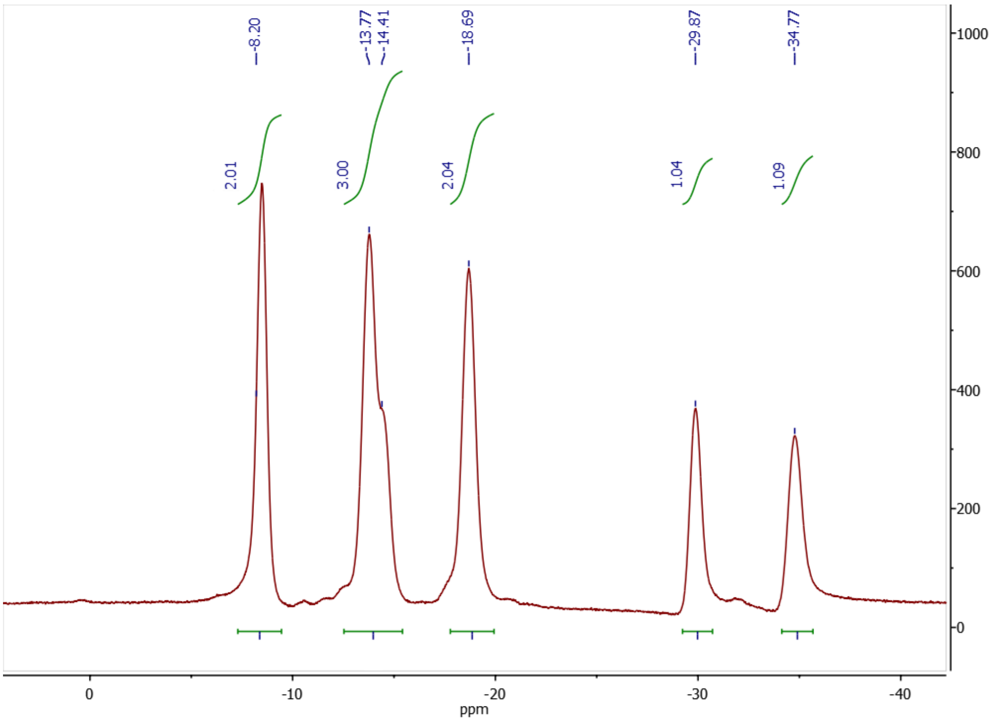

^{1}H NMR Spectra of $[C_5H_5N{\cdot}Zn\{\mu^2\text{-}C_2B_9H_{11}\}]_2$ (6) in CD_2Cl_2:

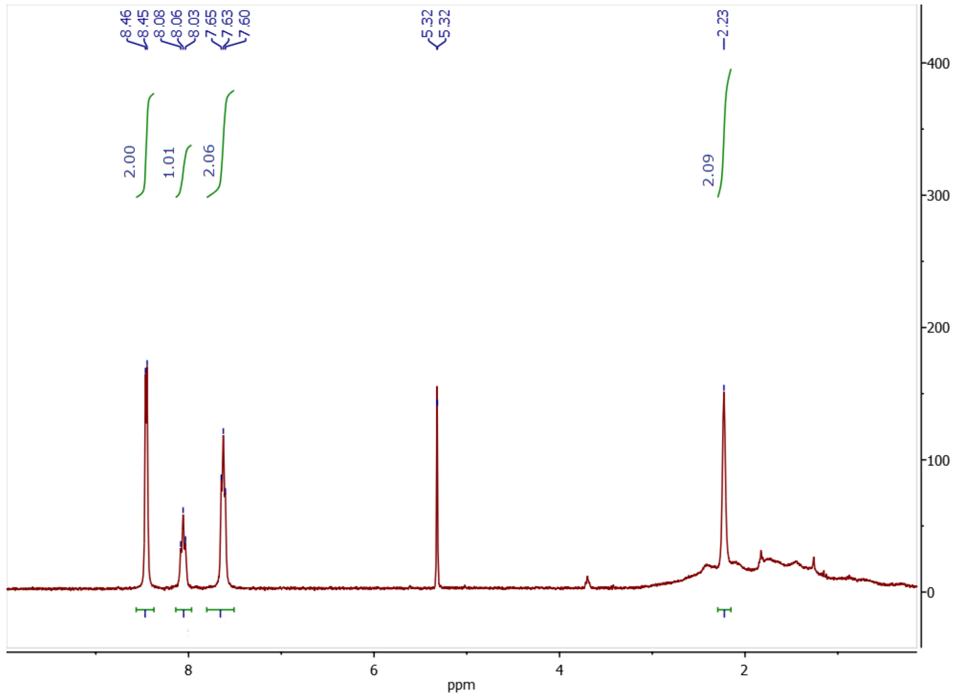

^{13}C NMR Spectra of $[C_5H_5N{\cdot}Zn\{\mu^2\text{-}C_2B_9H_{11}\}]_2$ (6) in CD_2Cl_2:

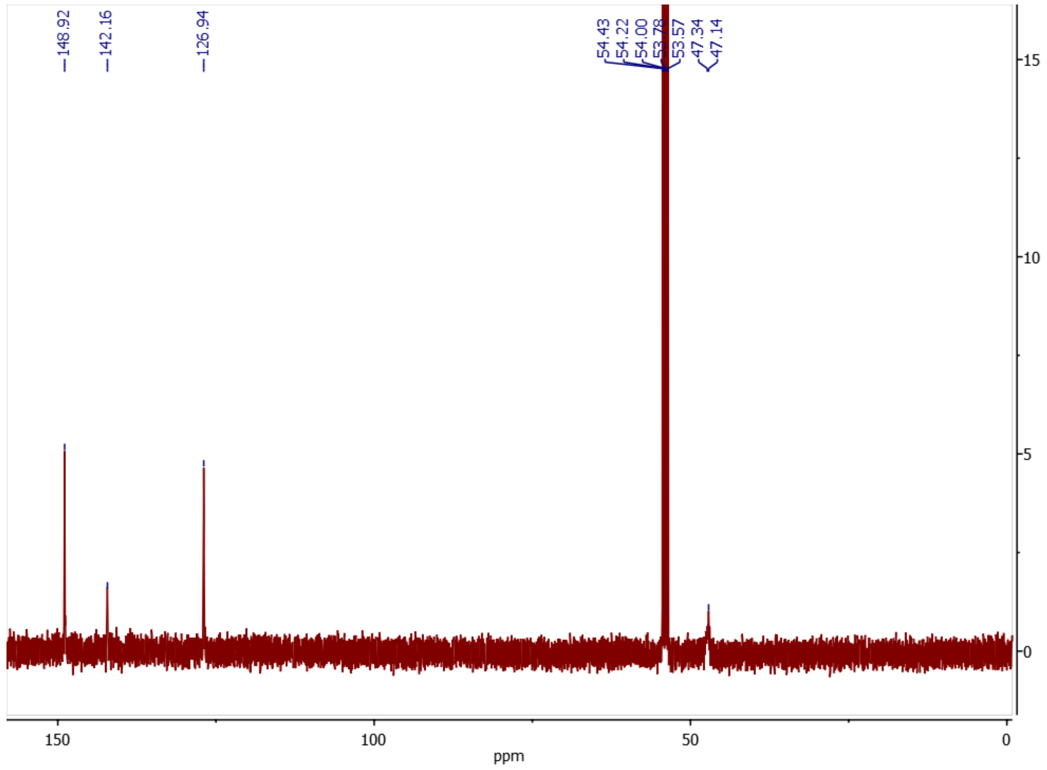

^{11}B NMR Spectra of $[C_5H_5N\cdot Zn\{\mu^2\text{-}C_2B_9H_{11}\}]_2$ (6) in CD_2Cl_2:

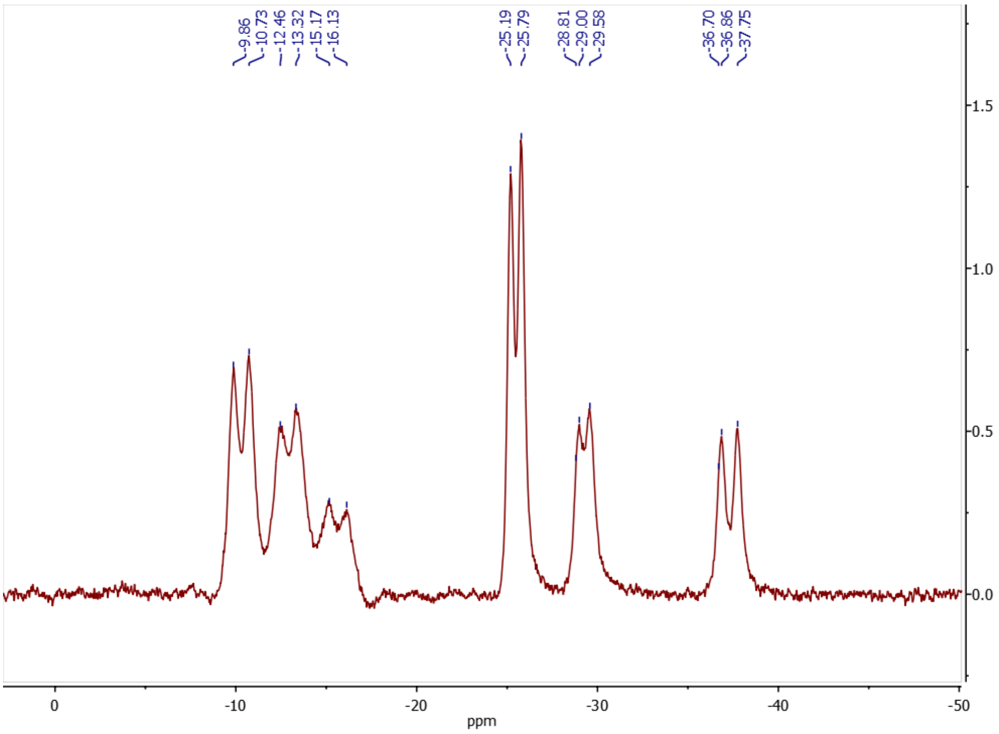

$^{11}B\{^1H\}$ NMR Spectra of $[C_5H_5N\cdot Zn\{\mu^2\text{-}C_2B_9H_{11}\}]_2$ (6) in CD_2Cl_2:

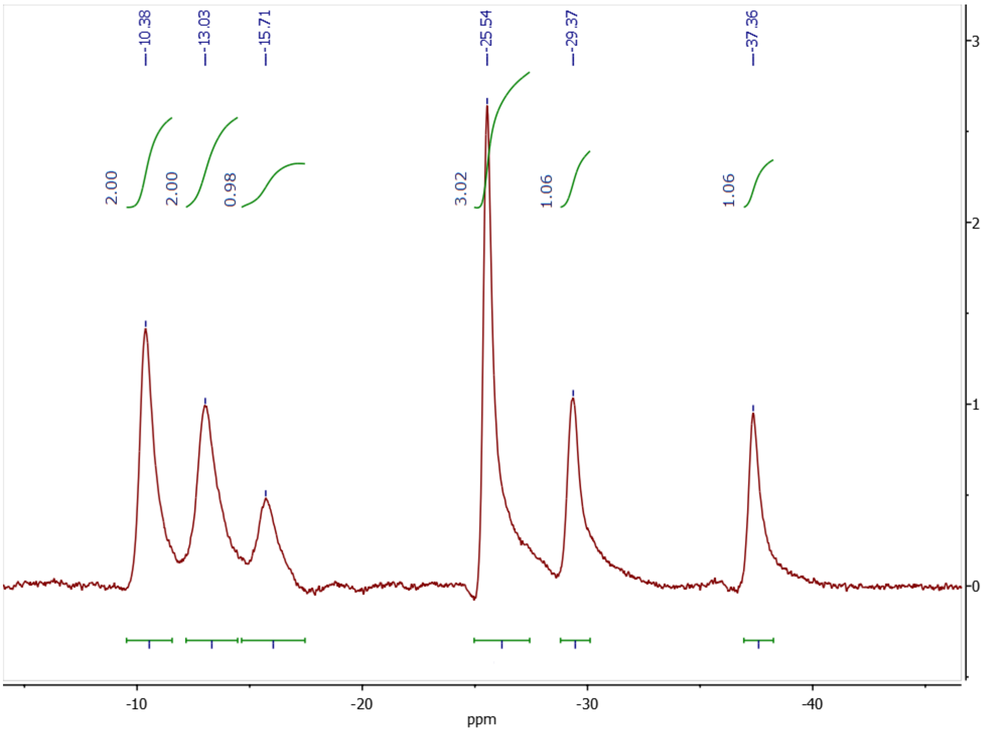

^{1}H NMR Spectra of $[(Ph_3P)_2Zn\{\eta^3\text{-}C_2B_9H_{11}\}]$ (7) in C_6D_6:

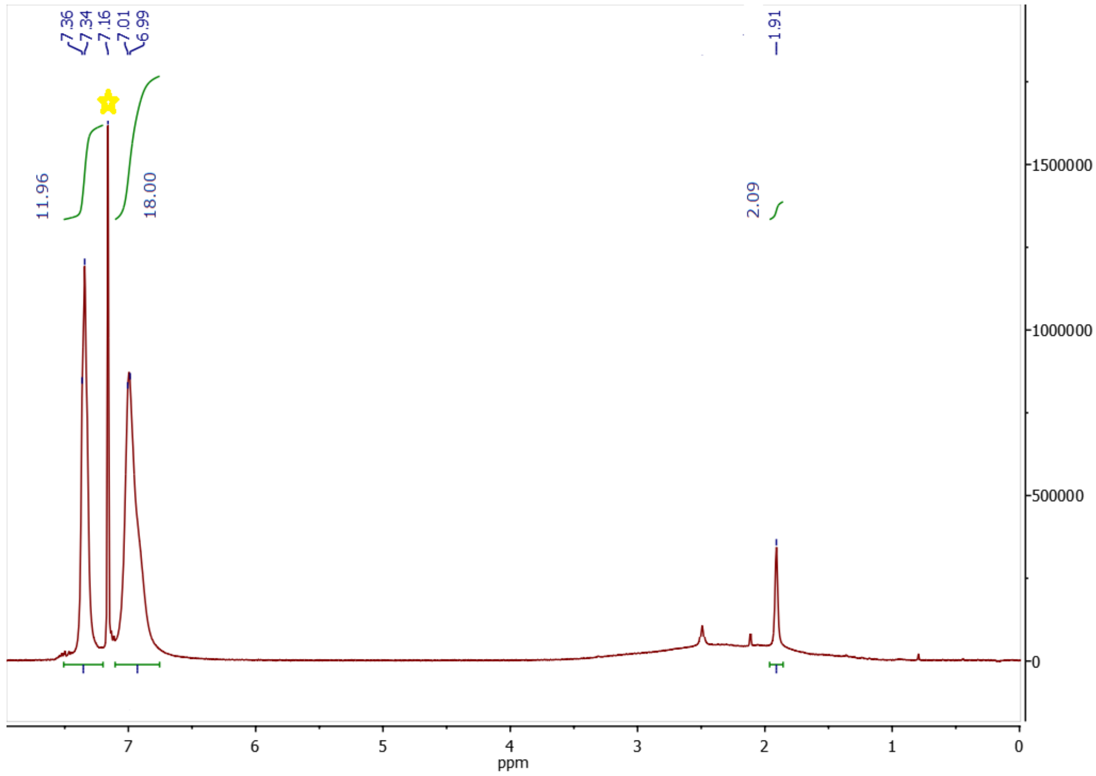

^{13}C NMR Spectra of $[(Ph_3P)_2Zn\{\eta^3\text{-}C_2B_9H_{11}\}]$ (7) in C_6D_6:

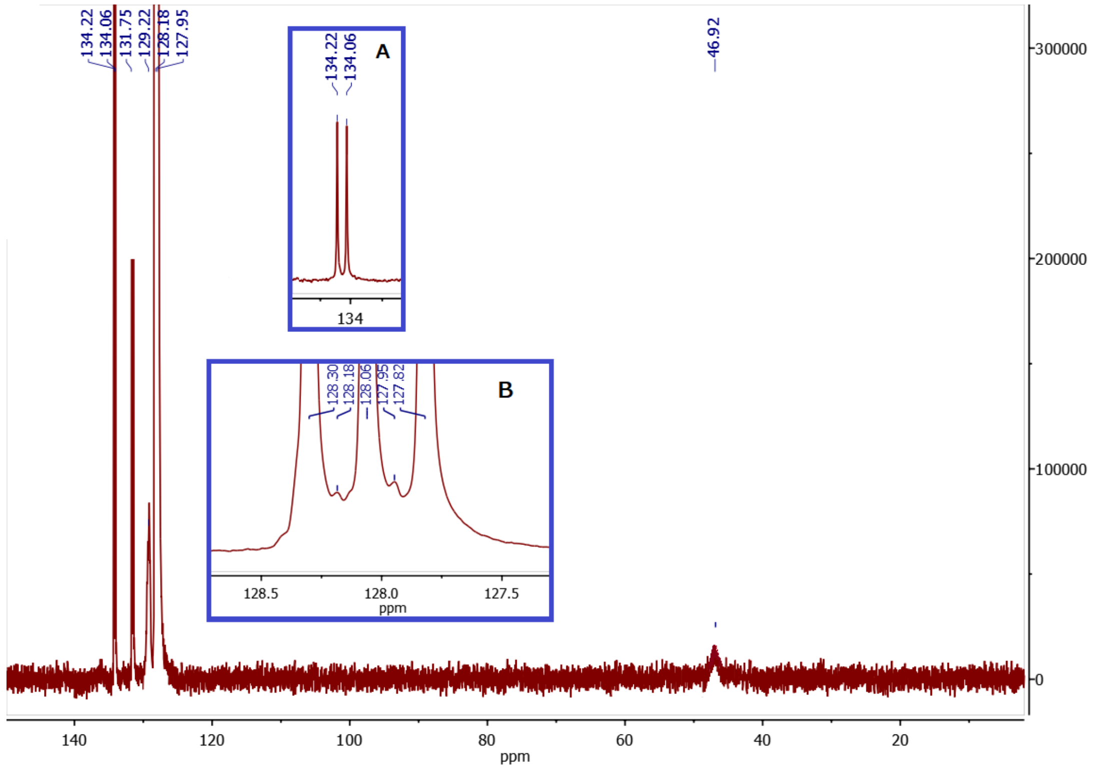

^{11}B NMR Spectra of $[(Ph_3P)_2Zn\{\eta^3\text{-}C_2B_9H_{11}\}]$ (7) in C_6D_6:

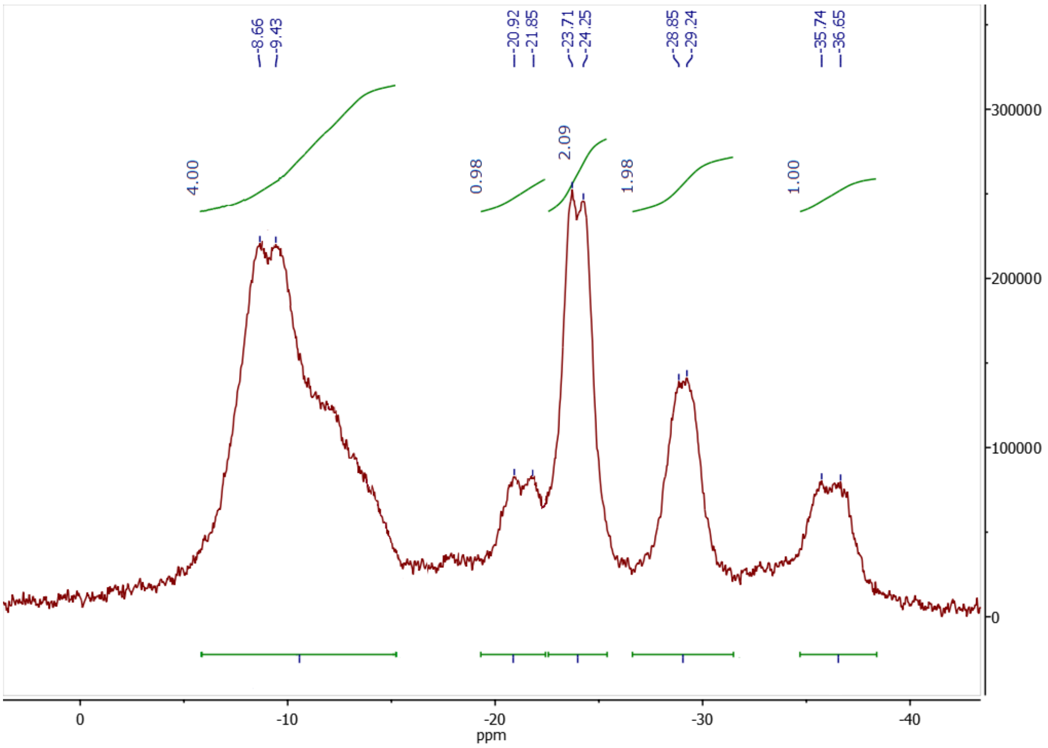

^{11}B{^{1}H} NMR Spectra of $[(Ph_3P)_2Zn\{\eta^3\text{-}C_2B_9H_{11}\}]$ (7) in C_6D_6:

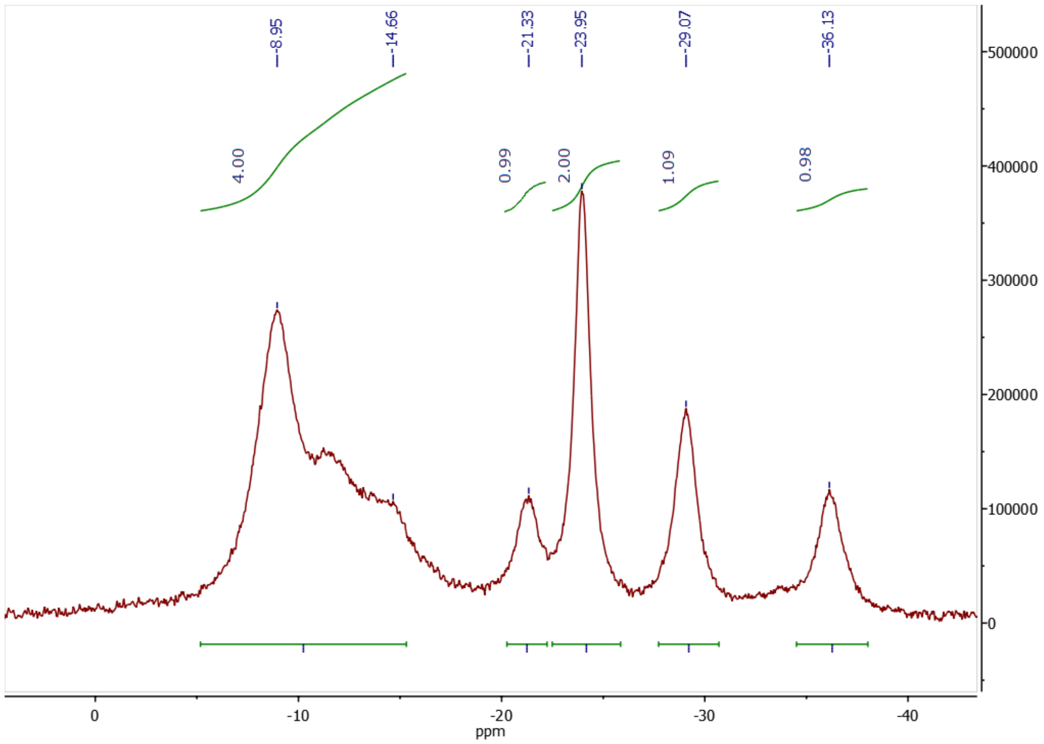

^{31}P NMR Spectra of $[(Ph_3P)_2Zn\{\eta^3\text{-}C_2B_9H_{11}\}]$ (7) in C_6D_6:

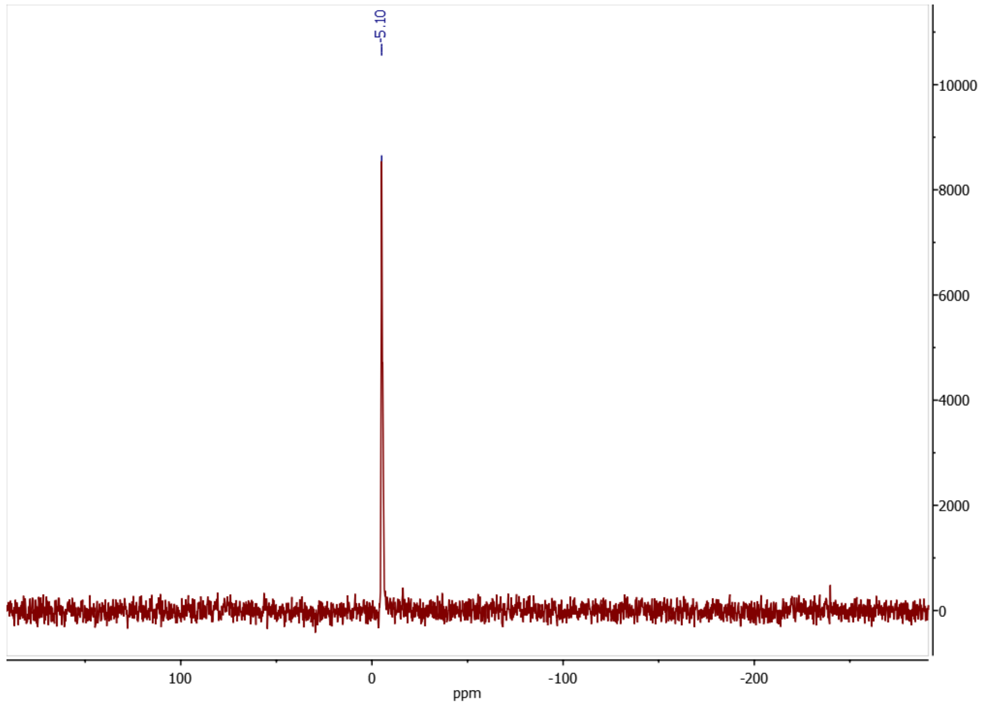

Crystal and structural refinement data for the 1, 2, 3, 5, 6 and 7.

Table S1: Crystal and structural refinement data for the 1, 2, 3, 5, 6 and 7.

Compound reference	**1**	**2**	**3**	**5**	**6**	**7**
Chemical formula	$C_9H_{23}B_9N_2Zn$	$C_{13}H_{31}B_9N_2Zn$	$C_{29}H_{47}B_9N_2Zn$	$2(C_{23}H_{33}N_2)•C_4H_{22}B_{18}Zn•3(CH_2Cl_2)$	$C_{14}H_{32}B_{18}N_2Zn_2•C_7H_8$	$C_{38}H_{41}B_9P_2Zn$
Formula Mass	321.95	378.06	586.34	1259.97	645.87	722.31
Crystal system	Triclinic	Monoclinic	Monoclinic	Triclinic	Monoclinic	Triclinic
Space group	$P\bar{1}$	*P21/c*	*P21/n*	$P\bar{1}$	*C2/c*	$P\bar{1}$
a/Å	8.2782(3)	7.45610(10)	10.7382(9)	10.9195(5)	27.2869(5)	10.7793(4)
b/Å	9.6396(3)	17.2664(2)	15.7668(9)	11.8383(7)	16.2707(3)	12.0472(5)
c/Å	11.1737(8)	15.7407(2)	20.526(2)	14.0597(7)	14.5022(3)	16.4126(6)
3α/°	84.694(5)	90	90	103.979(5)	90	84.128(3)
β/°	76.250(5)	101.5470(10)	99.164(9)	99.903(4)	92.8644(19)	75.813(3)
γ/°	68.233(3)	90	90	108.384(5)	90	63.798(4)
Unit cell volume/$Å^3$	804.33(7)	1985.44(4)	3430.8(5)	1611.72(16)	6430.6(2)	1853.97(14)
Temperature/K	150(2)	150(2)	150(2)	150(2)	150(2)	150(2)
Z	2	4	4	1	8	2
Radiation type	Cu Kα	Cu Kα	Cu Kα	Cu Kα	Cu Kα	Cu Kα
No. of reflections measured	5168	17300	8503	10745	21322	17318
No. of independent reflections	5168	3949	8503	6132	6345	7350
R_{int}	–	0.0197	–	0.0252	0.0301	0.0185
Final R_1 values ($I > 2\sigma(I)$)	0.0459	0.0258	0.0986	0.0743	0.0312	0.0291
Final $wR(F^2)$ values ($I > 2\sigma(I)$)	0.1238	0.0703	0.2638	0.2313	0.0836	0.0756
Final R_1 values (all data)	0.0488	0.0263	0.1239	0.0825	0.0366	0.0308
Final $wR(F^2)$ values (all data)	0.1249	0.0706	0.2761	0.2418	0.0872	0.0771
Goodness of fit on F^2	1.093	1.081	1.049	1.037	1.051	1.028
CCDC number	2487443	2487444	2487445	2487446	2487447	2487448

Electronic Structure Analysis

This section contains data from both NBO (Natural Bond Orbital) and QTAIM (Quantum Theory of Atoms in Molecules) calculations. To aid the discussion of each individual structure, and the bonding within, the analysis is ordered by structure and not the theoretical approach.

[(Cb)Zn(IMe_4)]; **1**

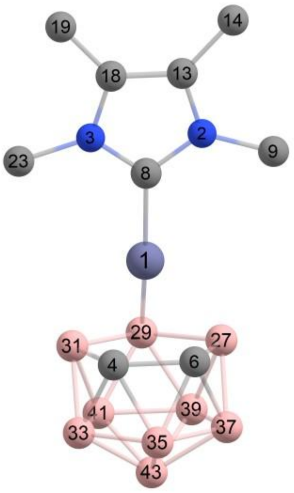

Figure S1: DFT-optimised structure of **1** with associated atom labels. Hydrogen atoms are omitted for clarity. Atom types are depicted by colour: carbon atoms in grey; nitrogen atoms in blue; boron atoms in pink; zinc atoms in glaucous (grey-blue-green).

NBO Charges

Table S2: Natural Population Analysis (NPA) charges of selected atoms in **1**.

Atom	NPA Charge
Zn1	1.46547
C8	–0.01346
C4	–0.63070
C6	–0.63072
B27	–0.28638
B29	–0.48366
B31	–0.28637

$\Sigma = -2.31783$

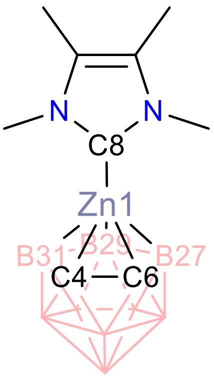

Wiberg Bond Indices

Table S3: Wiberg Bond Indices (WBI) of selected bonds in **1**.

Bond	WBI
Zn1–C8	0.4203
Zn1–C4	0.0142
Zn1–C6	0.0142
Zn1–B27	0.1040
Zn1–B29	0.1831
Zn1–B31	0.1041

$\Sigma = 0.4196$

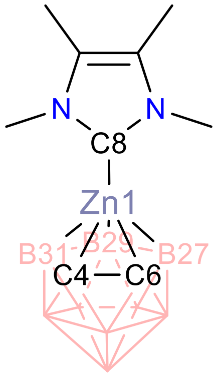

Second Order Perturbation Energies

There are no NBOs involving Zn and C8 or the 5 donor carborane atoms. However, some donor interactions from the ligand atoms to the vacant lone pair of Zn (NBO-79) are observed.

Table S4: Selected donor-acceptor interactions within **1**.

Donor NBO		Acceptor NBO	$\Delta E^{(2)}$ [in kcal/mol]
NBO-31	LP C8	NBO-79 LV Zn1	182.53
NBO-41	BD C4-B31	NBO-79 LV Zn1	12.74
NBO-44	BD C6-B27	NBO-79 LV Zn1	12.74
NBO-63	BD B27-B29	NBO-79 LV Zn1	71.69
NBO-65	BD B29-B31	NBO-79 LV Zn1	71.73

Note: Some interactions were also present between the other bonds of the carborane cage, but these were negligible / not significant.

Laplacian ($\nabla^2\rho$) Contour Plot

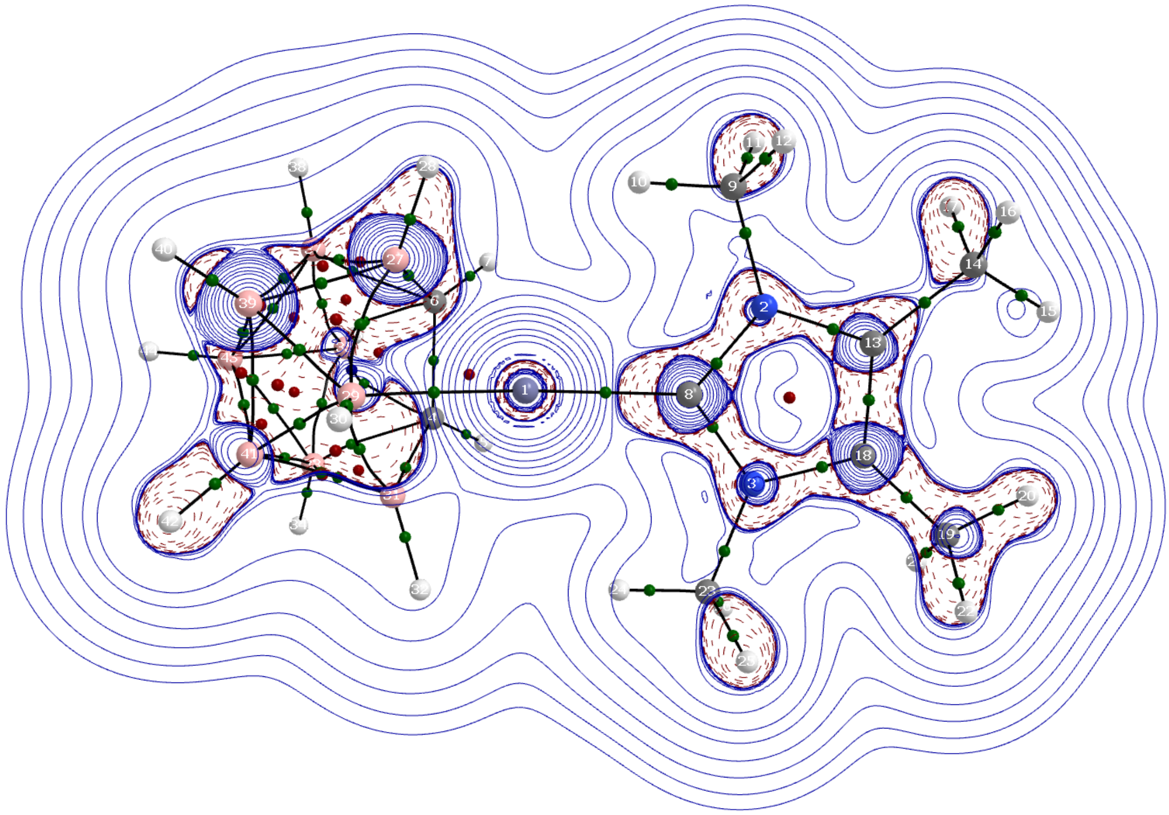

Figure S2: Contour plot of the Laplacian ($\nabla^2\rho(r)$) in the {C8-Zn1-B29} plane of the DFT-optimized **1**. Bond critical points (BCPs) are depicted as green spheres, ring critical points (RCPs) are depicted as dark red spheres.

There is only one bond path observed between Zn and the carborane (CB) cage, this is to B29, the central boron atom. This bond path, and that to the carbene carbon (C8) are ionic in nature ($\nabla^2\rho(r)$ values for both BCPs are positive). No other interactions were observed between Zn and the carborane ligand's atoms.

Table S5. Selected QTAIM atomic data for **1**.

Atom	q(A)	L(A)	N(A)	Vol(A)	%Loc(A)
**Zn1**	**+0.710586**	**+0.001448**	**19.289414**	**112.881602**	**93.116986**
N2	-1.075316	+0.000067	8.075316	77.173665	76.141585
N3	-1.075326	+0.000058	8.075326	77.192834	76.141557
**C4**	**-1.067090**	**+0.000010**	**7.067090**	**75.245849**	**68.838904**
**C6**	**-1.067115**	**+0.000051**	**7.067115**	**75.156360**	**68.838860**
**C8**	**+0.624716**	**-0.000097**	**5.375284**	**75.661884**	**67.650829**
C9	+0.263649	-0.000115	5.736351	66.617009	66.578696
C13	+0.334823	-0.000053	5.665177	65.551927	64.472141
C14	-0.013332	-0.000048	6.013332	71.630329	66.492179
C18	+0.334841	-0.000064	5.665159	65.489083	64.472189
C19	-0.013323	-0.000027	6.013323	71.666645	66.491678
C23	+0.263619	-0.000160	5.736381	66.593817	66.578522
**B27**	**+0.745779**	**+0.000001**	**4.254221**	**45.372566**	**62.239872**
**B29**	**+0.362654**	**+0.000086**	**4.637346**	**52.637450**	**61.230134**
**B31**	**+0.746487**	**+0.000416**	**4.253513**	**45.318656**	**62.242761**
B33	+0.794827	+0.000406	4.205173	43.066408	62.331239
B35	+0.936620	-0.000094	4.063380	40.893497	63.108287
B37	+0.794133	-0.000149	4.205867	43.130455	62.328070
B39	+0.538471	+0.000280	4.461529	47.505431	61.221906
B41	+0.537770	-0.000113	4.462230	47.516167	61.219977
B43	+0.550634	+0.000139	4.449366	48.116996	61.285975

Table S6. Selected QTAIM BCP data for **1**.

BCP	ρ(r)	$\nabla^2\rho(r)$	ε	G(r)	V(r)
**Zn1 - C8**	**0.108404**	**+0.234267**	**0.030545**	**+0.102161**	**-0.145755**
C4 - B31	0.129751	+0.015664	0.777579	+0.115065	-0.226213
C6 - B27	0.129755	+0.015676	0.777453	+0.115073	-0.226227
C4 - C6	0.204459	-0.295905	0.338455	+0.068022	-0.210019
B27 - B29	0.107480	-0.089436	3.795364	+0.029040	-0.080439
**Zn1 - B29**	**0.083212**	**+0.034921**	**1.125261**	**+0.042947**	**-0.077163**
B29 - B31	0.107477	-0.089420	3.797220	+0.029040	-0.080436
B29 - B41	0.113942	-0.106540	2.920514	+0.036133	-0.098901
B29 - B39	0.113942	-0.106543	2.920363	+0.036133	-0.098902
B27 - B39	0.115154	-0.110707	3.284047	+0.035864	-0.099404
B31 - B41	0.115157	-0.110721	3.283320	+0.035865	-0.099409
C4 - B33	0.121390	+0.047604	2.705480	+0.111016	-0.210130
C4 - B35	0.118744	-0.077431	2.678002	+0.071165	-0.161688
B39 - B43	0.112758	-0.100891	3.676736	+0.035312	-0.095846
B33 - B41	0.117043	-0.118119	3.042838	+0.036164	-0.101857
B31 - B33	0.109845	-0.075254	6.148665	+0.036708	-0.092230
B33 - B35	0.115758	-0.102147	4.256185	+0.038480	-0.102496
C6 - B35	0.118745	-0.077436	2.677977	+0.071165	-0.161689
C6 - B37	0.121391	+0.047612	2.705726	+0.111018	-0.210133
B35 - B37	0.115760	-0.102150	4.256097	+0.038481	-0.102499
B27 - B37	0.109843	-0.075253	6.147523	+0.036709	-0.092232
B37 - B39	0.117043	-0.118120	3.042747	+0.036164	-0.101858
B41 - B43	0.112757	-0.100886	3.676946	+0.035311	-0.095844
B35 - B43	0.119445	-0.122547	3.282226	+0.039110	-0.108857
B37 - B43	0.116478	-0.110951	3.727111	+0.037174	-0.102085
B39 - B41	0.110623	-0.094285	4.262615	+0.034428	-0.092428
B33 - B43	0.116477	-0.110946	3.727177	+0.037173	-0.102082

[(Cb)Zn(IiPrMe)]; 2

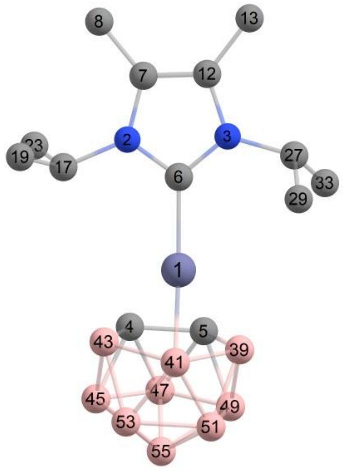

Figure S3: DFT-optimised structure of **2** with associated atom labels. Hydrogen atoms are omitted for clarity. Atom types are depicted by colour: carbon atoms in grey; nitrogen atoms in blue; boron atoms in pink; zinc atoms in glaucous (grey-blue-green).

NBO Charges

Table S7: Natural Population Analysis (NPA) charges of selected atoms in **2**.

Atom	NPA Charge
Zn1	1.46514
C6	−0.01590
H32	0.23543
C4	−0.63074
C5	−0.62616
B39	−0.28944
B41	−0.49647
B43	−0.28500

Σ = −2.32781

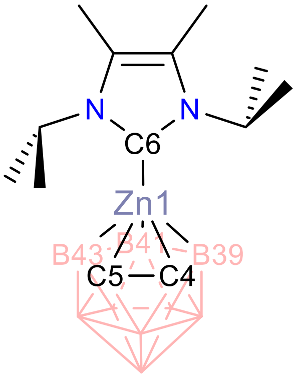

Wiberg Bond Indices

Table S8: Wiberg Bond Indices (WBI) of selected bonds in **2**.

Bond	WBI
Zn1–C6	0.4184
Zn1–H32	0.0036
Zn1–C4	0.0128
Zn1–C5	0.0145
Zn1–B39	0.1065
Zn1–B41	0.1799
Zn1–B43	0.0951

Σ = 0.4088

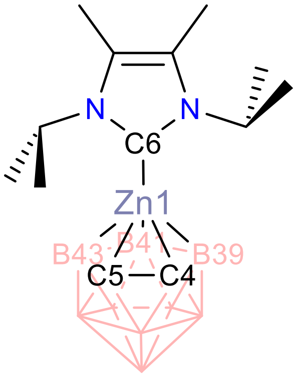

Second Order Perturbation Energies

Again, there are no NBOs involving Zn and C6 or the 5 donor carborane atoms. However, some donor interactions from the ligand atoms to the vacant lone pair of Zn (NBO-59) are observed.

Table S9: Selected donor-acceptor interactions within **2**.

Donor NBO		Acceptor NBO	$\Delta E^{(2)}$ [in kcal/mol]
NBO-36	LP C6	NBO-95 LV Zn1	171.01
NBO-45	BD C4-B43	NBO-95 LV Zn1	10.68
NBO-48	BD C5-B39	NBO-95 LV Zn1	11.07
NBO-79	BD B39-B41	NBO-95 LV Zn1	64.28
NBO-81	BD B41-B43	NBO-95 LV Zn1	63.19

Note: Again, some interactions were also present between the other bonds of the carborane cage, but these were negligible / not significant.

Laplacian ($\nabla^2\rho$) Contour Plot

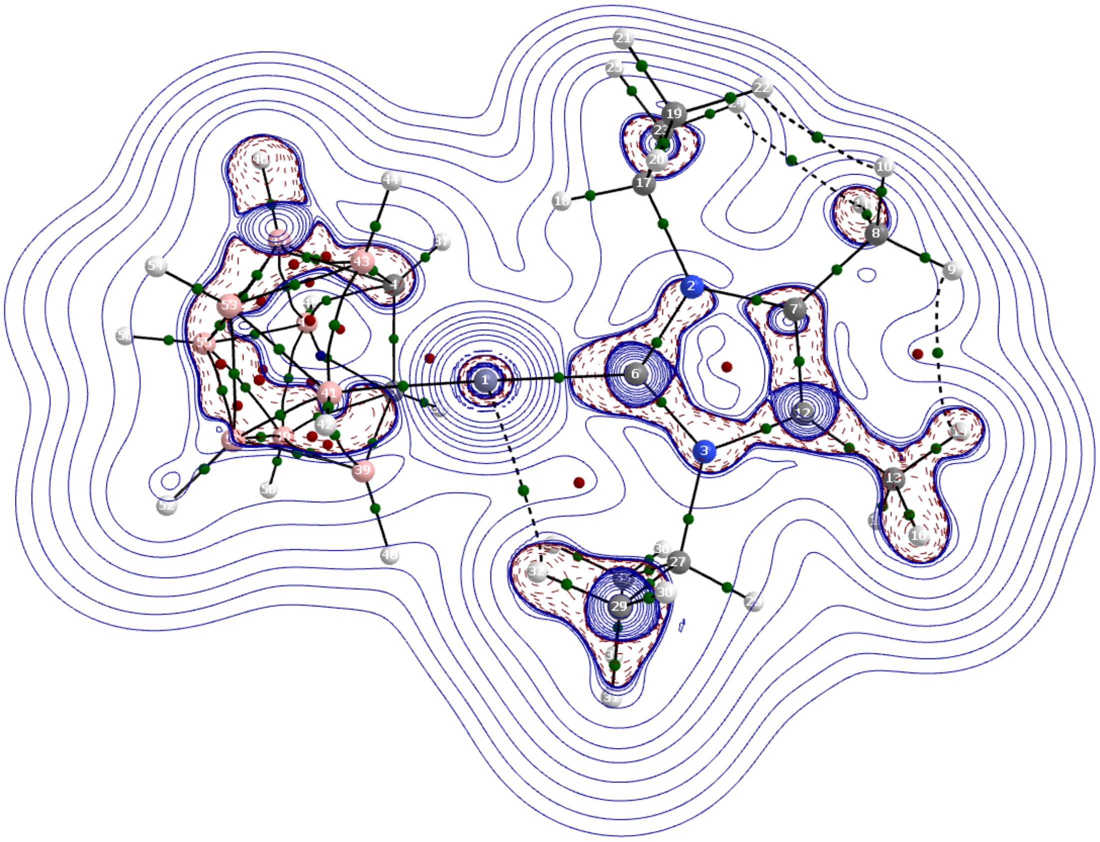

Figure S4: Contour plot of the Laplacian ($\nabla^2\rho(r)$) in the {C6-Zn1-H32} plane of the DFT-optimized **2**. Bond critical points (BCPs) are depicted as green spheres, ring critical points (RCPs) are depicted as dark red spheres.

Due to the unusual orientation of the isopropyl group at N3 (i.e. the H of the ^{i}Pr is *anti* to the Zn atom rather than *syn*), a weak bond vector path is observed between Zn1 and H32, a hydrogen of this isopropyl group (BCP ρ = 0.01038).

Again, there is only one bond path observed between Zn and the carborane (CB) cage, to B41, also the central boron atom. This bond path, and that to the carbene carbon (C6) are ionic in nature ($\nabla^2\rho(r)$ values for both BCPs are positive). No other interactions were observed between Zn and the carborane ligand's atoms.

Table S10. Selected QTAIM atomic data for **2**.

Atom	q(A)	L(A)	N(A)	Vol(A)	%Loc(A)
**Zn1**	**+0.717667**	**+0.000346**	**19.282333**	**107.303885**	**93.153392**
N2	-1.049067	-0.000074	8.049067	74.737026	75.869328
N3	-1.058341	-0.000163	8.058341	74.382570	75.914228
**C4**	**-1.067639**	**+0.000240**	**7.067639**	**73.758248**	**68.830730**
**C5**	**-1.065018**	**-0.000279**	**7.065018**	**74.735015**	**68.837798**
**C6**	**+0.606249**	**-0.000059**	**5.393751**	**74.616179**	**67.480629**
C7	+0.333015	-0.000126	5.666985	65.258377	64.425597
C8	-0.011287	-0.000245	6.011287	70.158489	66.403738
C12	+0.333499	+0.000055	5.666501	65.269169	64.420996
C13	-0.016583	+0.000048	6.016583	70.321967	66.404668
C17	+0.288318	+0.000099	5.711682	47.150847	64.545212
C19	-0.024160	+0.000093	6.024160	70.667626	66.434145
C23	-0.027365	-0.000186	6.027365	70.523646	66.425961
C27	+0.301586	+0.000440	5.698414	46.718914	64.404622
C29	-0.031144	-0.000252	6.031144	70.295764	66.484736
H32	+0.046556	+0.000049	0.953444	44.027280	40.317654
C33	-0.031181	+0.000157	6.031181	70.711847	66.502379
**B39**	**+0.749574**	**-0.000284**	**4.250426**	**44.432619**	**62.214507**
**B41**	**+0.352886**	**+0.000060**	**4.647114**	**52.565795**	**61.221327**
**B43**	**+0.752152**	**+0.000197**	**4.247848**	**44.587579**	**62.243802**
B45	+0.789688	+0.000035	4.210312	43.272950	62.315304
B47	+0.934077	+0.000065	4.065923	40.981176	63.089603
B49	+0.793700	-0.000411	4.206300	43.191329	62.318581
B51	+0.535381	-0.000465	4.464619	47.646290	61.216890
B53	+0.538459	+0.000091	4.461541	47.543306	61.220328
B55	+0.551183	-0.000028	4.448817	48.208192	61.283358

Table S11. Selected QTAIM BCP data for **2**.

BCP	ρ(r)	$\nabla^2\rho(r)$	ε	G(r)	V(r)
**Zn1 - C6**	**0.106572**	**+0.222879**	**0.040740**	**+0.098260**	**-0.140800**
C4 - B43	0.130747	+0.017230	0.729956	+0.116672	-0.229036
C5 - B39	0.130532	+0.015708	0.744450	+0.116046	-0.228165
C4 - C5	0.205859	-0.302413	0.330421	+0.068266	-0.212136
**Zn1 - H32**	**0.010378**	**+0.025718**	**0.633827**	**+0.005776**	**-0.005123**
C29 - H32	0.266717	-0.854180	0.011468	+0.039364	-0.292274
B39 - B41	0.107916	-0.091362	3.666892	+0.029114	-0.081068
B39 - B49	0.109682	-0.074248	6.541676	+0.036689	-0.091940
**Zn1 - B41**	**0.082499**	**+0.036119**	**1.074230**	**+0.042602**	**-0.076175**
B41 - B43	0.108291	-0.093992	3.375994	+0.029025	-0.081547
B41 - B53	0.113616	-0.104824	3.029283	+0.036172	-0.098550
B41 - B51	0.113869	-0.106059	2.947887	+0.036145	-0.098805
B39 - B51	0.115280	-0.111171	3.276276	+0.035913	-0.099620
B43 - B45	0.109876	-0.074575	6.575006	+0.036587	-0.091818
B43 - B53	0.115337	-0.111045	3.312756	+0.036010	-0.099781
C4 - B45	0.121186	+0.044844	2.779311	+0.110075	-0.208940
B45 - B47	0.115910	-0.102722	4.185601	+0.038465	-0.102611
C4 - B47	0.118697	-0.078647	2.664748	+0.070563	-0.160787
B45 - B53	0.117363	-0.119178	3.000011	+0.036216	-0.102226
C5 - B47	0.118617	-0.080576	2.684040	+0.069532	-0.159208
C5 - B49	0.121453	+0.043531	2.728245	+0.110084	-0.209285
B47 - B49	0.116022	-0.103327	4.163152	+0.038503	-0.102837
B49 - B51	0.117049	-0.118120	3.025001	+0.036106	-0.101741
B47 - B55	0.119436	-0.122050	3.338464	+0.039174	-0.108861
B53 - B55	0.112717	-0.100362	3.738701	+0.035354	-0.095799
B49 - B55	0.116569	-0.111181	3.714417	+0.037213	-0.102222
B51 - B53	0.110744	-0.095128	4.133679	+0.034410	-0.092603
B45 - B55	0.116686	-0.111627	3.683732	+0.037260	-0.102427
B51 - B55	0.112742	-0.100743	3.694552	+0.035316	-0.095819

[(Cb)Zn(IDipp)]; 3

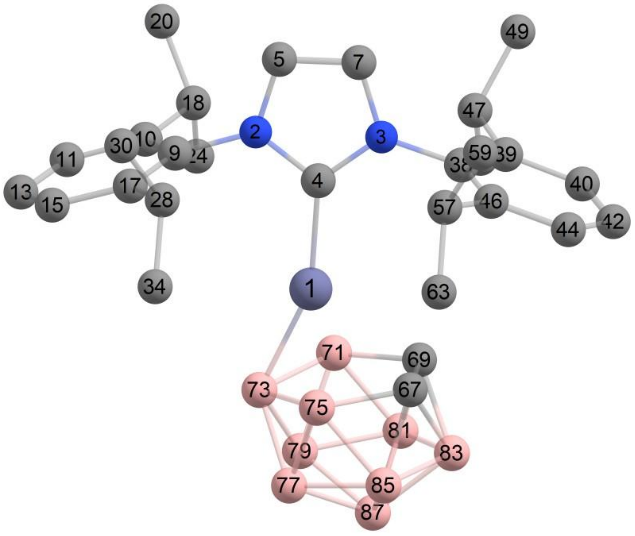

Figure S5: DFT-optimised structure of **3** with associated atom labels. Hydrogen atoms are omitted for clarity. Atom types are depicted by colour: carbon atoms in grey; nitrogen atoms in blue; boron atoms in pink; zinc atoms in glaucous (grey-blue-green).

NBO Charges

Table S12: Natural Population Analysis (NPA) charges of selected atoms in **3**.

Atom	NPA Charge
Zn1	1.48510
C4	−0.00401
C67	−0.62509
C69	−0.62883
B71	−0.28489
B73	−0.49970
B75	−0.27923

∑ = −2.31774

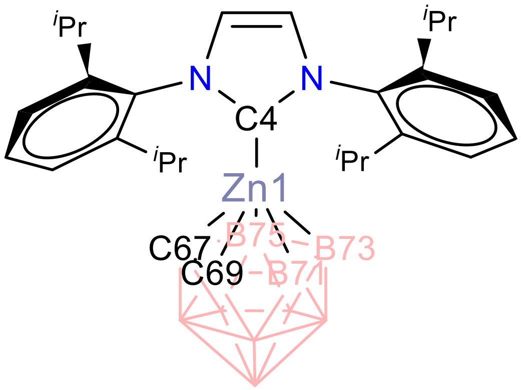

Wiberg Bond Indices

Table S13: Wiberg Bond Indices (WBI) of selected bonds in **3**.

Bond	WBI
Zn1–C4	0.4051
Zn1–C67	0.0138
Zn1–C69	0.0138
Zn1–B71	0.1013
Zn1–B73	0.1781
Zn1–B75	0.1020

∑ = 0.4090

Second Order Perturbation Energies

Again, there are no NBOs involving Zn and C4 or the 5 donor carborane atoms. However, some donor interactions from the ligand atoms to the vacant lone pair of Zn (NBO-151) are observed.

Table S14: Selected donor-acceptor interactions in **3**.

Donor NBO		Acceptor NBO	$\Delta E^{(2)}$ [in kcal/mol]
NBO-51	LP C4	NBO-151 LV Zn1	144.47
NBO-129	BD C67-B75	NBO-151 LV Zn1	9.32
NBO-132	BD C69-B71	NBO-151 LV Zn1	8.88
NBO-135	BD B71-B73	NBO-151 LV Zn1	62.65
NBO-137	BD B73-B75	NBO-151 LV Zn1	60.83

Note: Again, some interactions were also present between the other bonds of the carborane cage, but these were negligible / not significant.

Laplacian ($\nabla^2\rho$) Contour Plot

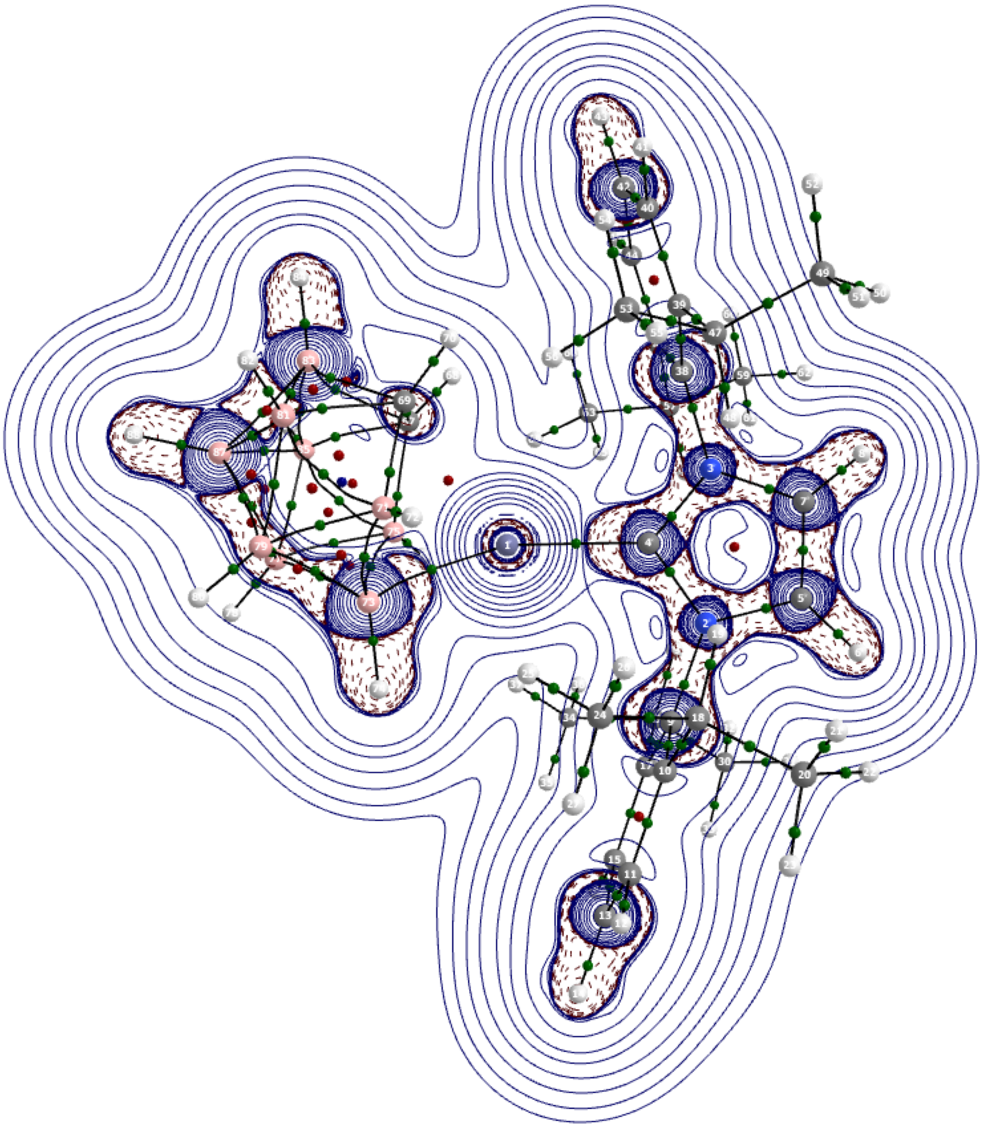

Figure S6: Contour plot of the Laplacian ($\nabla^2\rho(r)$) in the {C4-Zn1-B73} plane of the DFT-optimized **3**. Bond critical points (BCPs) are depicted as green spheres, ring critical points (RCPs) are depicted as dark red spheres.

Table S15. Selected QTAIM atomic data for **3**.

Atom	q(A)	L(A)	N(A)	Vol(A)	%Loc(A)
**Zn1**	**0.733813**	**0.000102**	**19.26619**	**118.1861**	**93.22382**
N2	-1.08684	0.00036	8.086836	65.41707	75.5249
N3	-1.08953	-4.8E-05	8.089527	65.6864	75.54715
**C4**	**0.61144**	**-5.9E-05**	**5.38856**	**74.85644**	**67.5723**
C5	0.322247	-0.00011	5.677753	81.01842	65.56208
C7	0.31826	-4.1E-05	5.68174	80.6128	65.57029
C9	0.239566	-0.00017	5.760434	66.6216	64.81401
C10	0.004667	-0.00015	5.995333	67.34491	64.70591
C11	-0.02496	0.000022	6.02496	81.68156	65.7675
C13	-0.01432	-2.2E-05	6.014317	83.85096	65.84232
C15	-0.02499	-1.5E-05	6.024986	81.72087	65.76748
C17	0.004709	-5.4E-05	5.995291	67.36369	64.70598
C18	0.052701	-6.4E-05	5.947299	50.1946	64.43659
C20	-0.02364	-0.00047	6.023639	70.09574	66.30214
C24	-0.02958	-0.00015	6.029583	70.32811	66.39307
C28	0.053083	0.000438	5.946917	50.22935	64.43844
C30	-0.02354	-0.00036	6.023543	70.02579	66.30231
C34	-0.02947	-3.2E-05	6.029465	70.2665	66.39292
C38	0.247474	0.000049	5.752526	65.36879	64.76768
C39	0.001327	-0.00024	5.998673	65.33886	64.68981
C40	-0.03053	-0.00016	6.030526	82.25927	65.75679
C42	-0.01645	0.000055	6.016448	84.19813	65.84339
C44	-0.03047	-8.7E-05	6.030474	82.31624	65.75716
C46	0.001464	-7.2E-05	5.998536	65.39185	64.69051
C47	0.052218	-0.00019	5.947782	50.333	64.44663
C49	-0.02316	-2.5E-05	6.02316	69.80789	66.30404
C53	-0.02912	-1.6E-05	6.029121	70.16003	66.3755
C57	0.052249	-0.00006	5.947751	50.34352	64.44804
C59	-0.02343	-0.00029	6.023428	69.96504	66.3045
C63	-0.02896	0.000124	6.028958	70.12958	66.37508
**C67**	**-1.0713**	**0.00006**	**7.071304**	**74.39875**	**68.8344**
**C69**	**-1.07146**	**-4.4E-05**	**7.071456**	**74.45529**	**68.83603**
**B71**	**0.756956**	**-0.00035**	**4.243044**	**45.34231**	**62.24014**
**B73**	**0.346869**	**-5.6E-05**	**4.653131**	**52.90454**	**61.2097**
**B75**	**0.7572**	**0.000081**	**4.2428**	**45.33433**	**62.24317**
B77	0.534528	0.000032	4.465472	47.6311	61.21567
B79	0.534846	0.000065	4.465154	47.63354	61.21618
B81	0.794925	-0.00022	4.205075	43.23583	62.32118
B83	0.936389	0.000262	4.063611	40.86584	63.09434
B85	0.795406	0.000148	4.204594	43.22421	62.32327
B87	0.54958	0.000196	4.45042	48.14705	61.28339

Table S16. Selected QTAIM BCP data for **3**.

BCP	ρ(r)	$\nabla^2\rho(r)$	ε	G(r)	V(r)
**Zn1 - C4**	**0.107285**	**0.234039**	**0.079161**	**0.101255**	**-0.144**
C5 - C7	0.319334	-0.87141	0.333575	0.119547	-0.45694
N2 - C4	0.306117	-0.74292	0.135598	0.237168	-0.66007
N2 - C5	0.292079	-0.71413	0.164733	0.201816	-0.58216
N3 - C4	0.305977	-0.73971	0.134803	0.237932	-0.66079
N3 - C7	0.291196	-0.70863	0.166242	0.201587	-0.58033
N2 - C9	0.253581	-0.61071	0.007533	0.146706	-0.44609
C9 - C10	0.290882	-0.73093	0.242277	0.096675	-0.37608
C10 - C11	0.296587	-0.77091	0.189324	0.097993	-0.38871
C11 - C13	0.301005	-0.79979	0.189649	0.099609	-0.39916
C9 - C17	0.290899	-0.73102	0.242311	0.096686	-0.37613
C13 - C15	0.301014	-0.79984	0.189666	0.099616	-0.39919
C15 - C17	0.296574	-0.77084	0.189318	0.097985	-0.38868
C10 - C18	0.238961	-0.51486	0.023737	0.058398	-0.24551
C18 - C20	0.228954	-0.46803	0.010967	0.054884	-0.22678
C18 - C24	0.22969	-0.47169	0.009647	0.055188	-0.2283
C17 - C28	0.238955	-0.51483	0.023737	0.058394	-0.2455
C28 - C30	0.228957	-0.46804	0.010969	0.054886	-0.22678
C28 - C34	0.2297	-0.47173	0.009655	0.05519	-0.22831
N3 - C38	0.255482	-0.62203	0.00482	0.146832	-0.44917
C38 - C39	0.290855	-0.73065	0.241877	0.09654	-0.37574
C39 - C40	0.296149	-0.76847	0.189083	0.097733	-0.38758
C40 - C42	0.300487	-0.79652	0.189298	0.099368	-0.39787
C38 - C46	0.290849	-0.73062	0.241887	0.096537	-0.37573
C42 - C44	0.300487	-0.79652	0.189301	0.099368	-0.39787
C44 - C46	0.296145	-0.76844	0.189088	0.097731	-0.38757
C39 - C47	0.238258	-0.51144	0.023978	0.058195	-0.24425
C47 - C49	0.228431	-0.46551	0.01153	0.054825	-0.22603
C47 - C53	0.230101	-0.47361	0.009339	0.055185	-0.22877
C57 - C59	0.228429	-0.46549	0.011529	0.054824	-0.22602
C57 - C63	0.230104	-0.47363	0.009349	0.055185	-0.22878
C67 - B75	0.130621	0.012913	0.728716	0.115507	-0.22779
C69 - B71	0.130657	0.013007	0.727019	0.115575	-0.2279
C67 - C69	0.207445	-0.30856	0.327271	0.068988	-0.21512
B71 - B79	0.114677	-0.10887	3.362568	0.035715	-0.09865
C69 - B83	0.119029	-0.07856	2.714659	0.071064	-0.16177
C69 - B81	0.121842	0.040882	2.670786	0.109898	-0.20958
B71 - B81	0.109222	-0.07285	6.710503	0.03678	-0.09177
**Zn1 - B73**	**0.08277**	**0.036927**	**1.097828**	**0.043002**	**-0.07677**
B73 - B77	0.114367	-0.10776	2.900426	0.036327	-0.09959
B77 - B85	0.117121	-0.11897	2.956586	0.036064	-0.10187
B79 - B81	0.117146	-0.11907	2.952799	0.036069	-0.10191
B71 - B73	0.108031	-0.09238	3.548038	0.02911	-0.08132
B73 - B79	0.114361	-0.10772	2.90479	0.036333	-0.09959
B75 - B77	0.114676	-0.1089	3.358033	0.03571	-0.09864
C67 - B85	0.121858	0.040928	2.665731	0.10993	-0.20963
C67 - B83	0.119016	-0.07852	2.717668	0.071053	-0.16174
B73 - B75	0.107984	-0.09213	3.572445	0.029109	-0.08125
B75 - B85	0.109215	-0.07285	6.697697	0.036785	-0.09178
B77 - B79	0.110882	-0.09463	4.306052	0.03459	-0.09284
B81 - B83	0.116012	-0.10295	4.282181	0.038662	-0.10306
B79 - B87	0.112928	-0.1013	3.662333	0.035379	-0.09608
B83 - B87	0.119486	-0.12264	3.258769	0.039017	-0.1087
B83 - B85	0.116009	-0.10294	4.283879	0.038659	-0.10305
B85 - B87	0.116396	-0.11037	3.793122	0.037206	-0.102
B81 - B87	0.116392	-0.11034	3.795114	0.037206	-0.102
B77 - B87	0.112929	-0.10132	3.660469	0.035377	-0.09608

$[Zn(Cb)_2]^-$ anion from **4** and **5**

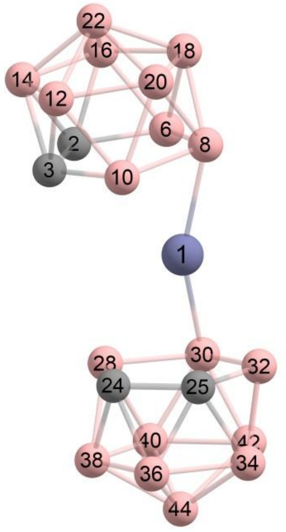

Figure S7: DFT-optimised structure of the bis-carboranyl zinc anion of **4**/**5**. with associated atom labels. Hydrogen atoms are omitted for clarity. Atom types are depicted by colour: carbon atoms in grey; boron atoms in pink; zinc atoms in glaucous (grey-blue-green).

NBO Charges

Table S17: Natural Population Analysis (NPA) charges of selected atoms for the bis-carboranyl zinc anion of **4**/**5**.

Atom	NPA Charge		Atom	NPA Charge	
Zn1	1.57753				
C2	−0.58187		C24	−0.58538	
C3	−0.58539		C25	−0.58189	
B6	−0.18410	Σ = −2.12464	B28	−0.19489	Σ = −2.12465
B8	−0.57817		B30	−0.57820	
B10	−0.19511		B32	−0.18429	

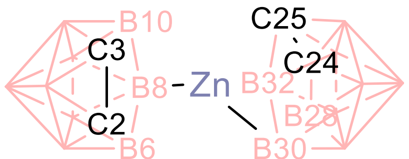

Wiberg Bond Indices

Table S18: Wiberg Bond Indices of selected bonds in the bis-carbornanyl zinc anion of **4**/**5**.

Bond	WBI		Bond	WBI	
Zn1–C2	0.0074	Σ = 0.3533	Zn1–C24	0.0080	Σ = 0.3533
Zn1–C3	0.0080		Zn1–C25	0.0074	
Zn1–B6	0.0723		Zn1–B28	0.0813	
Zn1–B8	0.1842		Zn1–B30	0.1842	
Zn1–B10	0.0814		Zn1–B32	0.0724	

Both NBO charges and WBIs in this bis-carboranyl structure are equivalent to the Zn-CB bonding observed in the previous mono-carborane/carbene structures.

Second Order Perturbation Energies

Again, there are no NBOs involving Zn and the 5 donor carborane atoms. However, some donor interactions from the B–B bonds to the vacant lone pair of Zn (NBO-80) are observed.

Table S19: Selected donor-acceptor interactions within the bis-carboranyl zinc anion of **4**/**5**.

Donor NBO		Acceptor NBO	$\Delta E^{(2)}$ [in kcal/mol]
NBO-42	BD B6-B8	NBO-80 LV Zn1	47.09
NBO-44	BD B8-B10	NBO-80 LV Zn1	41.78
NBO-60	BD B28-B30	NBO-80 LV Zn1	41.74
NBO-62	BD B30-B32	NBO-80 LV Zn1	47.13

Note: Some interactions were also present between the other three bonds of each carborane cage, but these were negligible / not significant.

In comparison to **1** and **2**, here the C–B bond donation into the Zn LV falls below the reporting threshold of 10 kcal/mol. The B–B bond donation $\Delta E^{(2)}$ values are 20 kcal/mol lower than those seen in the data for **1** and **2**.

Laplacian ($\nabla^2\rho$) Contour Plot

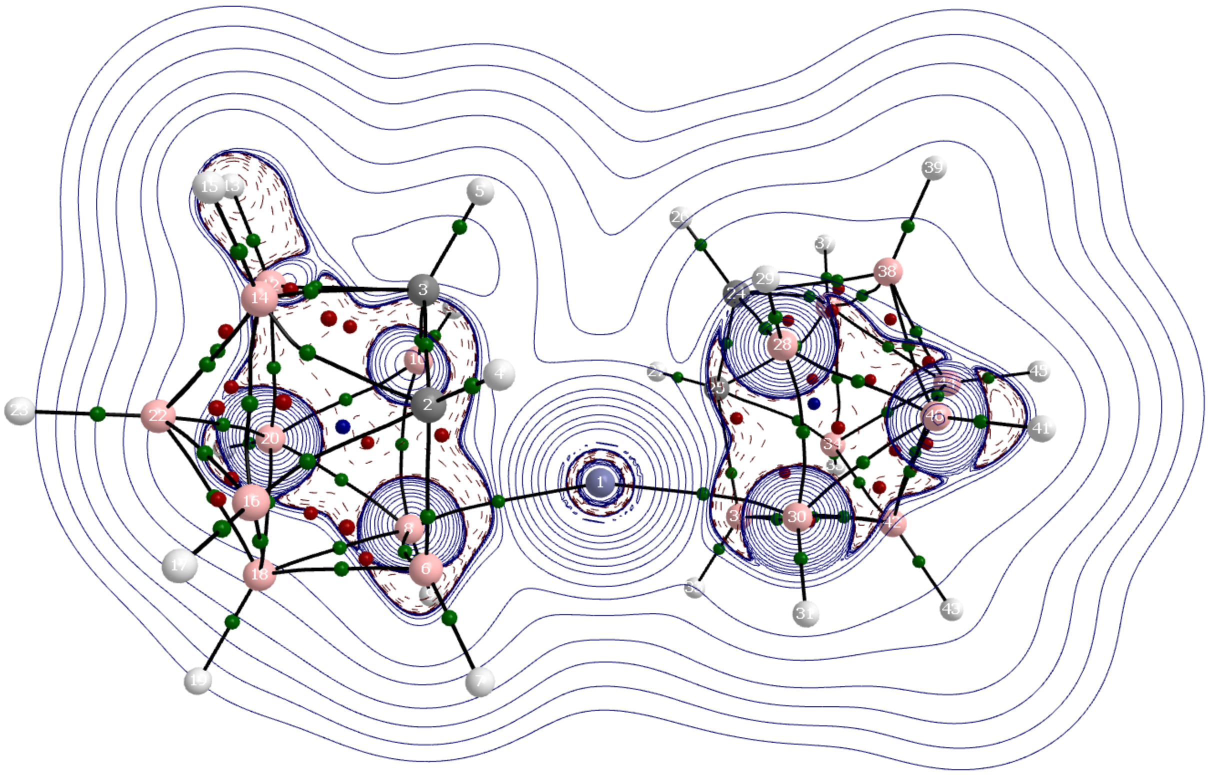

Figure S8: Contour plot of the Laplacian ($\nabla^2\rho(r)$) in the {B20-Zn1-B30} plane of the DFT-optimized anion from **4** and **5**. Bond critical points (BCPs) are depicted as green spheres, ring critical points (RCPs) are depicted as dark red spheres.

As with the preceding neutral complexes, **1** and **2**, only one bond path is observed between Zn and each carborane (CB) cage, to B8 and B30, both the central boron atom. No other interactions were observed between Zn and the carborane ligand atoms, and the two bond paths located are ionic in nature ($\nabla^2\rho(r)$ values for both BCPs are positive).

Table S20. Selected QTAIM atomic data for anion of **4**/**5**.

Atom	q(A)	L(A)	N(A)	Vol(A)	%Loc(A)
**Zn1**	**0.731393**	**0.000453**	**19.26861**	**104.4746**	**93.46021**
**C2**	**-1.06103**	**0.000104**	**7.061028**	**76.26512**	**69.07016**
**C3**	**-1.06818**	**-0.00023**	**7.068179**	**75.06885**	**69.04915**
**B6**	**0.871849**	**-0.0003**	**4.128151**	**43.6484**	**62.53463**
**B8**	**0.288465**	**0.000379**	**4.711535**	**52.37467**	**61.25401**
**B10**	**0.86331**	**0.000306**	**4.13669**	**43.09309**	**62.5031**
B12	0.764965	0.000253	4.235035	44.36053	62.17315
B14	0.926717	-6.3E-05	4.073283	41.30474	62.84628
B16	0.758091	-0.00035	4.241909	44.61472	62.15232
B18	0.486325	-0.00035	4.513675	48.96727	61.19023
B20	0.429235	-0.00585	4.570685	50.31777	60.50893
B22	0.494347	-0.00039	4.505653	50.10725	61.22195
**C24**	**-1.0678**	**-0.00011**	**7.067804**	**75.02643**	**69.05027**
**C25**	**-1.06094**	**0.000226**	**7.060935**	**76.26425**	**69.0702**
**B28**	**0.863867**	**0.000443**	**4.136133**	**43.12509**	**62.50646**
**B30**	**0.286835**	**-0.00037**	**4.713165**	**52.44749**	**61.25165**
**B32**	**0.871818**	**-0.00022**	**4.128182**	**43.71928**	**62.53475**
B34	0.758455	-7.9E-05	4.241545	44.59286	62.15424
B36	0.926218	-0.00046	4.073782	41.37306	62.84308
B38	0.7648	0.000094	4.2352	44.39879	62.17229
B40	0.482891	-0.00002	4.517109	48.97373	61.19095
B42	0.486407	-0.00016	4.513593	48.94154	61.19099
B44	0.494841	-4.8E-05	4.505159	50.03291	61.22328

Table S21. Selected QTAIM BCP data for anion of **4**/**5**.

BCP	ρ(r)	$\nabla^2\rho(r)$	ε	G(r)	V(r)
C2 - B6	0.140937	0.011129	0.395462	0.128304	-0.25383
C2 - C3	0.213704	-0.34064	0.284917	0.069624	-0.22441
**Zn1 - B8**	**0.074993**	**0.048346**	**0.48803**	**0.039926**	**-0.06777**
C2 - B14	0.120932	-0.08488	2.62147	0.072415	-0.16605
C2 - B16	0.122357	0.009005	2.793611	0.102697	-0.20314
C3 - B10	0.140788	0.006898	0.4	0.127171	-0.25262
B6 - B8	0.111846	-0.11442	2.291017	0.028728	-0.08606
B6 - B18	0.114493	-0.10634	3.610949	0.036071	-0.09873
B8 - B10	0.111143	-0.11036	2.52657	0.028918	-0.08543
C3 - B12	0.12285	0.008598	2.673671	0.103221	-0.20429
B8 - B20	0.113611	-0.10421	3.074197	0.035943	-0.09794
B16 - B22	0.117116	-0.11205	3.466915	0.037171	-0.10235
B12 - B20	0.119059	-0.12734	2.449849	0.03591	-0.10366
B12 - B14	0.116359	-0.10204	4.646335	0.039433	-0.10438
C3 - B14	0.121302	-0.08422	2.602133	0.073306	-0.16767
B16 - B18	0.119571	-0.12879	2.44039	0.036065	-0.10433
B14 - B22	0.11963	-0.12071	3.309225	0.038485	-0.10715
B14 - B16	0.116178	-0.10218	4.482888	0.03923	-0.104
B8 - B18	0.113125	-0.10169	3.282488	0.035976	-0.09738
B10 - B20	0.114378	-0.10665	3.52418	0.035812	-0.09829
B20 - B22	0.111496	-0.09466	4.115715	0.035138	-0.09394
B18 - B20	0.109433	-0.0915	3.865763	0.033813	-0.0905
B12 - B22	0.11737	-0.11308	3.408701	0.03725	-0.10277
B18 - B22	0.111746	-0.09544	4.030219	0.035235	-0.09433
C25 - B32	0.140939	0.011115	0.395543	0.128303	-0.25383
C24 - C25	0.2137	-0.34063	0.28492	0.069622	-0.2244
**Zn1 - B30**	**0.074994**	**0.048346**	**0.487711**	**0.039927**	**-0.06777**
B28 - B30	0.111154	-0.11042	2.523752	0.028918	-0.08544
C24 - B28	0.14079	0.006925	0.399826	0.127179	-0.25263
C24 - B38	0.122848	0.008594	2.674255	0.103216	-0.20428
C25 - B34	0.122359	0.009016	2.792741	0.102702	-0.20315
B30 - B40	0.11361	-0.1042	3.075568	0.035944	-0.09794
B30 - B32	0.111836	-0.11438	2.293105	0.028729	-0.08605
B32 - B42	0.114495	-0.10636	3.609467	0.03607	-0.09873
C25 - B36	0.120931	-0.08487	2.621624	0.07242	-0.16606
B34 - B44	0.117117	-0.11205	3.466312	0.037171	-0.10236
B34 - B42	0.119567	-0.12878	2.440786	0.036065	-0.10432
C24 - B36	0.121301	-0.08423	2.601885	0.0733	-0.16766
B34 - B36	0.116177	-0.10217	4.483672	0.039229	-0.104
B36 - B38	0.116358	-0.10205	4.645619	0.039433	-0.10438
B38 - B40	0.119064	-0.12736	2.449235	0.035911	-0.10366
B36 - B44	0.119629	-0.12071	3.309084	0.038485	-0.10715
B28 - B40	0.114378	-0.10664	3.5254	0.035814	-0.09829
B42 - B44	0.111746	-0.09544	4.030478	0.035235	-0.09433
B38 - B44	0.117366	-0.11306	3.409454	0.037249	-0.10276
B40 - B42	0.109434	-0.0915	3.865243	0.033813	-0.0905
B30 - B42	0.113124	-0.10169	3.281729	0.035974	-0.09737
B40 - B44	0.111496	-0.09466	4.115459	0.035138	-0.09394

[(Cb)Zn·Py]$_2$; **6**

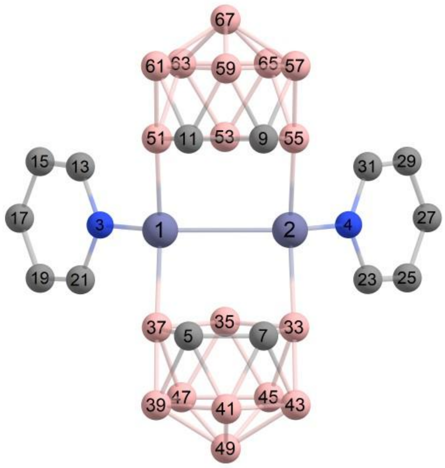

Figure S9: DFT-optimised structure of **6** with associated atom labels. Hydrogen atoms are omitted for clarity. Atom types are depicted by colour: carbon atoms in grey; nitrogen atoms in blue; boron atoms in pink; zinc atoms in glaucous (grey-blue-green).

‘Bottom’ carborane cage = CB1 = C5, C7, B33, B35, B37

‘Top’ carborane cage = CB2 = C9, C11, B51, B53, B55

NBO Charges

Table S22: Natural Population Analysis (NPA) charges of selected atoms in **6**.

Atom	NPA Charge		Atom	NPA Charge	
Zn1	1.60307		Zn2	1.59856	
N3	−0.58664		N4	−0.58707	
C5	−0.54167	Σ = −2.50162	C9	−0.54150	Σ = −2.52889
C7	−0.55340		C11	−0.54626	
B33	−0.36676		B51	−0.40421	
B35	−0.63483		B53	−0.63230	
B37	−0.40496		B55	−0.40462	

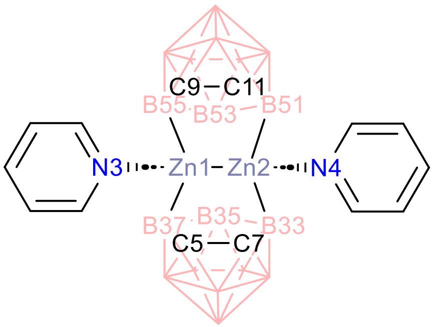

Wiberg Bond Indices

Table S23: Wiberg Bond Indices (WBI) of selected bonds in **6**.

Bond	WBI		Bond	WBI	
Zn1–Zn2	0.0112				
Zn1–N3	0.1482		Zn2–N4	0.1485	
Zn1–C5	0.0073		Zn2–C5	0.0051	
Zn1–C7	0.0051		Zn2–C7	0.0090	
Zn1–B33	0.0046	Σ = 0.2393	Zn2–B33	0.1279	Σ = 0.2448
Zn1–B35	0.0976		Zn2–B35	0.0980	
Zn1–B37	0.1247		Zn2–B37	0.0048	
Zn2–C9	0.0073		Zn1–C9	0.0051	
Zn2–C11	0.0051		Zn1–C11	0.0073	
Zn2–B51	0.0047	Σ = 0.2395	Zn1–B51	0.1246	Σ = 0.2394
Zn2–B53	0.0977		Zn1–B53	0.0976	
Zn2–B55	0.1247		Zn1–B55	0.0048	

Second Order Perturbation Energies

There are no NBOs involving Zn1 or Zn2 with the carborane atoms (or each other). However, there are many donor-acceptor interactions ($\Delta E^{(2)}$) between the bonds of the carborane cages and both Zn atoms, only significant (over 10 kcal/mol) values are given in the table below where the accepting NBO is either the vacant lone pair (LV) of Zn or the first Rydberg NBO of the Zn (NBO-223 for Zn1 and NBO-301 for Zn2).

Table S24: Selected donor-acceptor interactions of **6**.

Donor NBO		Acceptor NBO	$\Delta E^{(2)}$ [in kcal/mol]
NBO-53	LP N3	NBO-223 RY Zn1	30.13
NBO-54	LP N4	NBO-301 RY Zn2	32.02
NBO-100	BD B33-B35	NBO-301 RY Zn2	79.10
NBO-102	BD B35-B37	NBO-223 RY Zn1	74.51
NBO-111	BD B51-B53	NBO-223 RY Zn1	76.07
NBO-113	BD B53-B55	NBO-132 LV Zn2	11.19
NBO-113	BD B53-B55	NBO-301 RY Zn2	79.90
NBO-114	BD B55-H56	NBO-301 RY Zn2	27.19

Laplacian ($\nabla^2\rho$) Contour Plots

There are two bond vectors to each Zn atom, one from each CB cage, the BCPs are Zn1-B37 (CB1), Zn1-B51 (CB2), Zn2-B33 (CB1) and Zn2-B55 (CB2). These are the four carbon-adjacent boron atoms.

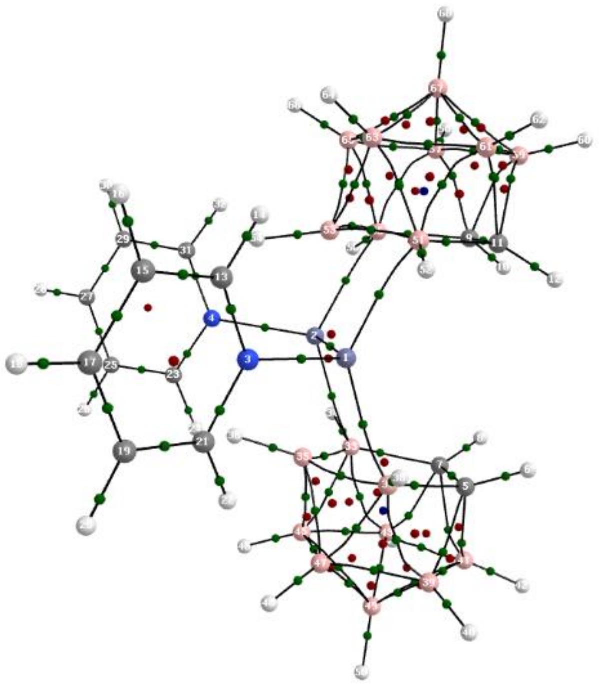

Figure S10: Side view of the DFT-optimized **6**, with bond paths and critical points shown. Bond critical points (BCPs) are depicted as green spheres, ring critical points (RCPs) are depicted as dark red spheres.

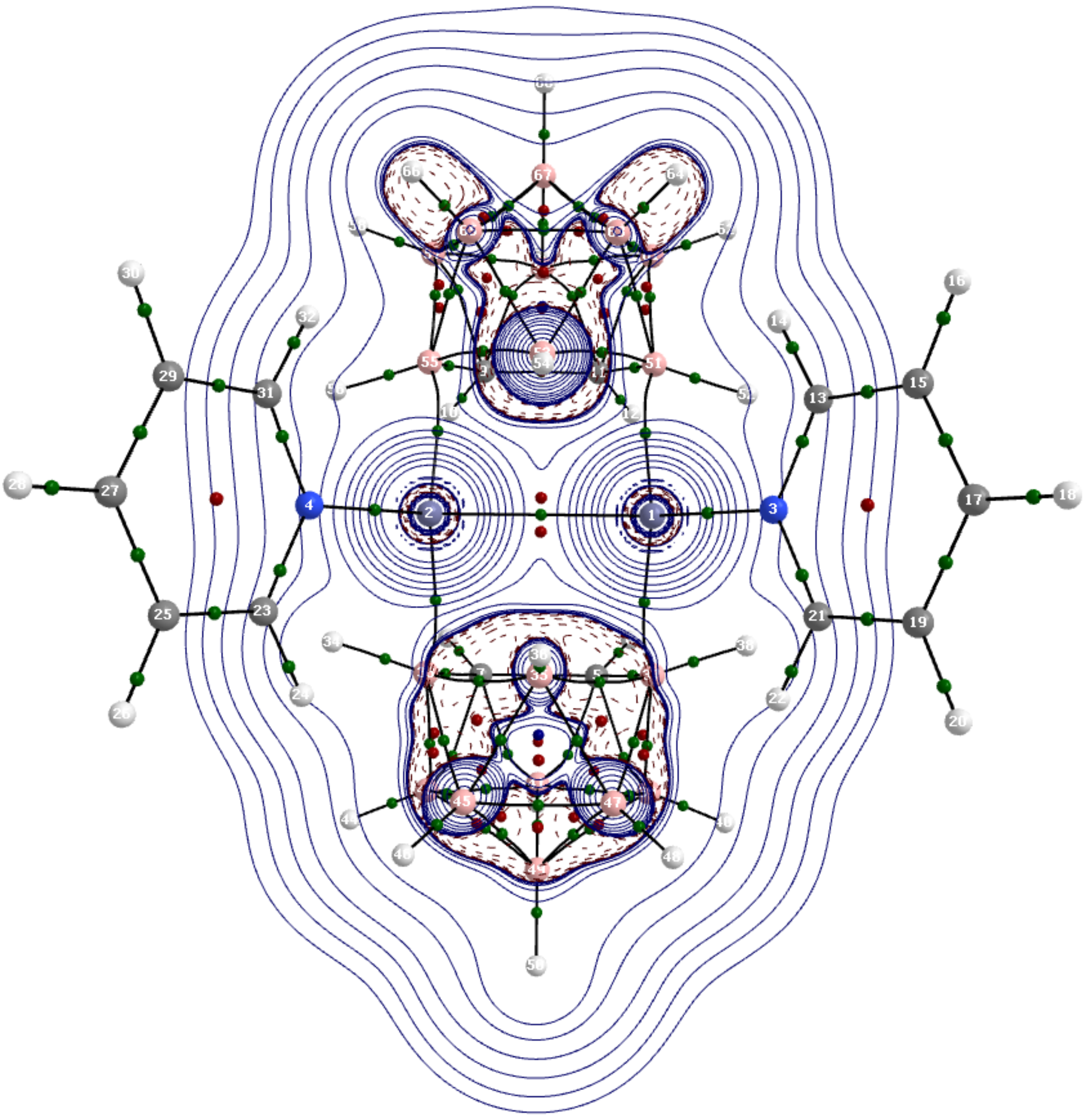

Figure S11: Contour plot of the Laplacian ($\nabla^2\rho(r)$) in the {Zn1-Zn2-B53} plane of the DFT-optimized **6**. Bond critical points (BCPs) are depicted as green spheres, ring critical points (RCPs) are depicted as dark red spheres.

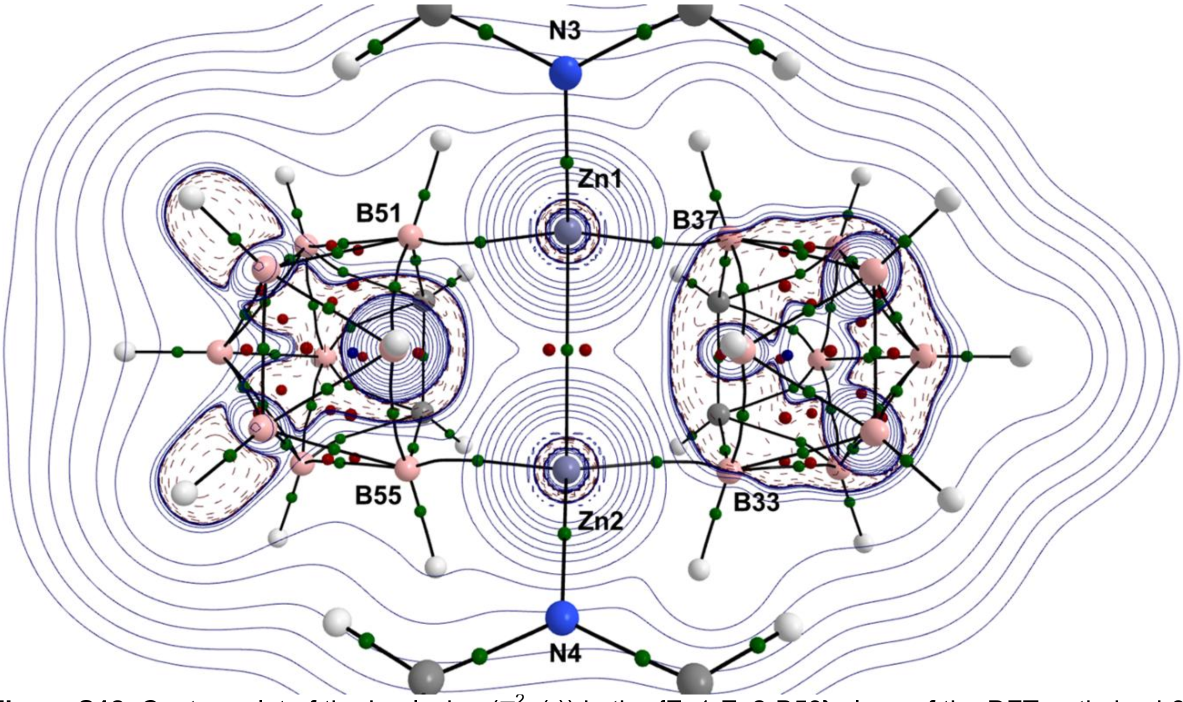

Figure S12: Contour plot of the Laplacian ($\nabla^2\rho(r)$) in the {Zn1-Zn2-B53} plane of the DFT-optimized **6**, zoomed in on the Zn–Zn bond and carborane cages. Bond critical points (BCPs) are depicted as green spheres, ring critical points (RCPs) are depicted as dark red spheres.

Table S25. Selected QTAIM atomic data for **6**.

Atom	q(A)	L(A)	N(A)	Vol(A)	%Loc(A)
**Zn1**	**0.832366**	**-0.001673**	**19.167634**	**89.466518**	**93.347855**
**Zn2**	**0.833906**	**-0.000184**	**19.166094**	**88.866979**	**93.356791**
N3	-1.106694	0.000138	8.106694	92.739995	77.976333
N4	-1.10684	-0.000002	8.10684	92.767308	77.975576
**C5**	**-1.045604**	**0.00007**	**7.045604**	**78.484998**	**69.150957**
**C7**	**-1.045475**	**0.000027**	**7.045475**	**78.535613**	**69.15104**
**C9**	**-1.045597**	**-0.000073**	**7.045597**	**78.555804**	**69.152075**
**C11**	**-1.045629**	**0.00003**	**7.045629**	**78.499902**	**69.15189**
C13	0.434841	-0.000086	5.565159	72.43887	65.093988
C15	-0.016937	0.000024	6.016937	84.660058	66.048711
C17	-0.009336	0.000048	6.009336	83.582296	65.948968
C19	-0.016945	0.000008	6.016945	84.731602	66.048506
C21	0.435016	0.000125	5.564984	72.383002	65.093964
C23	0.434914	0.000005	5.565086	72.370864	65.09404
C25	-0.016878	0.000091	6.016878	84.689593	66.04859
C27	-0.009526	-0.000162	6.009526	83.590249	65.949564
C29	-0.017094	-0.000142	6.017094	84.769628	66.049103
C31	0.434943	0.000065	5.565057	72.411023	65.09436
**B33**	**0.701035**	**-0.000369**	**4.298965**	**41.448162**	**61.991615**
**B35**	**0.287649**	**0.000081**	**4.712351**	**47.341324**	**61.056475**
**B37**	**0.702407**	**0.000464**	**4.297593**	**41.385007**	**61.998478**
B39	0.818129	-0.00009	4.181871	42.888594	62.294838
B41	0.988662	-0.000068	4.011338	39.994858	63.109994
B43	0.818014	-0.00005	4.181986	42.885355	62.294976
B45	0.518501	0.000051	4.481499	47.189166	61.213361
B47	0.518178	-0.000167	4.481822	47.295874	61.212166
B49	0.557432	0.000114	4.442568	48.323769	61.269458
**B51**	**0.702266**	**0.000483**	**4.297734**	**41.262296**	**61.998562**
**B53**	**0.287218**	**-0.000107**	**4.712782**	**47.38188**	**61.055166**
**B55**	**0.702808**	**0.00041**	**4.297192**	**41.301958**	**61.998581**
B57	0.818153	0.000108	4.181847	42.833616	62.295514
B59	0.988828	0.000011	4.011172	39.972829	63.11025
B61	0.818119	-0.000081	4.181881	42.799888	62.294654
B63	0.518695	-0.000092	4.481305	47.319039	61.213403
B65	0.518556	0.000098	4.481444	47.258972	61.214294
B67	0.55735	-0.000035	4.44265	48.347873	61.26896

Table S26. Selected QTAIM BCP data for **6**.

BCP	ρ(r)	∇^2ρ(r)	ε	G(r)	V(r)
**Zn1 - Zn2**	**0.030058**	**0.049068**	**12.496165**	**0.016669**	**-0.02107**
Zn1 - N3	0.073362	0.241039	0.032022	0.078433	-0.096607
Zn2 - N4	0.073376	0.241096	0.032018	0.078455	-0.096635
**Zn1 - B37**	**0.06548**	**0.063477**	**1.573911**	**0.037097**	**-0.058324**
C5 - C7	0.220027	-0.367304	0.284364	0.072319	-0.236464
**Zn2 - B33**	**0.06547**	**0.063527**	**1.575536**	**0.037104**	**-0.058326**
C9 - C11	0.220026	-0.367302	0.284372	0.072319	-0.236463
**Zn2 - B55**	**0.06547**	**0.063483**	**1.578604**	**0.03709**	**-0.058308**
**Zn1 - B51**	**0.065473**	**0.063517**	**1.575484**	**0.037103**	**-0.058326**
C7 - B41	0.121365	-0.071941	2.591971	0.077843	-0.173672
C7 - B43	0.120906	-0.019771	3.129468	0.092734	-0.19041
C7 - B33	0.14728	-0.024602	0.447774	0.127986	-0.262123
C5 - B37	0.147262	-0.024589	0.447983	0.127967	-0.262081
C5 - B39	0.120913	-0.019716	3.127175	0.092759	-0.190446
B33 - B35	0.111529	-0.117292	1.695777	0.028719	-0.086761
B33 - B45	0.115495	-0.111848	2.942201	0.035711	-0.099385
B35 - B37	0.111521	-0.117257	1.697192	0.028718	-0.08675
C5 - B41	0.121364	-0.07194	2.592278	0.077841	-0.173667
B37 - B47	0.115489	-0.111833	2.942166	0.035709	-0.099377
B37 - B39	0.110027	-0.073671	7.270285	0.037712	-0.093842
B39 - B47	0.117188	-0.116183	3.17863	0.036127	-0.101299
B41 - B49	0.116281	-0.109963	3.767869	0.036827	-0.101144
B39 - B41	0.115692	-0.101676	4.956271	0.039137	-0.103693
B33 - B43	0.110033	-0.073686	7.26923	0.03771	-0.093842
B41 - B43	0.115692	-0.101681	4.954436	0.039137	-0.103694
B43 - B45	0.117193	-0.116194	3.1788	0.036129	-0.101307
B45 - B47	0.112166	-0.103442	3.141757	0.033907	-0.093674
B35 - B45	0.107862	-0.081885	4.140716	0.035392	-0.091255
B47 - B49	0.113862	-0.103129	3.75693	0.035937	-0.097655
B43 - B49	0.116678	-0.113092	3.324703	0.037028	-0.102328
B45 - B49	0.113866	-0.103146	3.755639	0.035938	-0.097662
B35 - B47	0.107868	-0.081914	4.137361	0.035392	-0.091262
B39 - B49	0.116679	-0.1131	3.32414	0.037027	-0.102329
C11 - B59	0.121362	-0.071929	2.592449	0.077842	-0.173667
C11 - B61	0.120908	-0.019765	3.128387	0.092738	-0.190417
C11 - B51	0.147281	-0.024612	0.447791	0.127986	-0.262125
C9 - B55	0.147278	-0.024597	0.447688	0.127985	-0.262119
C9 - B57	0.120906	-0.019756	3.129377	0.092737	-0.190414
B51 - B53	0.111526	-0.117281	1.696019	0.028718	-0.086757
B51 - B63	0.115493	-0.111841	2.942156	0.035711	-0.099381
B53 - B55	0.111525	-0.117275	1.696283	0.028719	-0.086756
C9 - B59	0.121368	-0.071934	2.59137	0.07785	-0.173684
B55 - B65	0.115487	-0.111816	2.943126	0.035708	-0.09937
B55 - B57	0.11003	-0.073675	7.271281	0.037709	-0.093836
B57 - B65	0.117196	-0.11621	3.177737	0.036129	-0.10131
B59 - B67	0.11628	-0.109962	3.76776	0.036826	-0.101143
B57 - B59	0.115691	-0.101675	4.955795	0.039137	-0.103693
B51 - B61	0.110032	-0.073686	7.268117	0.037712	-0.093845
B59 - B61	0.11569	-0.101675	4.954993	0.039136	-0.10369
B61 - B63	0.117192	-0.116188	3.178892	0.036128	-0.101304
B63 - B65	0.112164	-0.103434	3.142111	0.033907	-0.093672
B53 - B63	0.107863	-0.081892	4.13957	0.035392	-0.091257
B65 - B67	0.113865	-0.103141	3.756246	0.035938	-0.097661
B61 - B67	0.116679	-0.113097	3.324188	0.037027	-0.102329
B63 - B67	0.113864	-0.103139	3.756135	0.035937	-0.097659
B53 - B65	0.107865	-0.081905	4.137779	0.035391	-0.091258
B57 - B67	0.116677	-0.113088	3.324847	0.037027	-0.102326

[(Cb)Zn(PPh$_3$)$_2$]; **7**

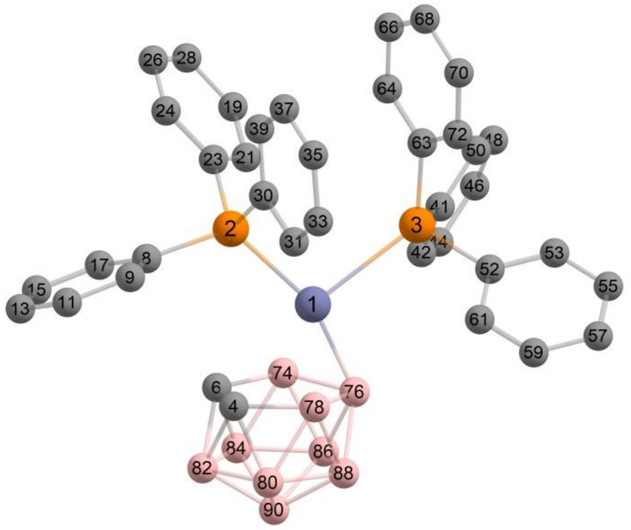

Figure S13: DFT-optimised structure of **7** with associated atom labels. Hydrogen atoms are omitted for clarity. Atom types are depicted by colour: carbon atoms in grey; nitrogen atoms in blue; boron atoms in pink; phosphorus atoms in orange; zinc atoms in glaucous (grey-blue-green).

NBO Charges

Table S27: Natural Population Analysis (NPA) charges of selected atoms in **7**.

Atom	NPA Charge
Zn1	1.34224
P2	0.84272
P3	0.83934
C4	−0.60547
C6	−0.60084
B74	−0.27776
B76	−0.51775
B78	−0.27298

$\Sigma = -2.2748$

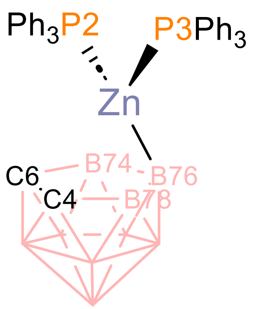

Wiberg Bond Indices

Table S28: Wiberg Bond Indices (WBI) of selected bonds in **7**.

Bond	WBI	
Zn1–P2	0.3276	
Zn1–P3	0.2069	
Zn1–C4	0.0096	$\sum = 0.3670$
Zn1–C6	0.0096	
Zn1–B74	0.0892	
Zn1–B76	0.1720	
Zn1–B78	0.0866	

Second Order Perturbation Energies

Again, there are no NBOs involving Zn and phosphorus atoms or the 5 donor carborane atoms. However, some donor interactions from the ligand atoms to the vacant lone pair of Zn (NBO-183) are observed.

Table S29: Selected donor-acceptor interactions in **7**.

Donor NBO		Acceptor NBO	$\Delta E^{(2)}$ [in kcal/mol]
NBO-67	LP P2	NBO-183 LV Zn1	102.34
NBO-68	LP P3	NBO-183 LV Zn1	57.91
NBO-77	BD C4-B78	NBO-151 LV Zn1	7.19
NBO-80	BD C6-B74	NBO-151 LV Zn1	5.91
NBO-167	BD B74-B76	NBO-151 LV Zn1	57.82
NBO-169	BD B76-B78	NBO-151 LV Zn1	66.68

Note: Again, some interactions were also present between the other bonds of the carborane cage, but these were negligible / not significant.

Laplacian ($\boldsymbol{\nabla}^2\rho$) Contour Plot

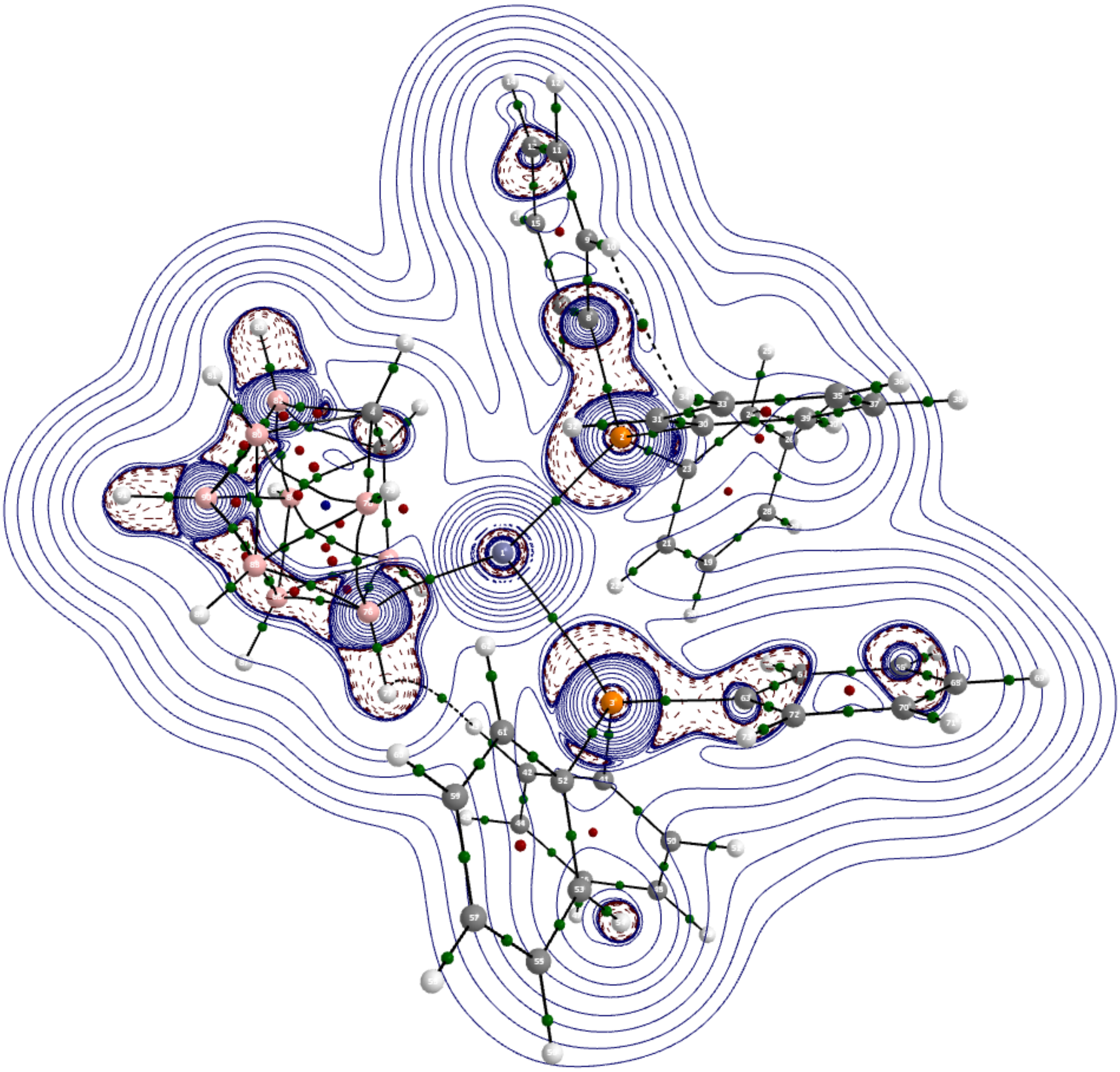

Figure S14: Contour plot of the Laplacian ($\nabla^2\rho(r)$) in the {P2-Zn1-P3} plane of the DFT-optimized **7**. Bond critical points (BCPs) are depicted as green spheres, ring critical points (RCPs) are depicted as dark red spheres.

Table S30. Selected QTAIM atomic data for **7**.

Atom	q(A)	L(A)	N(A)	Vol(A)
**Zn1**	**0.621148**	**-2.2E-05**	**19.37885**	**104.142**
**P2**	**1.378954**	**-9.5E-05**	**13.62105**	**94.46195**
**P3**	**1.31712**	**-0.00019**	**13.68288**	**104.2183**
**C4**	**-1.07605**	**-8.5E-05**	**7.076047**	**73.80997**
H5	0.048641	0.00003	0.951359	47.10267
**C6**	**-1.07454**	**0.000001**	**7.074537**	**74.19087**
H7	0.044219	0.000028	0.955781	46.5197
C8	-0.48832	0.000003	6.48832	77.99402
C9	-0.02546	-4.3E-05	6.025459	82.02807
H10	0.056303	0.000012	0.943697	45.02358
C11	-0.02097	-0.00014	6.02097	84.78187
H12	0.046486	0.000019	0.953514	48.72838
C13	-0.02174	-5.5E-05	6.021741	84.80312
H14	0.046953	0.000016	0.953047	48.77622
C15	-0.01906	-0.00013	6.019059	84.58658
H16	0.048683	0.00002	0.951317	48.64176
C17	-0.02458	-6.8E-05	6.024576	78.71577
H18	0.052492	0.000035	0.947508	47.17169
C19	-0.02414	-0.00014	6.024138	84.98951
H20	0.055747	0.00002	0.944253	48.03138
C21	-0.0264	0.000156	6.026404	79.00595
H22	0.059257	0.000068	0.940743	46.68696
C23	-0.51648	-0.00011	6.516481	79.93506
C24	-0.02711	-0.00018	6.02711	78.91485
H25	0.053498	0.000033	0.946502	47.03068
C26	-0.02509	-8.5E-05	6.025092	84.90692
H27	0.046572	0.000016	0.953428	48.73218
C28	-0.02433	0.000091	6.024334	84.83359
H29	0.047686	0.000021	0.952314	48.74032
C30	-0.51034	0.000405	6.510338	78.92536
C31	-0.04098	0.000167	6.040984	81.81938
H32	0.066654	0.000042	0.933346	46.17032
C33	-0.0233	-6.4E-05	6.023304	85.58768
H34	0.04995	0.000019	0.95005	48.57474
C35	-0.02319	-4.2E-05	6.023186	85.84449
H36	0.04328	0.00002	0.95672	49.06379
C37	-0.01851	-0.00015	6.018507	85.27391
H38	0.043124	0.000025	0.956876	49.4532
C39	-0.01582	0.000006	6.015822	81.69606
H40	0.05075	0.000034	0.94925	45.95591
C41	-0.49571	0.000172	6.495707	80.76277
C42	-0.02938	0.000105	6.029376	83.25754
H43	0.090043	0.000117	0.909957	40.58698
C44	-0.02386	0.000103	6.023857	84.7845
H45	0.053745	0.000018	0.946255	48.25593
C46	-0.02932	0.0001	6.029319	85.08669

H47	0.040751	0.00002	0.959249	49.26236
C48	-0.0311	0.000094	6.031095	85.30301
H49	0.037939	0.000024	0.962061	49.40352
C50	-0.03625	-6.5E-05	6.036253	80.16438
H51	0.049562	0.000184	0.950438	45.63474
C52	-0.48629	0.000229	6.486294	77.26284
C53	-0.02261	-4.9E-05	6.022609	81.95951
H54	0.048204	0.000042	0.951796	46.67097
C55	-0.0266	-0.00014	6.026601	85.14598
H56	0.039507	0.000025	0.960493	49.3002
C57	-0.02619	-2.1E-05	6.026186	84.94501
H58	0.041307	0.000021	0.958693	49.18767
C59	-0.02121	-2.5E-05	6.02121	84.66306
H60	0.049508	0.000019	0.950492	48.52713
C61	-0.01976	0.000081	6.019762	81.39994
H62	0.06711	0.000051	0.93289	45.03313
C63	-0.49042	0.00012	6.490416	79.65349
C64	-0.03267	-7.7E-05	6.032666	79.60265
H65	0.046249	0.000029	0.953751	46.46431
C66	-0.02992	-0.00019	6.029923	85.63262
H67	0.037266	0.000019	0.962734	49.39432
C68	-0.03182	-0.00021	6.031816	86.19056
H69	0.036083	0.000026	0.963917	49.58085
C70	-0.02507	-0.00016	6.025069	85.76257
H71	0.039125	0.000019	0.960875	49.31806
C72	-0.02174	0.000239	6.021737	84.21216
H73	0.058665	0.000007	0.941335	45.54977
**B74**	**0.803581**	**-1.9E-05**	**4.196419**	**43.922**
H75	-0.57598	0.000017	1.575981	82.56708
**B76**	**0.281338**	**0.000235**	**4.718662**	**50.04068**
H77	-0.55714	0.000035	1.557143	72.07182
**B78**	**0.795071**	**-0.00016**	**4.204929**	**43.79326**
H79	-0.58034	0.000055	1.580339	79.75017
B80	0.804651	0.000292	4.195349	43.00577
H81	-0.55496	0.000024	1.554958	81.64129
B82	0.945164	-8.2E-05	4.054836	40.93877
H83	-0.55996	0.000031	1.55996	81.69504
B84	0.805103	0.000355	4.194897	43.0191
H85	-0.55471	0.000025	1.554714	81.60605
B86	0.525959	0.000013	4.474041	47.81475
H87	-0.55738	0.000031	1.557377	82.35766
B88	0.528886	0.000062	4.471114	47.81392
H89	-0.55629	0.000028	1.556293	82.29796
B90	0.548328	-0.00024	4.451672	48.45275
H91	-0.54608	0.000027	1.546081	81.82035

Table S31. Selected QTAIM BCP data for **7**.

BCP	ρ(r)	$\nabla^2\rho(r)$	ε	G(r)	V(r)
**Zn1 - P2**	**0.064864**	**0.075908**	**0.020747**	**0.039212**	**-0.05945**
**Zn1 - P3**	**0.042641**	**0.053607**	**0.02229**	**0.022716**	**-0.03203**
C4 - B80	0.123209	0.020741	2.429229	0.106622	-0.20806
H5 - C8	0.006344	0.018045	1.115227	0.003712	-0.00291
C6 - B84	0.123066	0.021103	2.477639	0.106512	-0.20775
H7 - C17	0.00711	0.020383	0.333846	0.004188	-0.00328
B82 - H83	0.175748	-0.27798	0.01793	0.111535	-0.29257
C4 - C6	0.214821	-0.34218	0.292286	0.070762	-0.22707
P2 - C8	0.154546	-0.28153	0.142908	0.073975	-0.21833
C8 - C9	0.293047	-0.74749	0.181146	0.097811	-0.38249
C9 - C11	0.297789	-0.7805	0.188366	0.09839	-0.39191
C11 - C13	0.299515	-0.79289	0.186357	0.098096	-0.39442
C8 - C17	0.291638	-0.7403	0.179667	0.096762	-0.3786
C13 - C15	0.298885	-0.79029	0.183319	0.097476	-0.39252
C15 - C17	0.298639	-0.78404	0.192977	0.099225	-0.39446
C21 - C23	0.293679	-0.75158	0.182699	0.097101	-0.3821
P2 - C23	0.156355	-0.24814	0.026851	0.086986	-0.23601
C19 - C28	0.299384	-0.79189	0.185165	0.098094	-0.39416
C19 - C21	0.297806	-0.78229	0.183174	0.097686	-0.39094
C24 - C26	0.298904	-0.78576	0.192558	0.099188	-0.39482
C23 - C24	0.291493	-0.74116	0.177008	0.095873	-0.37704
P2 - C30	0.156038	-0.26245	0.101602	0.08255	-0.23071
C26 - C28	0.298249	-0.78719	0.181654	0.096954	-0.3907
H10 - C30	0.009867	0.032834	4.63748	0.006674	-0.00514
C30 - C31	0.291464	-0.73867	0.180326	0.096537	-0.37774
H32 - H79	0.005824	0.015085	1.031316	0.0031	-0.00243
C31 - C33	0.298029	-0.78217	0.187091	0.098323	-0.39219
C33 - C35	0.299167	-0.79119	0.184454	0.097826	-0.39345
C35 - C37	0.298966	-0.79012	0.184596	0.097645	-0.39282
C30 - C39	0.29364	-0.7516	0.180713	0.097834	-0.38357
C37 - C39	0.298448	-0.78422	0.188436	0.09866	-0.39337
C30 - C63	0.003313	0.008763	2.173443	0.001778	-0.00137
P3 - C41	0.153278	-0.2659	0.097148	0.076553	-0.21958
C41 - C42	0.292458	-0.74585	0.175612	0.096492	-0.37945
H43 - H77	0.007959	0.022985	0.863808	0.004745	-0.00374
C42 - C44	0.298442	-0.78532	0.182062	0.098183	-0.3927
C44 - C46	0.299236	-0.79173	0.184256	0.097849	-0.39363
C46 - C48	0.298718	-0.78884	0.18592	0.097583	-0.39238
C41 - C50	0.291691	-0.74055	0.179665	0.096575	-0.37829
C48 - C50	0.297992	-0.78075	0.192038	0.098723	-0.39263
P3 - C52	0.154498	-0.27061	0.052203	0.077056	-0.22177
C52 - C53	0.292798	-0.74699	0.17926	0.097075	-0.3809
C53 - C55	0.298818	-0.78506	0.192747	0.099345	-0.39496
C52 - C61	0.294189	-0.7549	0.181979	0.097524	-0.38377
C55 - C57	0.298354	-0.78733	0.184334	0.097198	-0.39123
C57 - C59	0.299542	-0.79291	0.186243	0.09821	-0.39465
C59 - C61	0.298164	-0.78405	0.183019	0.097987	-0.39199
H62 - H79	0.007265	0.019862	0.595129	0.004102	-0.00324
P3 - C63	0.152157	-0.25119	0.159979	0.079133	-0.22106
C63 - C64	0.291214	-0.73868	0.175229	0.096076	-0.37682
C37 - C70	0.002642	0.00673	2.479179	0.001366	-0.00105
C64 - C66	0.298361	-0.78189	0.195228	0.09919	-0.39385
C66 - C68	0.298424	-0.78688	0.186598	0.097517	-0.39175
C63 - C72	0.292821	-0.74686	0.176265	0.097158	-0.38103
C68 - C70	0.299259	-0.79083	0.188969	0.098176	-0.39406
C70 - C72	0.298035	-0.78203	0.187341	0.098287	-0.39208
**Zn1 - B76**	**0.078917**	**0.044019**	**0.750083**	**0.042073**	**-0.07314**

B74 - B76	0.108942	-0.09789	3.070698	0.028764	-0.082
B74 - B86	0.112953	-0.102	3.615959	0.035236	-0.09597
B82 - B84	0.116255	-0.10252	4.681962	0.03927	-0.10417
C6 - B74	0.13609	-0.00312	0.522364	0.118702	-0.23818
B76 - B86	0.115208	-0.11185	2.797868	0.037005	-0.10197
B80 - B90	0.116224	-0.10879	3.94956	0.0373	-0.1018
C4 - B78	0.13617	-0.00564	0.537892	0.118245	-0.2379
B80 - B82	0.116144	-0.10212	4.708763	0.03919	-0.10391
B84 - B90	0.116125	-0.10827	4.003267	0.03729	-0.10165
B88 - B90	0.112945	-0.10096	3.720276	0.035406	-0.09605
B76 - B78	0.108737	-0.09742	3.096479	0.028718	-0.08179
B86 - B90	0.112875	-0.10072	3.741902	0.035388	-0.09596
B78 - B88	0.112903	-0.1021	3.590297	0.035165	-0.09585
B84 - B86	0.118324	-0.12557	2.562016	0.03572	-0.10283
C4 - B82	0.119702	-0.07761	2.87782	0.072664	-0.16473
B80 - B88	0.118062	-0.12439	2.609593	0.035748	-0.10259
B76 - B88	0.115131	-0.11162	2.780565	0.036963	-0.10183
B78 - B80	0.106702	-0.0617	11.33757	0.036577	-0.08858
B82 - B90	0.119742	-0.1236	3.09054	0.038516	-0.10793
C6 - B82	0.119946	-0.07837	2.83392	0.0728	-0.16519
B74 - B84	0.106653	-0.0609	12.58407	0.036454	-0.08813
B86 - B88	0.109792	-0.08941	4.755391	0.034339	-0.09103

[(Cb)Zn·NMe_3]$_2$; VIII

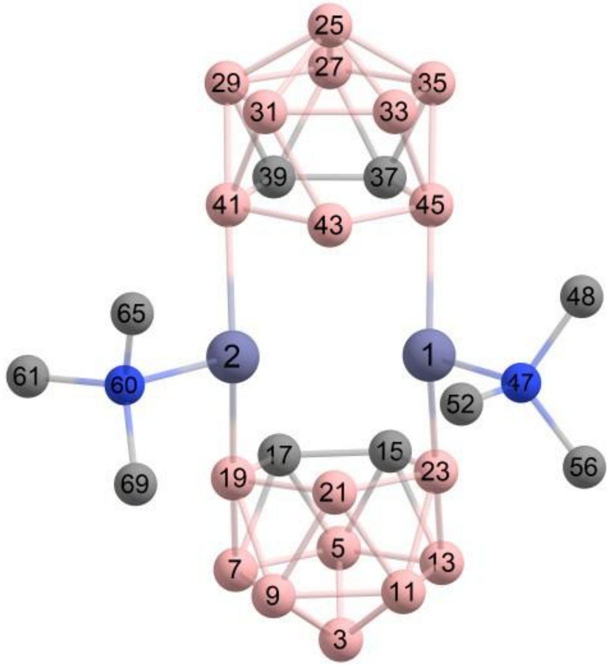

Figure S15: DFT-optimised structure of **VIII** with associated atom labels. Hydrogen atoms are omitted for clarity. Atom types are depicted by colour: carbon atoms in grey; nitrogen atoms in blue; boron atoms in pink; zinc atoms in glaucous (grey-blue-green).

'Bottom' carborane cage = CB1 = C15, C17, B19, B21, B23

'Top' carborane cage = CB2 = C37, C39, B41, B43, B45

NBO Charges

Table S32: Natural Population Analysis (NPA) charges of selected atoms of **VIII**.

Atom	NPA Charge		Atom	NPA Charge	
Zn1	1.60093		Zn2	1.59242	
N47	−0.62782		N60	−0.62443	
C15	−0.54824	Σ = −2.51886	C37	−0.54842	Σ = −2.50069
C17	−0.54303		C39	−0.54299	
B19	−0.40543		B41	−0.40678	
B21	−0.63340		B43	−0.61242	
B23	−0.38876		B45	−0.39008	

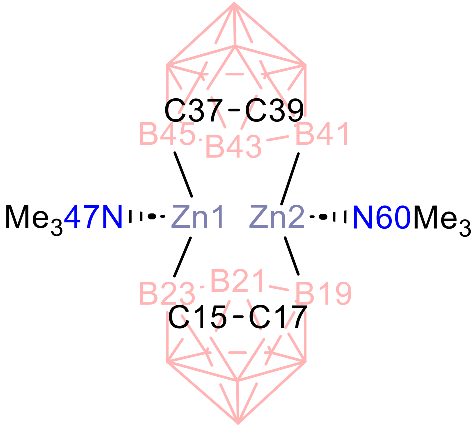

Wiberg Bond Indices

Table S33: Wiberg Bond Indices (WBI) of selected bonds in **VIII**.

Bond	WBI		Bond	WBI	
Zn1–Zn2	0.0113				
Zn1–N47	0.1409		Zn2–N60	0.1354	
Zn1–C15	0.0072		Zn2–C15	0.0051	
Zn1–C17	0.0041		Zn2–C17	0.0077	
Zn1–B19	0.0051	Σ = 0.235	Zn2–B19	0.1263	Σ = 0.2418
Zn1–B21	0.0968		Zn2–B21	0.0978	
Zn1–B23	0.1203		Zn2–B23	0.0049	
Zn2–C37	0.0052		Zn1–C37	0.0073	
Zn2–C39	0.0077		Zn1–C39	0.0040	
Zn2–B41	0.1265	Σ = 0.2433	Zn1–B41	0.0052	Σ = 0.2342
Zn2–B43	0.0988		Zn1–B43	0.0971	
Zn2–B45	0.0051		Zn1–B45	0.1206	

Second Order Perturbation Energies

There are no NBOs involving Zn1 or Zn2 with the carborane atoms (or each other). However, there are many donor-acceptor interactions ($\Delta E^{(2)}$) between the bonds of the carborane cages and both Zn atoms, only significant (over 10 kcal/mol) values are given in the table below where the accepting NBO is the first Rydberg NBO of the Zn (NBO-211 for Zn1 and NBO-289 for Zn2).

Table S34: Selected donor-acceptor interactions of **VIII**.

Donor NBO		Acceptor NBO	$\Delta E^{(2)}$ [in kcal/mol]
NBO-69	BD B21-B23	NBO-211 RY Zn1	61.26
NBO-67	BD B19-B21	NBO-289 RY Zn2	59.79
NBO-87	BD B43-B45	NBO-211 RY Zn1	64.52
NBO-85	BD B41-B43	NBO-289 RY Zn2	64.45

Laplacian ($\nabla^2\rho$) Contour Plots

There are two bond vectors to each Zn atom, one from each CB cage, the BCPs are Zn1-B23 (CB1), Zn1-B45 (CB2), Zn2-B19 (CB1) and Zn2-B41 (CB2). These are the four carbon adjacent borons.

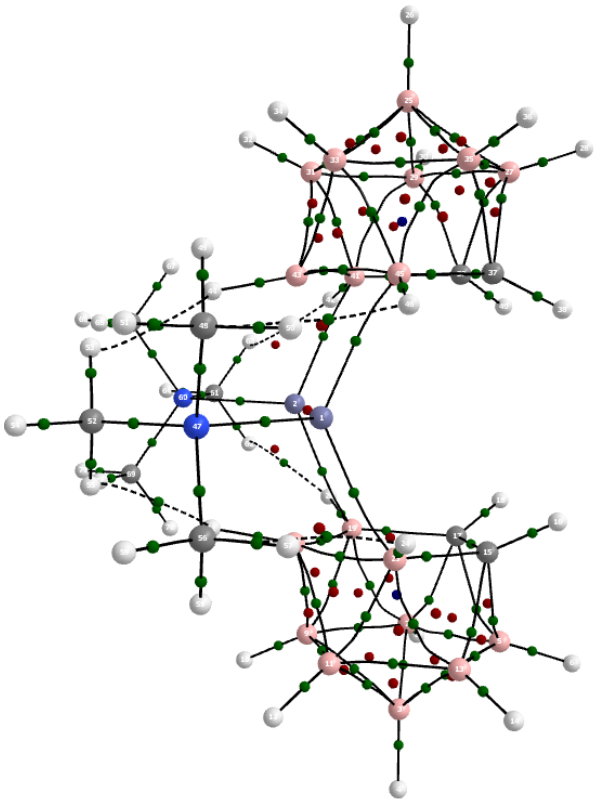

Figure S16: Side view of the DFT-optimized **VIII**, with bond paths and critical points shown. Bond critical points (BCPs) are depicted as green spheres, ring critical points (RCPs) are depicted as dark red spheres.

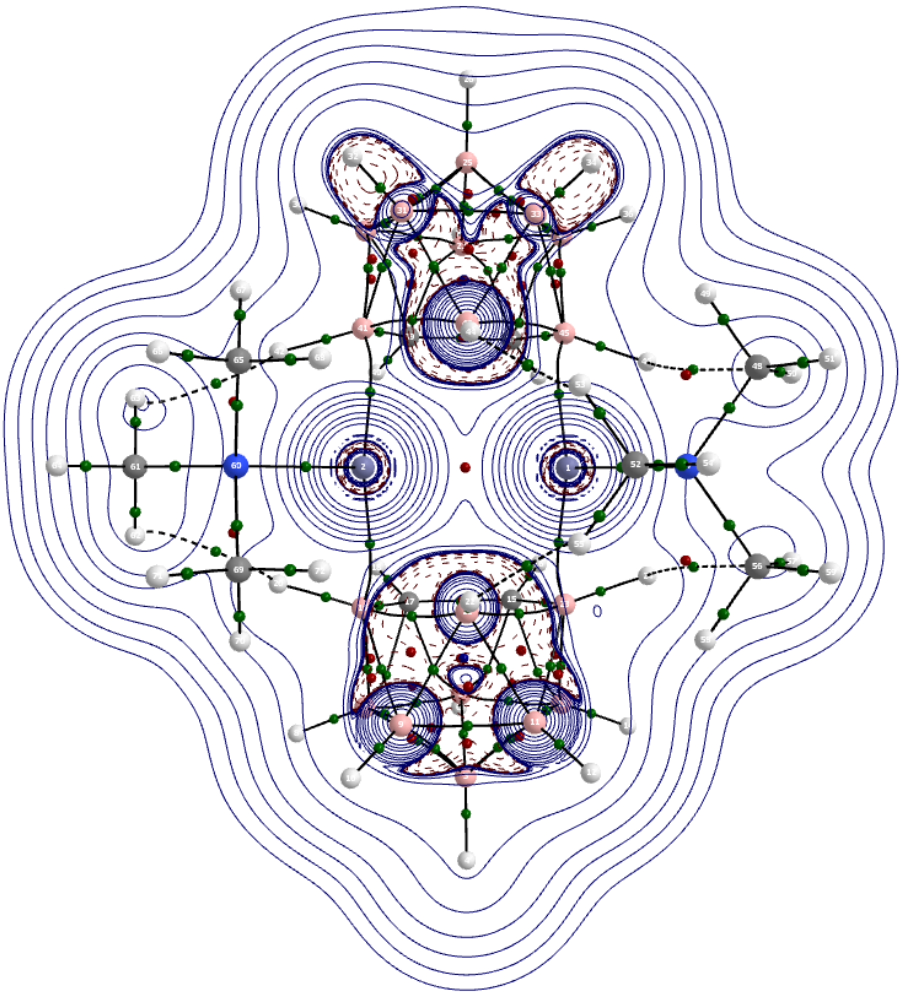

Figure S17: Contour plot of the Laplacian ($\nabla^2\rho(r)$) in the {Zn1-Zn2-B43} plane of the DFT-optimized **VIII**. Bond critical points (BCPs) are depicted as green spheres, ring critical points (RCPs) are depicted as dark red spheres.

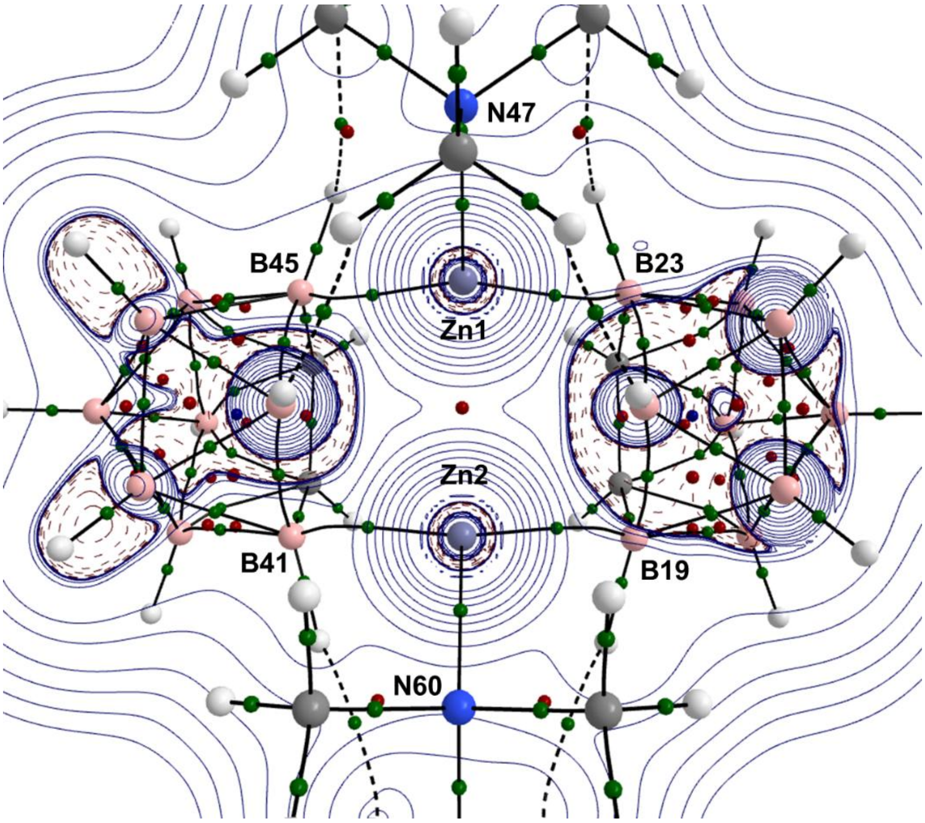

Figure S18: Contour plot of the Laplacian ($\nabla^2\rho(r)$) in the {Zn1-Zn2-B43} plane of the DFT-optimized **VIII**, zoomed in on the Zn–Zn bond and carborane cages. Bond critical points (BCPs) are depicted as green spheres, ring critical points (RCPs) are depicted as dark red spheres.

Table S35. Selected QTAIM atomic data for **VIII**.

Atom	q(A)	L(A)	N(A)	Vol(A)	%Loc(A)
**Zn1**	**0.824133**	**-0.00014**	**19.17587**	**88.06699**	**93.38423**
**Zn2**	**0.814561**	**0.000055**	**19.18544**	**90.82725**	**93.44831**
B3	0.558574	-0.00045	4.441426	48.40061	61.26974
B5	0.98914	-5.3E-05	4.01086	39.98301	63.11539
B7	0.817221	0.000088	4.182779	42.74832	62.30396
B9	0.529712	0.000064	4.470288	47.12979	61.22771
B11	0.5275	-3.9E-05	4.4725	47.32304	61.2299
B13	0.812659	-0.00037	4.187341	43.07674	62.28378
**C15**	**-1.04382**	**0.000013**	**7.043822**	**78.08018**	**69.16619**
**C17**	**-1.04738**	**-6.2E-05**	**7.047383**	**78.25518**	**69.14425**
**B19**	**0.701624**	**0.000391**	**4.298376**	**41.47167**	**62.02004**
**B21**	**0.279684**	**-9.7E-05**	**4.720316**	**47.99791**	**61.09776**
**B23**	**0.709649**	**0.000351**	**4.290351**	**41.23582**	**62.02856**
B25	0.558519	-0.00042	4.441481	48.38553	61.26967
B27	0.989296	0.000075	4.010704	40.01071	63.11588
B29	0.817495	0.000101	4.182505	42.83977	62.30552
B31	0.528995	-0.00039	4.471005	47.24307	61.22579
B33	0.527372	-4.7E-05	4.472628	47.30027	61.22992
B35	0.813139	0.000187	4.186861	42.92769	62.2864
**C37**	**-1.04385**	**-0.00013**	**7.043851**	**78.09937**	**69.16649**
**C39**	**-1.04742**	**-0.00005**	**7.047416**	**78.23816**	**69.14422**
**B41**	**0.701788**	**0.000276**	**4.298212**	**41.38177**	**62.01771**
**B43**	**0.280651**	**0.00046**	**4.719349**	**47.90947**	**61.10231**
**B45**	**0.708466**	**-0.00037**	**4.291534**	**41.2964**	**62.02376**
**N47**	**-0.91699**	**-8E-06**	**7.916993**	**60.95557**	**75.58866**
C48	0.225655	-0.00023	5.774345	62.47885	66.02595
C52	0.228145	0.00005	5.771855	63.60699	65.95276
C56	0.225866	-3.3E-05	5.774134	62.48593	66.02521
**N60**	**-0.9181**	**-0.00004**	**7.918098**	**60.83739**	**75.59422**
C61	0.225866	0.000314	5.774134	63.11188	65.9946
C65	0.230735	0.000024	5.769265	62.57207	65.98533
C69	0.23059	-0.00014	5.76941	62.58763	65.9854

Table S36. Selected QTAIM BCP data for **VIII**.

BCP	ρ(r)	$\nabla^2\rho(r)$	ε	G(r)	V(r)
B3 - H4	0.173447	-0.25723	0.008732	0.113078	-0.29046
B3 - B5	0.116088	-0.10919	3.829879	0.036799	-0.10089
**Zn2 - B19**	**0.064672**	**0.061537**	**1.475741**	**0.036199**	**-0.05701**
B19 - H20	0.156353	-0.13303	0.143236	0.117865	-0.26899
B3 - B7	0.116872	-0.11414	3.258344	0.037094	-0.10272
B5 - B7	0.115685	-0.10161	4.981249	0.03912	-0.10364
B5 - C17	0.121149	-0.07196	2.633535	0.077459	-0.17291
B7 - B9	0.116914	-0.11535	3.190512	0.036072	-0.10098
B7 - C17	0.12115	-0.01952	3.010036	0.093151	-0.19118
B7 - B19	0.10967	-0.07268	7.404329	0.037678	-0.09353
B21 - B23	0.112155	-0.12095	1.559111	0.028707	-0.08765
B11 - B21	0.106935	-0.07853	4.425379	0.035225	-0.09008
B3 - B9	0.113841	-0.1029	3.805836	0.03596	-0.09764
B19 - B21	0.111778	-0.11918	1.655848	0.028618	-0.08703
H10 - H70	0.005341	0.015612	0.683739	0.003147	-0.00239
B9 - B11	0.112125	-0.10339	3.13568	0.033826	-0.0935
B9 - B19	0.115133	-0.11108	2.937809	0.035597	-0.09896
**Zn1 - B23**	**0.064699**	**0.064976**	**1.859255**	**0.036886**	**-0.05753**
B9 - B21	0.107586	-0.08153	4.124905	0.035366	-0.09111
B3 - B11	0.1141	-0.10381	3.723784	0.03604	-0.09803
B3 - B13	0.116705	-0.11318	3.314757	0.037056	-0.10241
H12 - H58	0.005592	0.015845	0.50755	0.003217	-0.00247
B5 - B13	0.115617	-0.10181	4.847221	0.039071	-0.1036
B5 - C15	0.121507	-0.07215	2.523655	0.078065	-0.17417
B11 - B13	0.117518	-0.11675	3.209165	0.036341	-0.10187
B11 - B23	0.115705	-0.11235	2.947614	0.035678	-0.09944
B13 - C15	0.120288	-0.02307	3.306327	0.090959	-0.18769
B13 - B23	0.110625	-0.0757	7.03421	0.037491	-0.09391
C15 - C17	0.220078	-0.36757	0.284214	0.072362	-0.23662
C17 - B19	0.147323	-0.02469	0.446105	0.128067	-0.26231
C15 - B23	0.148408	-0.02477	0.431929	0.129478	-0.26515
H20 - H62	0.009066	0.025308	0.555181	0.00542	-0.00451
H24 - C56	0.008141	0.028286	1.969095	0.005761	-0.00445
B25 - B33	0.114108	-0.10384	3.722501	0.036044	-0.09805
B25 - B27	0.116093	-0.1092	3.829984	0.036801	-0.1009
**Zn2 - B41**	**0.064699**	**0.061345**	**1.474908**	**0.036163**	**-0.05699**
B25 - B29	0.116883	-0.11419	3.256605	0.037096	-0.10274
B27 - B29	0.115685	-0.10159	4.985715	0.039121	-0.10364
B27 - C39	0.121143	-0.072	2.634369	0.077435	-0.17287
B29 - B31	0.116905	-0.11533	3.189787	0.036068	-0.10097
B29 - C39	0.121169	-0.01933	3.003366	0.093229	-0.19129
B29 - B41	0.109647	-0.0726	7.424652	0.037676	-0.0935
B29 - H30	0.176084	-0.28408	0.030367	0.110612	-0.29224
B43 - B45	0.112185	-0.12108	1.55544	0.028708	-0.08769
B33 - B43	0.106929	-0.07847	4.436182	0.03523	-0.09008
B25 - B31	0.113837	-0.10287	3.80944	0.035961	-0.09764
B41 - B43	0.111755	-0.11908	1.659982	0.028617	-0.087
H32 - H67	0.005308	0.015551	0.712973	0.003132	-0.00238
B31 - B33	0.11214	-0.10345	3.132774	0.03383	-0.09352
B31 - B41	0.115121	-0.11106	2.936325	0.035589	-0.09894
**Zn1 - B45**	**0.064681**	**0.065184**	**1.853306**	**0.036935**	**-0.05758**
B31 - B43	0.107591	-0.08156	4.121047	0.035363	-0.09112
B25 - B35	0.116705	-0.11317	3.315115	0.037058	-0.10241
H34 - H49	0.005612	0.015884	0.500665	0.003226	-0.00248
B27 - B35	0.115617	-0.10183	4.839485	0.039067	-0.10359
B27 - C37	0.121503	-0.07217	2.522932	0.078056	-0.17415
B33 - B35	0.117521	-0.11674	3.211168	0.036345	-0.10188

B35 - C37	0.120267	-0.02323	3.313622	0.090884	-0.18757
B35 - B45	0.110644	-0.07577	7.016286	0.037494	-0.09393
C37 - C39	0.220072	-0.36755	0.284191	0.072358	-0.2366
C39 - B41	0.147262	-0.02462	0.446608	0.128002	-0.26216
C37 - B45	0.14844	-0.02483	0.431904	0.129505	-0.26522
H42 - H63	0.009056	0.025251	0.552578	0.005409	-0.00451
B43 - H44	0.159532	-0.16494	0.062424	0.115359	-0.27195
H46 - C48	0.008115	0.028336	2.28678	0.005768	-0.00445
**Zn1 - N47**	**0.065787**	**0.192373**	**0.014104**	**0.063295**	**-0.0785**
N47 - C48	0.246169	-0.57348	0.028667	0.104207	-0.35179
N47 - C52	0.248026	-0.58053	0.030859	0.103449	-0.35203
H22 - H55	0.008675	0.022524	0.189928	0.004866	-0.0041
H44 - H53	0.008737	0.022687	0.187268	0.0049	-0.00413
N47 - C56	0.246171	-0.5735	0.028667	0.104215	-0.35181
**Zn2 - N60**	**0.064306**	**0.186873**	**0.013187**	**0.061258**	**-0.0758**
N60 - C61	0.247025	-0.57563	0.032282	0.103486	-0.35088
H44 - H68	0.006976	0.020838	0.479406	0.004305	-0.0034
N60 - C65	0.247422	-0.57957	0.029663	0.104554	-0.354
N60 - C69	0.24743	-0.57962	0.029674	0.104554	-0.35401

Cartesian Coordinates and Raw Hartree Energies

1

SCF (BP86) Energy = -917.331981948
Enthalpy 0K = -916.994973
Enthalpy 298K = -916.972810
Free Energy 298K = -917.044169
Lowest Frequency = 18.6034 cm^{-1}
Second Frequency = 30.4951 cm^{-1}

```
Zn -0.39300  0.00023 -0.56768
N   2.35984 -1.08240 -0.05761
N   2.35999  1.08240 -0.05756
C  -1.89778  0.79068  1.15782
H  -1.33657  1.27961  1.96051
C  -1.89780 -0.79151  1.15721
H  -1.33661 -1.28106  1.95953
C   1.53850  0.00006 -0.18831
C   1.90551 -2.47415 -0.13708
H   0.81638 -2.48067 -0.29503
H   2.13874 -3.00421  0.79998
H   2.39719 -2.98739 -0.97879
C   3.68566 -0.69101  0.15518
C   4.79998 -1.67123  0.33356
H   5.75240 -1.14200  0.48613
H   4.91910 -2.32367 -0.54961
H   4.63772 -2.32415  1.20952
C   3.68575  0.69081  0.15522
C   4.80021  1.67086  0.33368
H   5.75259  1.14148  0.48602
H   4.63814  2.32361  1.20980
H   4.91932  2.32347 -0.54937
C   1.90585  2.47422 -0.13692
H   0.81669  2.48089 -0.29465
H   2.39743  2.98739 -0.97872
H   2.13934  3.00423  0.80010
B  -2.02987 -1.44975 -0.39160
H  -1.60927 -2.55936 -0.61542
B  -2.21858  0.00058 -1.53536
H  -1.98766  0.00105 -2.71891
B  -2.02985  1.45008 -0.39052
H  -1.60923  2.55985 -0.61349
B  -3.42094  1.43726  0.73551
H  -3.79253  2.46209  1.23031
B  -3.30881 -0.00064  1.77097
H  -3.52170 -0.00106  2.94743
B  -3.42097 -1.43775  0.73443
H  -3.79257 -2.46292  1.22851
B  -3.63409 -0.90604 -0.95365
H  -4.28594 -1.55291 -1.72527
B  -3.63406  0.90682 -0.95297
H  -4.28588  1.55430 -1.72410
B  -4.41669 -0.00011  0.38726
H  -5.60202 -0.00015  0.55969
```

2

SCF (BP86) Energy = -1074.58648634
Enthalpy 0K = -1074.139363
Enthalpy 298K = -1074.111785
Free Energy 298K = -1074.194565
Lowest Frequency = 19.3699 cm^{-1}
Second Frequency = 26.4801 cm^{-1}

```
Zn  0.64916 -0.23891 -0.48403
N  -2.03300  0.98844 -0.00137
N  -2.19062 -1.18337 -0.00556
C   2.16055  1.09443  0.92466
C   2.26138 -0.42117  1.35508
C  -1.29528 -0.15895 -0.12295
C  -3.38709  0.68960  0.19775
C  -4.47949  1.69519  0.39848
H  -5.45787  1.19315  0.35255
H  -4.47780  2.47811 -0.37659
H  -4.41115  2.19428  1.38038
C  -3.48325 -0.69002  0.19730
C  -4.68644 -1.56320  0.37580
H  -5.58239 -0.94207  0.52411
H  -4.59618 -2.22203  1.25736
H  -4.87254 -2.20268 -0.50516
C  -1.40968  2.33879 -0.15122
H  -0.32966  2.11939 -0.21817
C  -1.81853  3.00388 -1.47490
H  -1.62689  2.33239 -2.32695
H  -1.21189  3.91268 -1.61868
H  -2.87938  3.30392 -1.49171
C  -1.63293  3.22651  1.08227
H  -2.66622  3.60125  1.15695
H  -0.96930  4.10341  1.00933
H  -1.38295  2.68935  2.01165
C  -1.84903 -2.63783 -0.05253
H  -2.82690 -3.14639 -0.03983
C  -1.13695 -3.00606 -1.36321
H  -1.71115 -2.66584 -2.24031
H  -1.03198 -4.10187 -1.42072
H  -0.12153 -2.57795 -1.41369
C  -1.06422 -3.05408  1.20153
H  -0.06262 -2.59453  1.21177
H  -0.92922 -4.14813  1.20318
H  -1.60120 -2.77022  2.12173
H   1.58814  1.75233  1.58706
H   1.75033 -0.70287  2.28140
B   2.40318 -1.46633  0.04144
H   2.05970 -2.61791  0.14046
B   2.47137 -0.37940 -1.45727
H   2.21941 -0.71675 -2.58825
B   2.21702  1.30937 -0.74411
H   1.72311  2.28931 -1.24749
B   3.63129  1.68771  0.28680
H   3.94409  2.82916  0.46870
B   3.63346  0.58571  1.67798
H   3.87053  0.92023  2.80116
B   3.81523 -1.07045  1.06841
H   4.26100 -1.90153  1.80587
B   3.95681 -1.01070 -0.70789
H   4.63021 -1.80820 -1.29923
B   3.84057  0.72919 -1.19997
H   4.42975  1.17617 -2.14449
B   4.70960  0.26932  0.30538
H   5.89626  0.38113  0.42476
```

3

SCF (BP86) Energy = -1693.91327163
Enthalpy 0K = -1693.200198

Enthalpy 298K = -1693.157024
Free Energy 298K = -1693.275502
Lowest Frequency = 17.6646 cm^{-1}
Second Frequency = 28.0850 cm^{-1}

Zn 0.00877 1.10613 -0.00285
N -1.63919 -1.36549 0.00171
N 0.46170 -1.91178 0.00192
C -0.37780 -0.82863 0.00029
C -1.58783 -2.75721 0.00421
H -2.48961 -3.36285 0.00564
C -0.26203 -3.10270 0.00432
H 0.22983 -4.07122 0.00584
C -2.86917 -0.58843 0.00102
C -3.44223 -0.23450 1.24817
C -4.64015 0.50504 1.21376
H -5.10957 0.80262 2.15679
C -5.23313 0.87106 -0.00020
H -6.16115 1.45180 -0.00067
C -4.64035 0.50270 -1.21353
H -5.10994 0.79843 -2.15706
C -3.44245 -0.23697 -1.24669
C -2.83224 -0.63192 2.59255
H -1.86901 -1.13674 2.39826
C -3.74178 -1.64065 3.33300
H -3.27562 -1.95278 4.28332
H -3.92735 -2.54499 2.72836
H -4.72158 -1.19213 3.57208
C -2.53421 0.60262 3.47279
H -1.85753 1.30885 2.96491
H -2.05732 0.28768 4.41682
H -3.45909 1.14519 3.73401
C -2.83291 -0.63732 -2.59042
H -1.86970 -1.14188 -2.39533
C -3.74283 -1.64752 -3.32838
H -3.92836 -2.55048 -2.72166
H -3.27702 -1.96184 -4.27815
H -4.72263 -1.19937 -3.56814
C -2.53494 0.59523 -3.47343
H -3.45976 1.13742 -3.73557
H -2.05839 0.27814 -4.41692
H -1.85800 1.30249 -2.96730
C 1.91142 -1.82515 0.00100
C 2.58323 -1.78571 1.24884
C 3.98675 -1.66697 1.21427
H 4.54242 -1.62783 2.15661
C 4.67978 -1.59854 -0.00082
H 5.76979 -1.49714 -0.00153
C 3.98528 -1.66839 -1.21499
H 4.53981 -1.63040 -2.15805
C 2.58172 -1.78726 -1.24771
C 1.85904 -1.92460 2.58960
H 0.77180 -1.90822 2.39640
C 2.19350 -3.28990 3.23798
H 1.94044 -4.13239 2.57178
H 1.63175 -3.41587 4.17938
H 3.26891 -3.36397 3.47543
C 2.16851 -0.76231 3.55836
H 3.24394 -0.70714 3.80012
H 1.62583 -0.91034 4.50761
H 1.85928 0.20941 3.14092
C 1.85598 -1.92823 -2.58741
H 0.76897 -1.91185 -2.39297
C 2.18999 -3.29441 -3.23418
H 3.26514 -3.36857 -3.47277
H 1.62716 -3.42190 -4.17473
H 1.93790 -4.13595 -2.56641
C 2.16399 -0.76728 -3.55823
H 1.85503 0.20496 -3.14182
H 1.62024 -0.91681 -4.50663
H 3.23913 -0.71217 -3.80134
C 2.13060 2.08881 -0.79011
H 2.74627 1.32477 -1.27367
C 2.13129 2.08813 0.78469
H 2.74731 1.32357 1.26701
B 0.68020 2.63606 1.44170
H 0.35181 2.29497 2.55111
B -0.36570 3.14014 -0.00082
H -1.56744 3.24463 -0.00026
B 0.67872 2.63740 -1.44525
H 0.34934 2.29764 -2.55478
B 0.58179 4.33924 -0.90663
H 0.01910 5.17729 -1.55551
B 0.58278 4.33847 0.90499
H 0.02066 5.17594 1.55512
B 2.14621 3.66654 1.43424
H 2.72027 3.88644 2.46197
B 3.11096 3.27625 -0.00258
H 4.30114 3.15919 -0.00323
B 2.14479 3.66776 -1.43814
H 2.71772 3.88866 -2.46628
B 2.08588 4.72259 -0.00149
H 2.57704 5.81536 -0.00135

$[Zn(CB)_2]^-$ of **4** and **5**
SCF (BP86) Energy = -840.533912219
Enthalpy 0K = -840.222863
Enthalpy 298K = -840.203074
Free Energy 298K = -840.267949
Lowest Frequency = 21.9560 cm^{-1}
Second Frequency = 51.4408 cm^{-1}

Zn -0.00003 -0.64281 -0.00000
C -2.27684 0.28508 1.42212
C -1.96085 1.37443 0.35050
H -1.98371 0.52369 2.44940
H -1.46948 2.28080 0.71707
B -2.18574 -1.24695 0.83012
H -1.94927 -2.17801 1.56379
B -1.82953 -1.07191 -0.94985
H -1.40333 -1.93097 -1.69139
B -1.60261 0.73151 -1.12221
H -0.92460 1.34371 -1.91423
B -3.20073 1.51971 -0.82132
H -3.46871 2.58499 -1.31591
B -3.60005 1.26223 0.88602
H -4.07639 2.10588 1.59939
B -3.78724 -0.48074 1.15340
H -4.47713 -0.84193 2.07280
B -3.50063 -1.32325 -0.37873
H -4.08683 -2.34227 -0.65488
B -3.12578 -0.05693 -1.63120
H -3.45131 -0.18116 -2.78731
B -4.36775 0.25923 -0.35851
H -5.54257 0.35620 -0.61206
C 1.96116 1.37428 -0.35158
C 2.27700 0.28399 -1.42230
H 1.47002 2.28046 -0.71893

```
H   1.98401  0.52182 -2.44980
B   1.60269  0.73269  1.12164
H   0.92471  1.34568  1.91306
B   1.82918 -1.07087  0.95084
H   1.40277 -1.92923  1.69306
B   2.18549 -1.24751 -0.82900
H   1.94891 -2.17916 -1.56190
B   3.78719 -0.48195 -1.15282
H   4.47704 -0.84409 -2.07188
B   3.60038  1.26129 -0.88690
H   4.07696  2.10424 -1.60095
B   3.20101  1.52029  0.82020
H   3.46923  2.58593  1.31388
B   3.12566 -0.05564  1.63138
H   3.45108 -0.17901  2.78762
B   3.50026 -1.32311  0.38001
H   4.08621 -2.34204  0.65702
B   4.36778  0.25914  0.35850
H   5.54261  0.35602  0.61202
```

6

SCF (BP86) Energy = -1564.32634074
Enthalpy 0K = -1563.834860
Enthalpy 298K = -1563.801625
Free Energy 298K = -1563.897550
Lowest Frequency = 22.1106 cm^{-1}
Second Frequency = 22.4518 cm^{-1}

```
Zn  0.00002 -1.33886 -0.20640
Zn  0.00000  1.33859 -0.20685
N   0.00030 -2.56416  1.50531
N  -0.00058  2.56515  1.50385
C  -2.03301 -0.77431 -2.61468
H  -1.56852 -1.26715 -3.47362
C  -2.03274  0.77082 -2.61549
H  -1.56806  1.26257 -3.47495
C   2.03358  0.77244 -2.61504
H   1.56932  1.26460 -3.47451
C   2.03352 -0.77269 -2.61475
H   1.56924 -1.26514 -3.47404
C   1.16958 -2.96782  2.06038
H   2.08330 -2.60886  1.57445
C   1.20901 -3.79284  3.18989
H   2.17451 -4.09562  3.60373
C   0.00054 -4.21362  3.76491
H   0.00063 -4.86082  4.64767
C  -1.20805 -3.79247  3.19041
H  -2.17347 -4.09495  3.60467
C  -1.16887 -2.96748  2.06088
H  -2.08269 -2.60828  1.57531
C  -1.16997  2.96907  2.05850
H  -2.08359  2.60973  1.57268
C  -1.20961  3.79481  3.18747
H  -2.17519  4.09777  3.60100
C  -0.00125  4.21607  3.76238
H  -0.00151  4.86383  4.64473
C   1.20745  3.79466  3.18831
H   2.17279  4.09749  3.60249
C   1.16848  2.96893  2.05931
H   2.08240  2.60949  1.57409
B  -1.95238  1.41372 -1.11739
H  -1.56828  2.56704 -0.96433
B  -1.91127 -0.00030  0.02335
H  -1.63971  0.00028  1.20916
B  -1.95279 -1.41562 -1.11583
H  -1.56912 -2.56887 -0.96150
B  -3.49989 -1.43646 -1.99447
H  -3.93846 -2.46817 -2.41020
B  -3.51192 -0.00171 -3.03632
H  -3.90989 -0.00225 -4.16203
B  -3.49946  1.43417 -1.99604
H  -3.93768  2.46556 -2.41295
B  -3.45011  0.90163 -0.29462
H  -3.95574  1.54439  0.58682
B  -3.45036 -0.90208 -0.29361
H  -3.95619 -1.54369  0.58852
B  -4.42785 -0.00073 -1.49415
H  -5.62430 -0.00056 -1.46591
B   1.95271 -1.41450 -1.11621
H   1.56874 -2.56777 -0.96243
B   1.91101  0.00039  0.02345
H   1.63919  0.00059  1.20921
B   1.95274  1.41485 -1.11675
H   1.56871  2.56811 -0.96335
B   3.50018  1.43529 -1.99480
H   3.93873  2.46674 -2.41119
B   3.51274 -0.00022 -3.03560
H   3.91110 -0.00045 -4.16118
B   3.50011 -1.43536 -1.99428
H   3.93865 -2.46699 -2.41024
B   3.45006 -0.90158 -0.29326
H   3.95547 -1.54358  0.58884
B   3.45011  0.90214 -0.29361
H   3.95554  1.54446  0.58826
B   4.42814  0.00002 -1.49311
H   5.62458 -0.00000 -1.46445
```

VIII

SCF (BP86) Energy = -1416.69364242
Enthalpy 0K = -1416.138598
Enthalpy 298K = -1416.103904
Free Energy 298K = -1416.199866
Lowest Frequency = 26.2020 cm^{-1}
Second Frequency = 30.5549 cm^{-1}

```
Zn -0.00187 -1.39347  0.19096
Zn  0.00190  1.33541  0.23238
B  -4.39698 -0.01903 -1.18965
H  -5.59349 -0.03471 -1.18923
B  -3.44644  0.06742 -2.70971
H  -3.81819  0.11528 -3.84325
B  -3.48187  1.45107 -1.60119
H  -3.92755  2.49564 -1.97544
B  -3.46059  0.83774  0.07483
H  -3.99370  1.42988  0.97327
B  -3.43436 -0.96385 -0.01272
H  -3.94619 -1.65287  0.82771
B  -3.43549 -1.41500 -1.73658
H  -3.84523 -2.43182 -2.21402
C  -1.96377 -0.69726 -2.29001
H  -1.46991 -1.14126 -3.15910
C  -1.99110  0.84578 -2.21914
H  -1.51888  1.38568 -3.04494
B  -1.95272  1.41295 -0.68925
H  -1.60586  2.56702 -0.48128
B  -1.91155 -0.05265  0.38043
H  -1.67929 -0.10155  1.57444
B  -1.90975 -1.40710 -0.82503
```

```
H  -1.50130 -2.55833 -0.72884
B   4.39668 -0.02772 -1.19058
H   5.59314 -0.04762 -1.19064
B   3.44585  0.06419 -2.71011
H   3.81729  0.11231 -3.84375
B   3.48662  1.44613 -1.59962
H   3.93587  2.48964 -1.97249
B   3.46386  0.83051  0.07558
H   3.99940  1.41952  0.97460
B   3.43129 -0.97076 -0.01457
H   3.94095 -1.66288  0.82465
B   3.43007 -1.41954 -1.73905
H   3.83597 -2.43713 -2.21811
C   1.96057 -0.69564 -2.29077
H   1.46456 -1.13660 -3.16019
C   1.99354  0.84721 -2.21783
H   1.52302  1.38990 -3.04270
B   1.95769  1.41241 -0.68695
H   1.61529  2.56731 -0.47700
B   1.91180 -0.05479  0.38054
H   1.67984 -0.10435  1.57452
B   1.90484 -1.40730 -0.82684
H   1.49259 -2.55748 -0.73218
N  -0.00171 -2.75228  1.89051
C   1.21408 -3.61392  1.84521
H   2.11626 -2.98370  1.83706
H   1.20218 -4.22054  0.92740
H   1.23967 -4.28878  2.72363
C   0.00232 -1.98432  3.16579
H   0.90266 -1.35284  3.20974
H   0.00199 -2.67489  4.03244
H  -0.89478 -1.34843  3.21267
C  -1.22145 -3.60854  1.84985
H  -1.21551 -4.21565  0.93233
H  -2.12086 -2.97436  1.84447
H  -1.24697 -4.28281  2.72872
N   0.00186  2.75634  1.89484
C   0.00041  4.13177  1.32160
H  -0.89739  4.27290  0.70068
H   0.89653  4.27383  0.69844
H   0.00104  4.88676  2.13267
C   1.22109  2.58599  2.73188
H   1.25504  3.35526  3.52886
H   2.11959  2.66876  2.10205
H   1.21248  1.58861  3.19586
C  -1.21544  2.58448  2.73435
H  -2.11534  2.66614  2.10636
H  -1.24870  3.35371  3.53141
H  -1.20462  1.58713  3.19836
```

7

SCF (BP86) Energy = -1936.86516100
Enthalpy 0K = -1936.174161
Enthalpy 298K = -1936.127172
Free Energy 298K = -1936.258015
Lowest Frequency = 9.8304 cm^{-1}
Second Frequency = 19.5653 cm^{-1}

```
Zn -0.42311  1.13728  0.07302
P  -1.69124 -0.93217 -0.01168
P   2.05211  0.15709  0.03626
C  -2.64317  2.48014 -0.69381
H  -3.35647  1.80735 -1.17901
C  -2.63505  2.47471  0.86391
H  -3.34377  1.79835  1.35018
C  -3.54606 -0.90191 -0.18250
C  -4.17290 -1.00657 -1.44082
H  -3.57456 -1.16128 -2.34357
C  -5.57110 -0.91993 -1.53910
H  -6.04788 -1.00402 -2.52098
C  -6.35198 -0.72669 -0.38915
H  -7.44118 -0.65509 -0.47031
C  -5.73121 -0.62376  0.86656
H  -6.33291 -0.47263  1.76843
C  -4.33519 -0.70913  0.97317
H  -3.86204 -0.63518  1.95768
C  -0.42960 -2.27345  3.69148
H   0.21194 -1.88886  4.49041
C  -0.61029 -1.51595  2.52174
H  -0.11615 -0.54238  2.42501
C  -1.44289 -1.99251  1.48739
C  -2.11677 -3.22408  1.65169
H  -2.80767 -3.57933  0.88038
C  -1.92623 -3.98079  2.81738
H  -2.44984 -4.93503  2.93587
C  -1.08018 -3.50867  3.83596
H  -0.94186 -4.09739  4.74865
C  -1.11060 -1.86093 -1.50998
C  -0.93190 -1.09603 -2.68573
H  -1.09400 -0.01067 -2.66705
C  -0.54255 -1.72299 -3.88020
H  -0.41404 -1.12403 -4.78745
C  -0.30975 -3.10719 -3.90465
H  -0.00041 -3.59474 -4.83489
C  -0.46130 -3.86389 -2.73143
H  -0.26390 -4.94051 -2.74281
C  -0.86289 -3.24720 -1.53631
H  -0.96494 -3.84383 -0.62573
C   2.87765  0.43243  1.68574
C   2.40386  1.49425  2.48844
H   1.56922  2.11428  2.14561
C   3.01004  1.76686  3.72527
H   2.63408  2.59427  4.33548
C   4.08637  0.98526  4.17439
H   4.55580  1.19936  5.14033
C   4.55969 -0.07321  3.38149
H   5.39985 -0.68605  3.72543
C   3.96043 -0.35124  2.14263
H   4.32994 -1.18425  1.53666
C   3.08472  1.13843 -1.16174
C   4.48303  1.25356 -1.01309
H   4.98441  0.79176 -0.15653
C   5.23226  1.97520 -1.95385
H   6.31637  2.06614 -1.82861
C   4.59375  2.58602 -3.04673
H   5.18045  3.15565 -3.77502
C   3.20241  2.47907 -3.19460
H   2.69575  2.97103 -4.03084
C   2.44625  1.76114 -2.25355
H   1.35684  1.71708 -2.35312
C   2.49961 -1.60658 -0.38307
C   2.23229 -2.64045  0.54348
H   1.80848 -2.40306  1.52424
C   2.52564 -3.97501  0.22534
H   2.32729 -4.76141  0.96141
C   3.07461 -4.29994 -1.02623
H   3.30809 -5.34131 -1.27160
C   3.32602 -3.28104 -1.95803
```

H	3.75574	-3.52339	-2.93574
C	3.04277	-1.94272	-1.64100
H	3.25665	-1.15554	-2.36988
B	-1.13129	2.78753	1.50863
H	-0.84623	2.41456	2.62199
B	-0.03423	3.17659	0.07842
H	1.17212	3.16241	0.07041
B	-1.14323	2.79817	-1.34579
H	-0.87078	2.43995	-2.46983
B	-2.44860	4.04353	-1.34426
H	-2.98175	4.34541	-2.37362
B	-3.45215	3.78936	0.09482
H	-4.64694	3.84393	0.09972
B	-2.43669	4.03284	1.52658
H	-2.96376	4.32677	2.56129
B	-0.80122	4.47936	0.99693
H	-0.11328	5.22226	1.64249
B	-0.80816	4.48660	-0.82423
H	-0.12647	5.23354	-1.47126
B	-2.23452	5.07776	0.09387
H	-2.55052	6.23378	0.09939
